# The quality of medical products for cardiovascular diseases: a gap in global cardiac care

**DOI:** 10.1136/bmjgh-2021-006523

**Published:** 2021-09-14

**Authors:** Ngan Thi Do, Konnie Bellingham, Paul N Newton, Céline Caillet

**Affiliations:** 1 Lao-Oxford-Mahosot Hospital-Wellcome Trust Research Unit, Microbiology Laboratory, Mahosot Hospital, Vientiane, Lao People's Democratic Republic; 2 Centre for Tropical Medicine and Global Health, Nuffield Department of Medicine, University of Oxford, Oxford, UK; 3 Infectious Diseases Data Observatory/WorldWide Antimalarial Resistance Network, Centre for Tropical Medicine and Global Health, University of Oxford, Oxford, UK

**Keywords:** systematic review, epidemiology, public health, cardiovascular disease

## Abstract

**Objective:**

Good quality cardiovascular medicines and devices are crucial in the prevention and management of the ever-growing threats of cardiovascular diseases (CVDs) globally. Yet our current understanding of the extent and impact of substandard and falsified (SF) cardiovascular medical products is poor. Our objective was to review the available literature on SF cardiovascular medicines/devices, with a focus on prevalence studies to discuss their impacts on public health.

**Methods:**

Searches were conducted in Embase, PubMed, Web of Science, Google Scholar, Google and websites with interest in medicines/devices quality up to 31 August 2020. Articles in English and French identified in these searches were screened for eligibility. The Medicine Quality Assessment Reporting Guidelines was used to assess the quality of prevalence surveys, and we report according to the Preferred Reporting Items for Systematic Reviews and Meta-Analyses statement.

**Results:**

A total of 279 articles were included, which were subcategorised into prevalence surveys (n=28), equivalence studies (n=118), stability studies (n=5), routine quality control analyses (n=15), bioavailability studies (n=2), recalls/seizures/case reports (n=77), general discussions (n=24) and reviews (n=10). A failure frequency (defined as the proportion of samples that failed at least one quality test described in the report) of 525 (15.4%) was observed for the 3414 samples tested for quality in the 27 prevalence surveys with sufficient information for inclusion in our quantitative analysis. Nineteen surveys (70.4%) used convenience outlet sampling. The majority (88.8%, 3032/3414) of samples included in prevalence surveys were collected from low-income and middle-income countries. The most common defects were out-of-specification active ingredient(s) content, impurity/contaminant content and impaired dissolution. We found 26 incidents describing SF cardiovascular devices with 181 related deaths but no prevalence surveys.

**Conclusion:**

The data suggest that SF cardiovascular products are likely to be a serious public health problem that has received limited attention. We do not suggest that 15.4% of cardiovascular medicines are SF, and our findings highlight the need for more research with robust methodology to provide more accurate prevalence estimates in order to inform policy and implement measures to ensure the quality of cardiovascular medicines and devices within the supply chain. Ensuring that CVD medical products are of good quality would help ensure effectiveness and that the benefits of therapy are realised in the prevention and treatment of CVDs.

**Key questions**

**What is already known?**

- Substandard and falsified (SF) medicines and medical devices for cardiovascular diseases lead to negative health impact and adverse financial consequences for patients and the community.
- Better understanding of the global extent of SF cardiovascular medical products and their burden on public health is needed.

**What are the new findings?**

- Our study showed that almost one-fifth of the 4703 samples tested for quality were substandard or falsified; however, this estimate is not globally generalisable due to the limited data and methodologies of the included studies.
- Issues and incidents of SF cardiovascular medical devices were linked to the death of 181 patients; however, our study did not identify any prevalence surveys relating to cardiovascular medical device quality.

**What do the new findings imply?**

- Our findings suggest that SF cardiovascular medical products may be a serious but neglected public health problem.
- More studies, with objective methodology and better reporting, are needed to provide more accurate estimates and to better identify where and what the problems are, to better inform policy.

## Introduction

Cardiovascular diseases (CVDs) are a group of disorders of the heart and blood vessels and a leading cause of death globally. In 2019, there were an estimated 523 million cases of CVDs according to the Global Burden of Disease study.^1^ Approximately 18.5 million people died from CVDs in 2019, representing 32% of all global deaths,^1^ and this is predicted to reach 23.6 million deaths by 2030 as a result of sociodemographic changes, including population ageing and increasingly common risk factors (eg, obesity, hypertension and diabetes).^2^ According to the WHO, three-quarters of CVD deaths take place in low-income and middle-income countries (LMICs).^3^ Of all CVD deaths, 85% are due to heart attacks and strokes. The global cost of CVDs for patients and communities is enormous. In LMICs, the mean monthly treatment for hypertension costs US$22, and that for stroke and coronary heart disease ranges between US$300 and US$1000.^4^

Prevention, control and early detection of cardiovascular risk for individual patients are vital interventions. Cardiovascular medications are key components for CVD prevention and therapy. Approximately 400 active pharmaceutical ingredients (APIs) or combinations of API for the treatment or prevention of CVDs are included in the WHO Anatomical Therapeutic Chemical (ATC) classification list,^5^ and the British National Formulary (77/2019).^6^ Thirty-two are included in the 2019 WHO Essential Medicines List (EML).^7^ Surgical operations and use of medical devices are common interventions for severe disease. The US Food and Drug Administration (FDA) Code of Federal Regulations lists 154 types of devices for CVDs.^8^

Data from 84 countries described in the World Medicines Situation Report showed that in 2008, cardiovascular medicines were the second most consumed class of medicine in the non-hospital sector.^9^ In 2017, the total revenue from branded cardiovascular medicines was over US$40 billion^10^ and is expected to reach approximately US$90 billion by 2024.^11^ The cardiovascular medical devices market was estimated to reach US$69 billion by the end of 2026.^12^

Falsified medicines are those that ‘deliberately/fraudulently misrepresent their identity, composition or source’.^13^ Substandard medicines are ‘authorised medical products that fail to meet either their quality standards or their specifications, or both’.^13^ This may result from negligence/errors during the manufacturing process or degradation through deterioration because of inappropriate storage/transport in the supply chain. There is usually inadequate evidence to distinguish poor quality medicines resulting from errors during the manufacturing process from subsequent degradation in the supply chain due to heat and humidity. Substandard or falsified (SorF) medical products of all therapeutic classes have been found worldwide. Many describe the issue as a ‘pandemic’.^14^ In 2018, 159 diverse signatories of the Oxford Statement called ‘for investment, policy change, and action to eliminate substandard and falsified medical products’.^15^ A 2017 WHO report, based on 100 studies published between 2007 and 2016, found that 10.5% of the 48 000 analysed medical products, for all classes, collected in 88 LMICs, failed at least one quality test.^16^

Cardiovascular medicines do not seem to be an exception to the SF challenges. Falsified Plavix (clopidogrel) containing simvastatin was identified in the UK in 2007,^17^ and in 2008, the USA and Germany faced issues of contaminated heparin, with dire consequences for a large number of patients.^18^ In 2011–2012, over 200 patients died in Lahore (Pakistan) after using Isotab (isosorbide mononitrate), which was found to contain deadly amounts of pyrimethamine.^19^ Between 2013 and 2017, ‘heart medicines’ made up to 5.1% of all the SF cases (75 out of 1500 reports) reported to the WHO Global Surveillance and Monitoring System, but details regarding the incidents and countries of occurrence are unavailable.^20^ The recent large SEVEN study surveyed the quality of seven cardiovascular medicine in 10 sub-Saharan African countries, yielding results of great concern as 249 of the 1530 samples (16.3%) tested failed to meet the stated specifications.^21^ Poor quality medical devices used in CVD prevention or treatment and their impact on public health are also of concern. In 2016, almost 400 000 implantable cardioverter defibrillators (ICDs) and cardiac resynchronisation therapy devices (CRT-Ds) were recalled due to premature battery depletion that resulted in multiple adverse reactions and the death of at least two people.^22^ This systematic review was conducted with the key objective to summarise the available literature on cardiovascular medicines/devices quality globally, with a focus on prevalence studies, in order to discuss their potential impact on public health and inform policy.

## Methods

### Search strategy

Search terms relevant to pharmaceutical quality (eg, ‘falsified’ and ‘substandard’) were combined with the names of API used for the prevention or treatment of CVDs and the main classes of cardiovascular medicine (eg, ‘beta blocker’ and ‘anticoagulant’) (online supplemental material 1). The names of APIs and medicine classes were retrieved from the WHO ATC database (online supplemental material 1). Systematic searches were conducted in Embase, PubMed and Web of Science in English up to 31 August 2020. The search terms were adapted for searches in Google, Google Scholar, national medicines regulatory authority (MRA) websites and other websites with interest on medicine quality in English and French (online supplemental material 2). Only articles from the first 10 pages (20 titles/page) of Google search results were screened for eligibility. Titles and abstracts were first screened and full texts of the identified articles were then assessed for eligibility. A manual search of the reference lists of the included articles was performed. Articles identified in previous systematic reviews by our research group that included cardiovascular medicines, not captured in our searches, were also included.^23 24^

10.1136/bmjgh-2021-006523.supp1 Supplementary data

10.1136/bmjgh-2021-006523.supp2 Supplementary data

### Eligibility criteria

Scientific articles and grey literature in English or French assessing or discussing the quality of cardiovascular medicines, whether they contained empirical data or not, were included. Non-empirical literature includes general discussions (eg, on the regulatory response to contaminated valsartan products) and reviews of the literature on various aspects of cardiovascular medicines’ quality (eg, review of the literature on heparin’s contaminants). Articles containing scientific data on the prevalence of cardiovascular medicine quality were the most relevant publications for this review. Other scientific articles included stability studies, equivalence studies, bioavailability studies and quality control analyses. We also included reports of seizures, recalls, alerts by MRAs or pharmaceutical companies and adverse reactions where the quality of the medicine was suspected to be the cause. The different types of publications included in this review that included data points are described in table 1.

**Table 1:** Types of studies with data points included in the review and definition

	Study/report type	Definition
Scientific reports	Quality control	Surveillance in which samples were collected to be analysed in routine postmarketing surveillance by MRA or a laboratory mandated by an MRA
	Prevalence survey	Study in which samples were collected within the pharmaceutical supply chain to assess their quality in order to describe the prevalence of circulating SF medicines
	Equivalence study	Study to assess the quality of different marketed brands of the same APIs, assuming that the results of the collected samples would represent the quality of the brand as a whole and not an estimate of the frequency of individual samples of different quality
	Stability study	Study in which quality tests are performed on medicines subjected to various storage conditions
	Bioavailability study	Study of the in vivo bioavailability, that is, testing for adequate body tissue concentration, including the rate and extent to which the drug reaches the body tissue compartment
Other reports	Recall/warning/alert	Recall/warning/alert of products by manufacturers via MRA or by MRAs directly, or by WHO rapid alert
	Case reports	Patients not responding to medicines or experiencing adverse drug reactions in which the quality of the medicine was suspected as the cause, also includes samples analysed for quality not included in a scientific study
	Seizure	Confiscations by police or MRA

API, active pharmaceutical ingredient; MRA, medicines regulatory agency; SF, substandard and falsified.

Publications on the quality of herbal/mineral/animal part remedies used to treat CVDs were not included in this review. We excluded from our quantitative analysis data from reports of whole classes of medicines with no details on the quality of cardiovascular medicines and publications describing the development/validation of analytical technique(s) for quality assessment of cardiovascular medicines.

### Key definitions

We follow in this review the latest WHO definitions of substandard and falsified (SF) medicines, published in 2017.^13^ As it is not possible to reliably classify a medicine without packaging analysis, products without packaging authentication that failed at least one quality test or the results are outside the acceptable limits of the chosen specifications reference (pharmacopoeia monograph or in-house specifications) are defined as ‘SorF’. However, samples that contained incorrect or no API were assumed to be falsified. There is a risk of misclassification of such samples as falsified when they are actually substandard, due to gross manufacturing errors.

We define ‘failure frequency’ (FF) as the proportion of samples that failed at least one quality test described in the report.

Pharmaceutical analysis relies on compendial tests such as those described in pharmacopoeial monographs. For finished medicines, monographs commonly include the identification and quantification of API content (using sophisticated standardised techniques such as liquid chromatography (LC) coupled with various detectors), dissolution testing, detection of specific levels of predetermined impurities/related substances, uniformity of dosage units and additional attributes, depending on the formulation of the product (eg, friability of tablets). In many studies included in this review, not all pharmacopoeial analyses were conducted and a variety of non-pharmacopoeial technologies were used, for example, for research on specific contaminants or for unstated APIs. Details on the techniques used were not always provided in the reports, making it difficult to standardise the definition of a ‘failing sample’. Consequently, a failing sample is defined in this review as a sample for which at least one quality analysis test performed by the investigators failed, irrespective of the number of and the nature of and the technologies used for these tests. As a wide variety of medical devices for the diagnosis, prevention, monitoring, treatment or alleviation of CVD exist and, as far as we are aware, each specific device or type of device requires customised quality tests; we follow the same terminology for medical devices (ie, a medical device is considered as failing if at least one of the quality analyses is failed).

We define a ‘data point’ as a specific location where medicines were collected for quality analysis, at a given time and for a given study.

### Data collection

Data were manually extracted into the ‘Online Medicine Quality Data Manager’, an online data entry tool developed by the Infectious Diseases Data Observatory Informatics and the Lao-Oxford-Mahosot Hospital-Wellcome Trust Research Unit Medicine Quality team. Publication type (eg, report and original research article), year of publication, publisher, sampling type, location (country and city, where available) and type of outlet where samples were collected, total number of samples collected, API/API combination name, number of samples failing medicine quality test(s), quality defect and the techniques that were used to analyse samples were entered in the online tool.

Only the data on the quality test results of the medicines before being submitted to stress conditions in stability studies were taken into account in our analysis.

### Data analysis

FlySpeed SQL Query V.3.5.4.2 was used to extract data from the online database and Microsoft Excel 2013 was used for data analysis. Qualitative variables were expressed as numbers and percentages (n (%)). Quantitative variables were expressed as median with first and third quartiles (Q1 and Q3, respectively).

### Quality of studies assessment: medicine quality assessment reporting guidelines (MEDQUARG)

The methodology and reporting of prevalence surveys were evaluated using the MEDQUARG checklist of 26 items used in reports of medicine quality surveys.^25^ All criteria had to be fulfilled for each item to be awarded one point. Prevalence surveys were assessed independently by two reviewers with a third person resolving any disagreement. Only the prevalence surveys published as original articles in scientific journals, following the Introduction/Methods/Results/Discussion or similar style and published as reports or PhD thesis, were assessed.

### Medical devices

A similar methodology as that described previously for the medicines was applied for the identification, screening, inclusion of articles (see specific key terms in online supplemental material 1), and the extraction, entry and analysis of data related to cardiovascular devices quality.

This review was registered in the International Prospective Register for Systematic Review (registration number CRD42018094426) and is reported according to the Preferred Reporting Items for Systematic reviews and Meta-Analyses guidelines (online supplemental material 3)

10.1136/bmjgh-2021-006523.supp3 Supplementary data

## Results

### Overall literature on cardiovascular medicine quality

**Figure 1:**
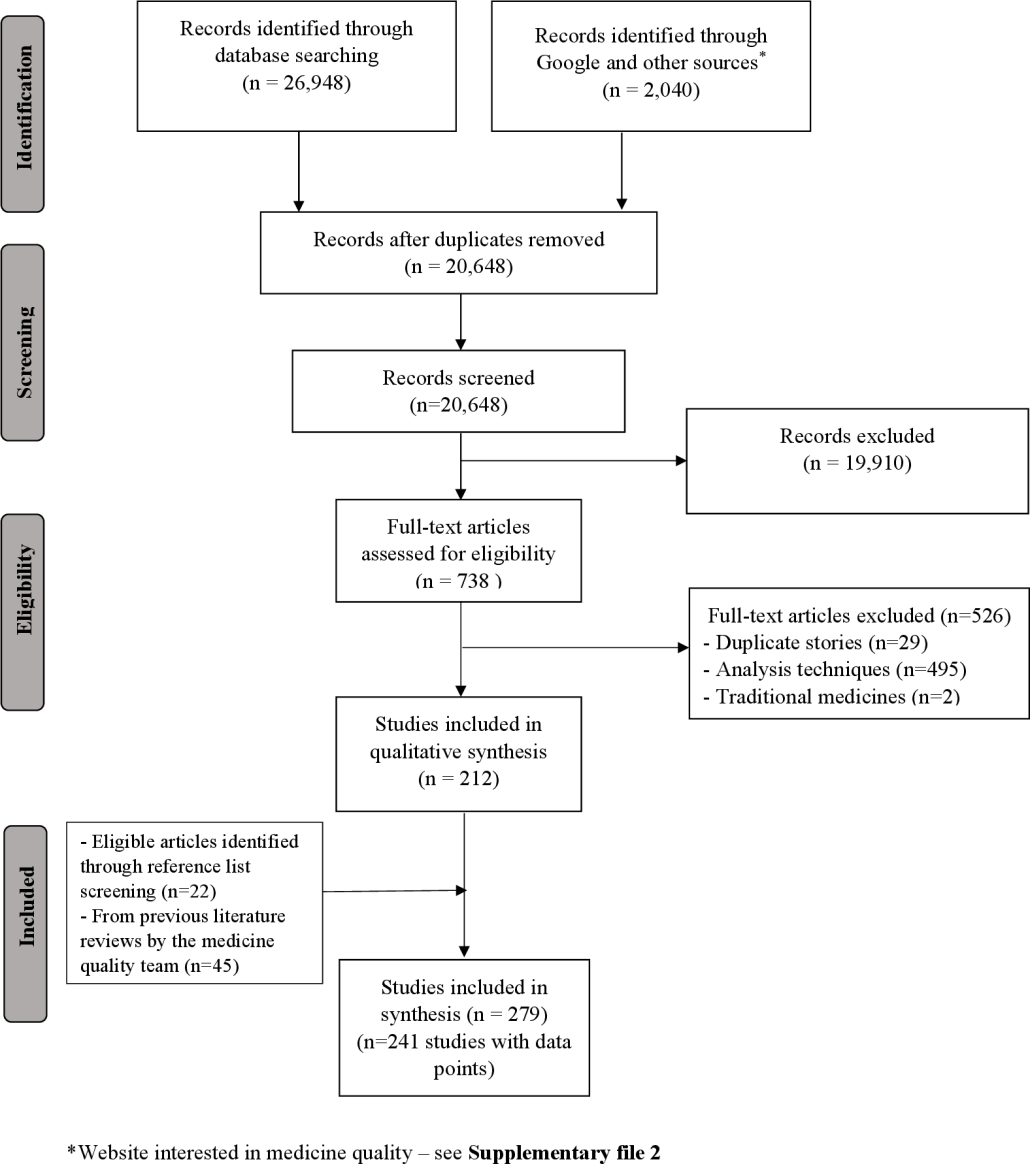
Preferred Reporting Items for Systematic Reviews and Meta-Analyses flowchart of the selection process of the publications on cardiovascular medicine quality.

After removal of duplicates, 20 648 out of 28 988 publications gathered through electronic searches were screened by title and abstract (figure 1).

**Figure 2:**
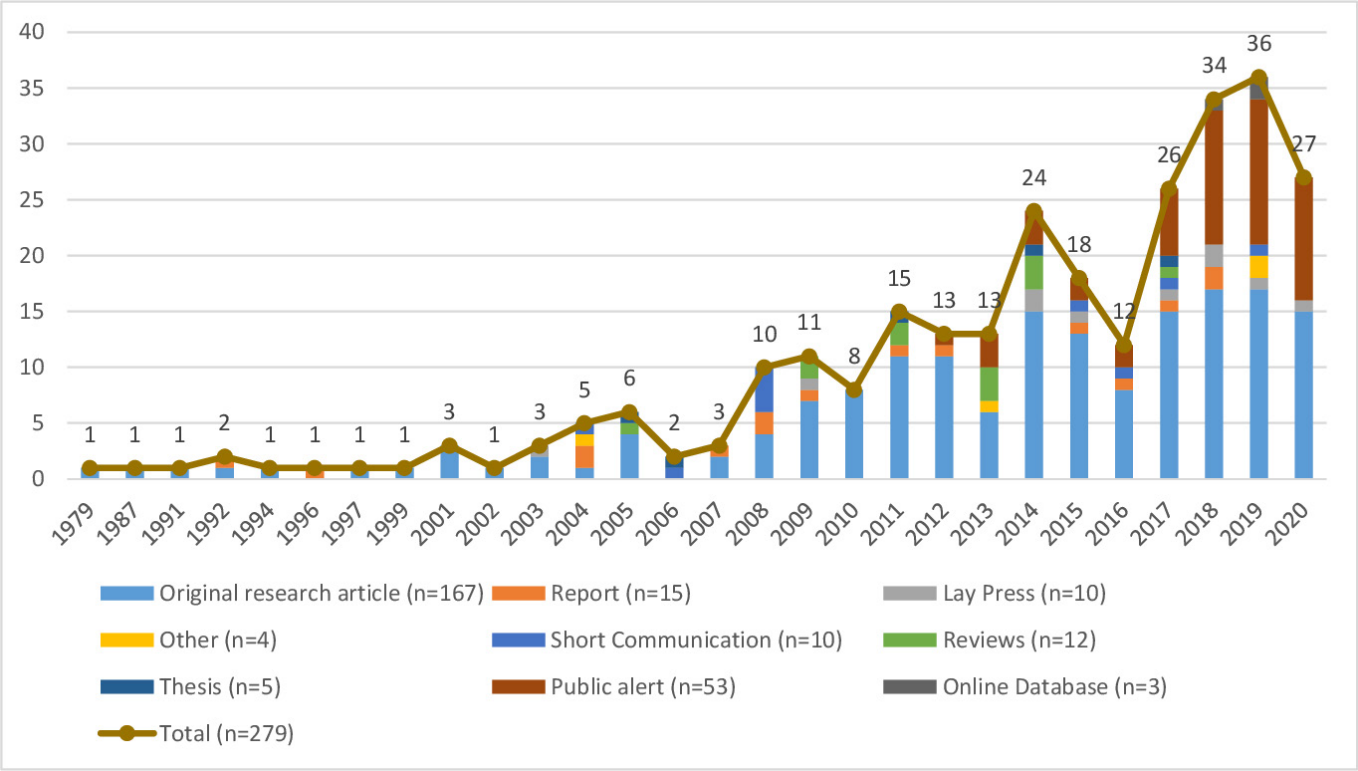
Number of publications per type and year of publication. (Note: publications published up to 31 August 2020 only were included, hence the reduction in the number of publications in 2020 compared with 2019)

Of these, 738 full-text papers were retrieved to assess eligibility with 279 publications included in this review, of which most (59.9% (n=167)) were original research articles and public alerts (19.0% (n=53)) (figure 2). Most original research articles (89.8%, 150/167) were published in peer-reviewed journals. The number of publications related to cardiovascular medicines quality per year was stable between 1979 and 2003, and then increased from five publications in 2004 to 36 in 2019.

Of the 279 publications, 38 (13.6%) did not contain any data point information, precluding inclusion in quantitative analysis. These included 24 discussions and 10 reviews, which did not include details of cardiovascular medicines’ quality, and 4 studies with insufficient details on the medicines tested (1 prevalence survey, 1 routine quality control analysis and 2 equivalence studies). A total of 241 (86.4%) publications described the quality of cardiovascular medicines in a specific location at a specific time with a total of 488 data points. Out of those 241 publications, 116 were equivalence studies (48.1%); 27 (11.2%) were prevalence surveys; 14 (5.8%) were routine quality control analyses; 5 (2.1%) were stability studies; and 2 (0.8%) bioavailability studies (online supplemental material 4). The rest were recall/warning/alerts (n=65), seizures (n=6) or case reports (n=6) published in newspapers or on medicines regulatory authorities’ websites.

10.1136/bmjgh-2021-006523.supp4 Supplementary data

A total of 4703 samples of 49 different API or combinations of API in 70 countries in five continents were collected and tested for quality and were mainly included in prevalence surveys (n=3414, 72.6%), equivalence studies (n=822, 17.5%) and MRA quality control analysis (n=443, 9.4%). Of all samples, 822 (17.5%) failed at least one quality test. Of the failing samples, 696 (84.7%) were classified as SorF because no packaging analysis to assess the authenticity of the samples was performed; 122 (14.8%) were substandard; and 4 (0.5%) were falsified.

All data are mapped in the Infectious Diseases Data Observatory Medicine Quality Surveyor system (https://www.iddo.org/mqsurveyor/%23cardiovascular).

### Prevalence studies

Twenty-seven prevalence surveys published between 1996 and 2020 (19 in the last 10 years) contained sufficient information for inclusion in our quantitative analysis (online supplemental material 5). Overall, samples of 23 different APIs or combinations of APIs were collected in 28 countries (131 data points) from 4 continents. The sample size ranged from 2 to 1530 samples with a median (Q1–Q3) of 30 (10–94) samples per survey. The overall FF in prevalence surveys was 15.4% (525/3414). Four prevalence surveys used random sampling to select the outlets to be included; 2 used mixed random and convenience sampling designs (depending on the type of outlets sampled); 19 used convenience sampling; and in 2 studies, the sampling methodology was unclear. Samples obtained through convenience sampling of outlets had an FF of 9.3% (197/2126); those using random selection had an FF of 25.2% (319/1268); and those with unclear sampling strategy had an FF of 45.0% (9/20).

10.1136/bmjgh-2021-006523.supp5 Supplementary data

In most prevalence studies (22/27, 81.5%) samples were tested for more than one quality attribute (online supplemental material 5). One-sixth of the samples tested for impurity/contaminant/related substances failed the test (16.5%, 80/484); 13.1% failed the API content test (430/3293); and 28/659 (4.2%) failed dissolution tests (online supplemental material 6). Four samples out of 1335 (0.3%) tested for packaging authenticity in prevalence surveys failed. These were falsified medicines imitating Blopress (candesartan). Of 430 samples that failed API content tests, 13.7% (n=59) contained lower and 3.7% (n=16) contained higher than the reference limits chosen by the authors, and for 82.6% (n=355), there was not enough information in the publication to determine whether they contained higher or lower amounts of API.

10.1136/bmjgh-2021-006523.supp6 Supplementary data

Over 80% of samples (2743/3293) tested for API content were analysed by LC (coupled with various detectors), and 0.5% (18/3293) were analysed by UV-visible spectrophotometry. Thin-layer chromatography was used for 50 samples for API identification and/or semiquantitation. For 532 (16.2%) samples, no information was given on the technique(s) used.

More than one reference pharmacopoeia was used in nine studies. The United States Pharmacopeia (USP) was the most commonly used (in 18 studies), followed by the British Pharmacopoeia and the Brazilian Pharmacopoeia (in seven and three studies, respectively) (online supplemental material 5). In the study with the largest sample size, an in-house validated method and in-house specifications were used.^21^

The highest FF was observed in samples collected from hospitals/health centres (44.4%, 16/36), followed by private pharmacies (19.8%, 349/1762) and unlicensed/unregistered outlets (19.7%, 129/656) (online supplemental material 7).

10.1136/bmjgh-2021-006523.supp7 Supplementary data

For 1418 samples described in 18 articles, a breakdown of the samples’ stated manufacturer origin was not given. For those 1996 samples with such data, more than 1000 samples were stated as made by European manufacturers, with an FF of 8.2% (85/1035). The FF of samples stated as made by American manufacturers was the highest (50.0%, 3/6), followed by those made by African (17.5%, 37/211) and Asian manufacturers (15.9 %, 118/744) (online supplemental material 8).

10.1136/bmjgh-2021-006523.supp8 Supplementary data

We found no publicly available evidence on cardiovascular medicine quality for 167/195 (85.6%) of nation states.^26^ More than half (65.9%, 2250/3414) of the samples in prevalence surveys were collected in middle-income countries, 22.9% (782/3414) in low-income countries and 5.3% (182/3414) in high-income countries (HICs) (table 2). Two hundred samples (5.9%) were part of a multicountry study, but the FFs were not specified by country. More than 90% of samples included in prevalence surveys were collected in Africa and Asia, representing 62.8% (2 143/3414) and 28.5% (973/3414) of all the samples, respectively. Most samples collected in Africa were part of a large single study conducted in 10 countries^21^ in which half of the total samples included in the prevalence surveys (n=1530, 44.8%) were collected. The FF was the highest in samples collected in Europe (80.0%, 8/10) followed by the Americas (43.2%, 38/88), but the total number of samples tested was low (10 and 88). The FF was 20.3% (434/2143) for samples collected in Africa and 4.4% (43/973) in Asia. The largest number of samples was collected in India (n=521), with an FF of 0.6%.

**Table 2:** FF by continent/country in prevalence surveys of cardiovascular medicine quality

Continent	Country	Income	Publications (n)	Data points (n)	FF % (n/N)
Europe					80.0% (8/10)
	Belgium	HIC	1	1	80.0% (8/10)
Americas					43.2% (38/88)
	Brazil	UMIC	3	9	61.4% (35/57)
	Mexico	UMIC	2	3	60.0% (3/5)
	USA	HIC	2	2	0.0% (0/26)
Africa					20.3% (434/2143)
	Nigeria	LMIC	2	7	38.7% (179/463)
	Niger	LIC	1	7	24.0% (24/100)
	Congo	LMIC	1	7	22.0% (33/150)
	Benin	LIC	1	7	20.6% (67/325)
	Zimbabwe	LMIC	1	2	18.2% (2/11)
	DR Congo	LIC	2	12	17.9% (25/140)
	Côte d'Ivoire	LMIC	1	7	17.6% (52/295)
	Mauritania	LMIC	1	7	15.3% (23/150)
	Rwanda	LIC	1	5	12.5% (2/16)
	Burkina Faso	LIC	1	7	10.0% (14/140)
	Togo	LIC	2	9	8.3% (10/121)
	Guinea	LIC	1	5	4.0% (2/50)
	Senegal	LMIC	1	7	0.8% (1/130)
	Libya	UMIC	1	2	0.0% (0/9)
	South Africa	UMIC	1	1	0.0% (0/43)
Asia					4.3% (42/973)
	Indonesia	LMIC	1	3	100.0% (4/4)
	China	UMIC	1	1	53.8% (14/26)
	Japan	HIC	1	3	16.7% (1/6)
	Myanmar	LMIC	2	2	11.8% (2/17)
	Afghanistan	LMIC	1	1	10.0% (3/30)
	Cambodia	LIC	1	1	8.9% (7/79)
	Mongolia	LMIC	1	1	6.8% (8/118)
	India	LMIC	2	6	0.6% (3/521)
	Jordan	UMIC	1	4	0.0% (0/172)
Unknown*	Unknown	Unknown	2	2	1.5% (3/200)
Total			27	131	15.4% (525/3414)

Because of the limited number of samples tested for quality in the studies included in this review, the figures should not be interpreted as representative of the prevalence of specific substandard and falsified cardiovascular medicines (please refer to the Discussion section).

FF is defined as the proportion of samples that failed at least one quality test described in the report.

*Multicountry study (Argentina, Austria, Brazil, Canada, China, Cyprus, Denmark, Egypt, Finland, France, Germany, Greece, Hungary, Luxembourg, Mexico, Netherlands, New Zealand, Poland and Portugal) with no breakdown of the results by country.

FF, failure frequency; HIC, high-income country; LIC, low-income country; LMIC, low-income and middle-income country; UMIC, upper middle-ncome country.

The most commonly collected API in prevalence surveys was amlodipine with 832 (24.4%) samples analysed, followed by atenolol (511/3414, 15.0%) and furosemide (466/3414, 13.6%). FFs were 18.1%, 7.8% and 7.1% for amlodipine, atenolol and furosemide, respectively (table 3).

**Table 3:** FF by API/API combination in prevalence surveys of cardiovascular medicine quality

API/API combination	Publications (n)	Data points (n)	FF % (n/N)
Enalapril	1	1	100.0% (2/2)
Clopidogrel–acetylsalicylic acid	1	1	80.0% (8/10)
Nifedipine	1	1	76.5% (78/102)
Candesartan	2	6	42.6% (20/47)
Warfarin	2	4	37.5% (3/8)
Lisinopril	1	3	31.9% (53/166)
Simvastatin	3	13	28.1% (64/228)
Captopril	3	12	25.8% (63/244)
Methyldopa	2	2	20.0% (1/5)
Epinephrine	1	2	18.2% (2/11)
Amlodipine	5	16	18.1% (151/832)
Atenolol	8	17	7.8% (40/511)
Furosemide	6	14	7.1% (33/466)
Hydrochlorothiazide	5	14	1.8% (4/218)
Atorvastatin	4	6	1.5% (3/199)
Ramipril	1	1	0.0% (0/39)
Acenocoumarol	1	10	0.0% (0/165)
Clopidogrel	1	1	0.0% (0/33)
Diltiazem	1	1	0.0% (0/12)
Bisoprolol	2	2	0.0% (0/77)
Propranolol	2	2	0.0% (0/4)
Digoxin	1	1	0.0% (0/2)
Valsartan–hydrochlorothiazide	1	1	0.0% (0/33)
Total	27	131	15.4% (525/3414)

Because of the limited number of samples tested for quality in the studies included in this review, the figures should not be interpreted as representative of the prevalence of specific specific substandard and falsified cardiovascular medicines (please refer to the Discussion section).

Note: We found no data on the quality of medicines belonging to the WHO ATC peripheral vasodilator and vasoprotective subgroups.

FF is defined as the proportion of samples that failed at least one quality test described in the report.

API, active pharmaceutical ingredient; ATC, Anatomical Therapeutic Chemical; FF, failure frequency.

The FF of enalapril samples was the highest (100.0%, 2/2), followed by that of clopidogrel– acetylsalicylic acid (80.0%, 8/10) and nifedipine (76.5%, 78/102), but very few samples were tested.

**Figure 3:**
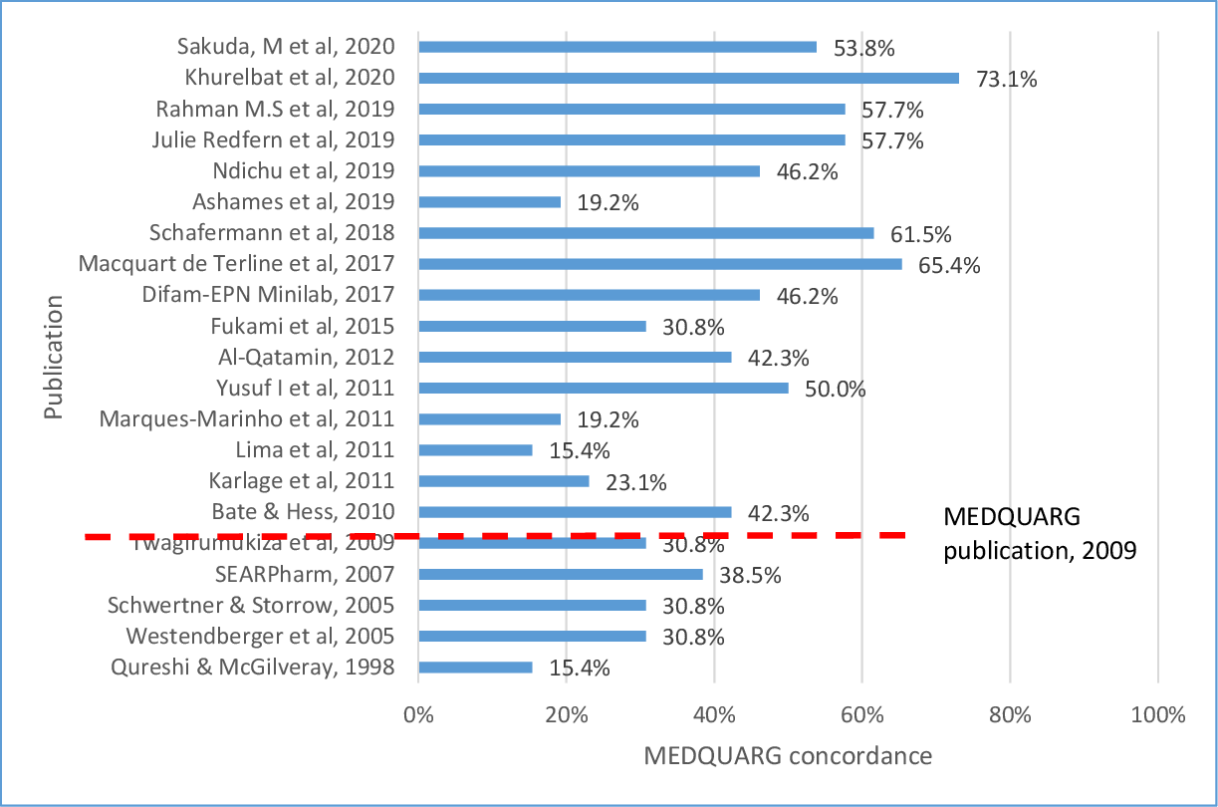
Percentage of concordance of the 21 prevalence surveys of cardiovascular medicine quality with the 26 items included in the MEDQUARG checklist. MEDQUARG, Medicine Quality Assessment Reporting Guidelines. The red dashed lines indicate the publication date of the MEDQUARG checklist.

The median (Q1–Q3) MEDQUARG score of 21 prevalence surveys assessed was 42.3% (30.8%–53.8%) (figure 3). Although 16 surveys were reported after the publication of the MEDQUARG in 2009, only 3 stated that the MEDQUARG guidelines were followed.^21 27 28^ Ten (47.6%) studies reported how the sample collectors presented to the seller (whether mystery or overt shopper and what the mystery shopper asked the seller) and 4 (19.0%) outlined the sampling design with sufficient details (online supplemental material 9). No studies provided stock size or turnover indices of the outlets sampled. Only 47.6% (10/21) of the studies provided definitions of the quality of medicines or recognised the WHO definition. In two (9.5%) surveys, the samples were clearly categorised as genuine, falsified or substandard or equivalent terminology (or the reason why this was not done was explained), and whether medicines were registered with the government in the location(s) sampled. No studies reported with sufficient details the relationship between packaging and chemistry results. The MRA of the sampled countries was either involved in the study (a representative of the MRA being an author in the paper) or was stated to be informed of its findings in 10 prevalence studies (47.6%).

10.1136/bmjgh-2021-006523.supp9 Supplementary data

### Equivalence studies

We found 116 equivalence studies from 1987 to 2020 with 161 data points and 822 samples tested, with a median (Q1–Q3) of 6 (4–9) samples per study, of 32 different API/API combinations collected in 51 countries (online supplemental materials 10 and 11). Of 822 samples, 30.5% (n=251) were SorF and 2.4% (n=20) were substandard, giving an overall FF of 33.0% in equivalence studies. Most samples were generics (72.3%, 594/822) with an FF of 31.6% (188/594) and innovator brands with an FF of 16.0% (4/25) (online supplemental material 11). No information on whether the samples were generics or innovators was reported for 203 samples. Out of 594 generic medicines, 71.9% of samples were collected in Asia (49.0%, 291/594) and Africa (22.9%, 136/594) with FFs of 27.8% (81/291) and 34.6% (47/136), respectively.

10.1136/bmjgh-2021-006523.supp10 Supplementary data

10.1136/bmjgh-2021-006523.supp11 Supplementary data

### Seizures, recalls and case reports

Seventy-seven publications describing recalls/warning/alerts (n=65), seizures (n=6) and case reports (n=6) of SF cardiovascular medicines in 34 countries were found. Twenty-seven reports described recalls of thousands of batches of sartan products containing N-nitrosodimethylamine (NDMA), N-nitrosodiethylamine (NDEA) and N-nitroso-N-methyl-4-aminobutyric acid (NMBA) impurities in 2018–2020 (online supplemental material 12). Recalls of products of 27 other APIs/combinations of APIs due to dissolution failure, API content or impurity/contaminant were also found (online supplemental material 13).

10.1136/bmjgh-2021-006523.supp12 Supplementary data

10.1136/bmjgh-2021-006523.supp13 Supplementary data

### Cardiovascular medical devices

After removal of duplicates, 5152 out of 6494 publications gathered through electronic and other sources searches were screened by title and abstract (online supplemental material 14). A total of 31 publications were included. We found no prevalence survey on the quality of cardiovascular medical devices. Nineteen recalls/alerts, 5 case reports and 2 seizures of SF cardiovascular device, published from 1994 to 2020, were found. The other five publications were general discussions about device quality.

10.1136/bmjgh-2021-006523.supp14 Supplementary data

Thirteen publications described recall/alert/case report of pacemakers and ICD issues due to hardware malfunctions, software errors or premature battery depletion (online supplemental material 15). Reports of more than 300 adverse reactions deemed associated with SF devices, including 181 deaths likely associated with devices failure, were identified.

10.1136/bmjgh-2021-006523.supp15 Supplementary data

Other publications included in our review are listed in online supplemental material 16 (medicines) and online supplemental material 17 (devices).

10.1136/bmjgh-2021-006523.supp16 Supplementary data

10.1136/bmjgh-2021-006523.supp17 Supplementary data

## Discussion

Overall, 822 (17.5%) of 4703 medicine samples collected in all studies included in this review failed at least one quality test. A similar FF (15.4%, 525/3414) was observed when considering only samples included in the 27 studies with the aim to assess the prevalence of SF medicines (‘prevalence surveys’). Most prevalence surveys were of limited quality with relatively subjective and diverse sampling methods and small sample sizes, making it very difficult to draw a clear picture of the global epidemiology of SF cardiovascular medicines and discuss associated factors. SF cardiovascular medicines were found in 24 countries (out of 28 where samples were collected) on four continents in prevalence surveys. Most failing samples contained out-of-specification impurity/contaminant levels and/or API content and/or dissolution defects. We found no prevalence surveys on the quality of cardiovascular medical devices, but we found recalls, seizures and case reports of SF devices in four continents, some associated with deadly consequences (181 people died because of device failure).

### Prevalence of SF medicines

The median number of samples (median of 30) collected per prevalence survey in this review is lower than that observed in a systematic review of antidiabetic medication quality (median of 112)^24^ and higher than that observed in a review of antimalarial quality (median of 10).^23^ Most samples were from LMICs, where more than three-quarters of CVD deaths occur.^2^ We found only one study conducted in China, despite a high incidence of CVDs, with an FF of 53.8% but with only 26 samples tested.^29^ In 2016, there were 245 million people in China with CVDs and 265.11–309.33 CVD-related deaths per 100 000 deaths per year.^30^ There were only 42 samples tested in three HICs, though past incidents suggest SF medicines are also present there. In one study, eight ‘commercial’ samples of clopidogrel–acetylsalicylic acid from Belgium failed either API content analysis or dissolution test, and one sample out of three clopidogrels obtained online dissolved poorly. Less than 100 samples were tested in three countries of the Americas (online supplemental material 5), with the highest observed FF. These data suggest that more investigations are needed globally, including in HICs, to assess whether, and the extent to how, SF may or not be involved in high rates of CVD and their complications.

We found no prevalence surveys for 25 of the 34 medicines used for CVDs included in the 2019 WHO EML,^7^ but we did find prevalence data on 14 API/combinations of APIs not included in the list. Over one-quarter of the samples of enalapril, clopidogrel–acetylsalicylic, nifedipine, candesartan, warfarin, lisinopril, simvastatin and captopril (four of them included in the WHO EML (enalapril, warfarin, lisinopril and simvastatin) failed at least one quality test, although with a number of samples tested per API lower than 50. Out of the 17 APIs with a Narrow Therapeutic Index (NTI), we found data on only three.^31^ Some tablets from three out of eight samples of warfarin tested were found outside normal API ranges of 95%–105% according to the USP, but all tablets were within the 85%–115% range. No sample among 165 samples of acenocoumarol and no samples (0/2) of digoxin failed. For NTI medicines such as warfarin, even API variation as small as 5% lower or higher than the specifications are very likely to lead to therapeutic failures or serious adverse reactions, sometimes irreversible. Monitoring the quality of NTI medicines is thus vital and has been recently advocated.^32^ We found no prevalence data on low-dose (less than 150 mg) acetylsalicylic acid, only limited evidence on clopidogrel’s quality, and no data on vital acute care medicines such as dopamine, streptokinase, heparin and glycoprotein IIb/IIIa inhibitors.

The most common defects were higher than acceptable levels of impurity/contaminant, incorrect API content and dissolution failure. For samples failing to contain the correct amount of API, whether the API was lower or higher than pharmacopoeial limits was often (82.6%, 355/430) not reported. When details were reported, a lower API than that stated was the most common defect. The administration of cardiovascular medicines containing lower API or with low dissolution rate risks low bioavailability. When taken mid-term or long term, they will likely lead to treatment failure with end-organ damage, and ultimately life-threatening complications and death.^33 34^ However, as far as we are aware, the consequences to patients exposed to subtherapeutic doses have not been studied. People may be exposed to SF for weeks if only one batch of a brand is affected, but they may receive good quality medicines at the next refill. Dose–response trials and pharmacokinetic and pharmacodynamic studies could provide an evidence base for understanding the consequences of medicines with API concentrations outside of pharmacopoeial specifications.^35^

‘Moderately’ high candesartan levels were detected in 12 samples (up to 112% API for pharmacopoeia specifications at 95%–105%); 1 sample of captopril contained 155% API; 1 sample of nifedipine contained 135% API; 1 warfarin sodium tablet and 1 epinephrine injection contained high API content, but no details on the exact amounts were given. Higher content than expected of these APIs poses high risks of adverse drug reactions.^36 37^ Potentially unsafe and unexpected APIs such as acetylsalicylic acid (in very low doses) or metronidazole in clopidogrel samples were also described.^38^

Various impurities, mainly API-related substances, such as nitrophenylpyridine and nitrosophenlylpyridine (in 75 samples),^39^ or ramipril–diketopiperazine (in 14 samples),^40^ were found at levels above specifications. Little is known about the safety of most impurities in pharmaceutical products. Some, for example, can pose toxic effects on DNA resulting in increased cancer risk.^41^ In a post hoc analysis using samples of the largest prevalence survey recently published, various impurities were found in captopril and amlodipine samples, but little is known about their clinical consequences.^42^ Investigation as to whether impurities/contaminants have carcinogenic effects or other significant effects and quantifying their risks are crucial toxicological problems requiring more research. Out-of-specifications levels of NDMA, NDEA and NMBA that are classified as probable human carcinogens were identified in thousands of batches of sartans worldwide in 2018–2019.^43 44^ The US FDA estimated that 1 additional case of cancer over a lifetime would be observed in 8000 patients using valsartan products containing NDMA impurity and 1 case in 18 000 patients using valsartan products containing NDEA at the highest valsartan dose daily for 4 years.^45^ However, in contrast, analysis of Danish nationwide registries of patients followed up for a median of 4.6 years between 2012 and 2017 showed no significant short-term increases in risk of cancer in 3400 patients exposed to NMDA impurities compared with 3625 patients unexposed to NMDA.^46^

### The quality of medical devices for CVD prevention and treatment

We found no prevalence surveys on cardiovascular device quality, although multiple issues related to pacemakers, ICDs, CRT-Ds or stents were identified. For example, hundreds of thousands of ICDs and CRT-Ds worldwide were recalled due to premature battery depletion, linked to adverse reactions and, in some cases, patient deaths.^22 47^ In a hospital in Lahore, Pakistan, and in a raid of a multinational company in Pakistan, allegedly fake stents were found in 2016–2017.^48 49^ Because of the nature of cardiovascular devices such as pacemakers, ICDs and stents, the assessment of the quality requires specific skills and resources, especially in settings with limited regulatory oversight.^50^ To better understand the epidemiology and impact of the problem, more research and regulatory inspections are needed. The functionality of regulatory systems for devices globally have been called into question with many reports of substandard products, including cardiovascular devices.^51^ New guidance by the WHO will be helpful for market surveillance of medical devices.^52^

### Limitations of the systematic review

Limitations of this review include that unpublished postmarketing surveillance results from MRAs and data from the pharmaceutical industry could not be captured. We found recalls/seizures/case reports mainly from a limited number of MRA’s websites and other websites interested in medicine quality, and Google, from 1994 to 2020. Our searches were done in English and French only.

### Quality of prevalence surveys reporting and methodology

Most prevalence surveys were conducted using convenience sampling, risking bias that could be reduced by use of randomised surveys, although at increased cost and time.^23^ The majority of the surveys were conducted in a limited number of geographical areas and with a small sample size of medicines. The quality of reporting of the prevalence studies was poor, as reflected by the low MEDQUARG scores. Only 3 out of 16 surveys published after the publication of the MEDQUARG guidelines in 2009 included MEDQUARG guidelines as part of the study protocol. Careful interpretation of the results and their generalisability is thus needed. The description of outlets where samples were collected was often ambiguous as articles rarely contained their details. Only two prevalence study papers stated whether medicines collected were substandard or falsified, although crucial to differentiate as their causes and proposed solutions differ. Because of these limitations, we did not perform statistical causal factor analysis such as the type of outlets or the country of the stated manufacturers. Indeed, although crucial to better inform policy, they could induce misleading results and interpretation.

Although it will be difficult to accurately define the prevalence of SF medicines, more research is needed to help improve the methods used for prevalence surveys of SF medicines in specific contexts.^53 54^ For example, generalisability of results could be improved by including knowledge of medicine use and consumption in the study areas in designing the methodology.

Systematic reviews and meta-analyses of SF medicine prevalence surveys will also benefit from a better understanding of confounding contextual variables such as social, political and environmental factors.^54^

## Conclusion

The evidence suggests that there are important issues with the quality of cardiovascular medicines/devices that will have important consequences for public health. Our results cannot be regarded as generalisable, and careful interpretation is needed; we do not state that 15.4% of cardiovascular medicines globally are SF. Surveys with standardised methods and reporting (ideally, using randomised sample collection and including the description of contextual aspects) will provide meaningful and generalisable estimates of the prevalence of the quality of cardiovascular medicines and how this changes through time and space. To better inform policy, more effort is needed to pinpoint problems and seek appropriate solutions. Ensuring quality cardiovascular medicines/devices from manufacturing throughout the supply chain to consumers, in the context of ever-rising CVDs, is crucial to public health.

## Data Availability

All data relevant to the study are included in the article or uploaded as supplementary information. All data relevant to the study are included in the article or uploaded as online supplementary information. The raw data will also be made available at https://www.iddo.org/mqsurveyor/%23cardiovascular after acceptance by the journal.

## References

[1] Inst. heal. metrics Eval. [Dataset]. Global burden of disease results tool | GHDx, 2020. Available: http://ghdx.healthdata.org/gbd-results-tool

[2] World Health Organization. About cardiovascular diseases. world heal. organ, 2011. Available: https://www.who.int/cardiovascular_diseases/about_cvd/en/

[3] World Health Organization. Cardiovascular diseases (CVDs). World heal. organ, 2020. Available: https://www.who.int/news-room/fact-sheets/detail/cardiovascular-diseases-(cvds)

[4] Gheorghe A, Griffiths U, Murphy A, et al. The economic burden of cardiovascular disease and hypertension in low- and middle-income countries: a systematic review. BMC Public Health 2018;18:975. 10.1186/s12889-018-5806-x 30081871 PMC6090747

[5] WHOCC. [Dataset]. WHOCC - ATC/DDD Index, 2019. Available: https://www.whocc.no/atc_ddd_index/?code=C

[6] Pharm Press. British National formulary (BNF) 77/ March 2019. London: Pharm Press, 2019.

[7] World Health Organization. World Health organization model list of essential medicines, 2019. Available: https://apps.who.int/iris/bitstream/handle/10665/325771/WHO-MVP-EMP-IAU-2019.06-eng.pdf?ua=1

[8] United States Food and Drug Administration. CFR - code of federal regulations title 21-Food and drug administration department of health and human services subchapter - medical devices part 870 cardiovascular devices, 2019. Available: https://www.accessdata.fda.gov/scripts/cdrh/cfdocs/cfcfr/CFRSearch.cfm?CFRPart=870

[9] World Health Organization. The world medicines situation report, 2011. 3rd edition. Geneva: World Health Organization, 2011. https://www.who.int/medicines/areas/policy/world_medicines_situation/en/

[10] Pharmaceutical Technology. Who were the top players in cardiovascular disease in 2017? Pharm. Technol, 2019. Available: https://www.pharmaceutical-technology.com/comment/top-players-cardiovascular-disease-2017/

[11] Market research engine. Cardiovascular drugs market size, forecast 2018-2024. mark. Res. engine, 2019. Available: https://www.marketresearchengine.com/cardiovascular-drugs-market

[12] Intrado GlobeNewswire. Cardiovascular devices market to reach USD 69.08 billion by 2026. Intrado GlobeNewswire, 2019. Available: https://www.globenewswire.com/news-release/2019/05/22/1841035/0/en/Cardiovascular-Devices-Market-to-Reach-USD-69-08-Billion-by-2026-Reports-And-Data.html

[13] World Health Organization. Member state mechanism on substandard/spurious/falsely-labelled/falsified/ counterfeit medical products, 2017. Available: https://apps.who.int/medicinedocs/documents/s23501en/s23501en.pdf

[14] Nayyar GML, Breman JG, Herrington JE. The global pandemic of falsified medicines: laboratory and field innovations and policy perspectives. Am J Trop Med Hyg 2015;92:2–7. 10.4269/ajtmh.15-0221 25897072 PMC4455081

[15] Newton PN, Bond KC, Oxford Statement signatories. Global access to quality-assured medical products: the Oxford statement and call to action. Lancet Glob Health 2019;7:e1609–11. 10.1016/S2214-109X(19)30426-7 31708137

[16] World Health Organization. A study on the public health and socioeconomic impact of substandard and falsified medical products, 2017. Available: https://www.who.int/medicines/regulation/ssffc/publications/se-study-sf/en/

[17] Almuzaini T, Sammons H, Choonara I. Substandard and falsified medicines in the UK: a retrospective review of drug alerts (2001-2011). BMJ Open 2013;3:e002924. 10.1136/bmjopen-2013-002924 PMC3731779 23883882

[18] Rosania L. Heparin crisis 2008: a tipping point for increased FDA enforcement in the pharma sector. HeinOnline, 2019. Available: https://heinonline.org/hol-cgi-bin/get_pdf.cgi?handle=hein.journals/foodlj65&section=27 24479237

[19] Tribunal JI. Batch J093. The pathology of negligence. Report of the Judicial inquiry Tribunal to determine the causes of deaths of patients of the Punjab Institute of cardiology, Lahore in 2011-2012, 2012. Available: https://padproject.nd.edu/assets/206307/pathology_of_negligence_pic_drug_inquiry_report_2012.pdf

[20] World Health Organization. Global surveillance and monitoring system for substandard and falsified medical products, 2017. Available: https://apps.who.int/iris/bitstream/handle/10665/326708/9789241513425-eng.pdf?ua=1

[21] Antignac M, Diop BI, Do B, et al. Quality assessment of 7 cardiovascular drugs in 10 sub-Saharan countries: the seven study. JAMA Cardiol 2017;2:223–5. 10.1001/jamacardio.2016.3851 27760238

[22] Turner T. St. Jude medical implantable defibrillators. Drugwatch, 2019. Available: https://www.drugwatch.com/defibrillators/

[23] Tabernero P, Fernández FM, Green M, et al. Mind the gaps--the epidemiology of poor-quality anti-malarials in the malarious world--analysis of the WorldWide Antimalarial Resistance Network database. Malar J 2014;13:139. 10.1186/1475-2875-13-139 24712972 PMC4021408

[24] Saraswati K, Sichanh C, Newton PN, et al. Quality of medical products for diabetes management: a systematic review. BMJ Glob Health 2019;4:e001636. 10.1136/bmjgh-2019-001636 PMC6768360 31637025

[25] Newton PN, Lee SJ, Goodman C, et al. Guidelines for field surveys of the quality of medicines: a proposal. PLoS Med 2009;6:e1000052. 10.1371/journal.pmed.1000052 PMC2659710 19320538

[26] The World Bank. [Dataset]. World Bank country and lending groups, 2020. Available: https://datahelpdesk.worldbank.org/knowledgebase/articles/906519-world-bank-country-and-lending-groups

[27] Schäfermann S, Wemakor E, Hauk C, et al. Quality of medicines in southern Togo: investigation of antibiotics and of medicines for non-communicable diseases from pharmacies and informal vendors. PLoS One 2018;13:e0207911. 10.1371/journal.pone.0207911 30496234 PMC6264819

[28] Rahman MS, Yoshida N, Tsuboi H, et al. A cross-sectional investigation of the quality of selected medicines for noncommunicable diseases in private community drug outlets in Cambodia during 2011-2013. Am J Trop Med Hyg 2019;101:1018–26. 10.4269/ajtmh.19-0247 31516106 PMC6838583

[29] Kakio T, Nagase H, Takaoka T, et al. Survey to identify substandard and falsified tablets in several Asian countries with pharmacopeial quality control tests and principal component analysis of handheld Raman spectroscopy. Am J Trop Med Hyg 2018;98:1643–52. 10.4269/ajtmh.17-0553 29611498 PMC6086158

[30] Ma L-Y, Chen W-W, Gao R-L, et al. China cardiovascular diseases report 2018: an updated summary. J Geriatr Cardiol 2020;17:1–8. 10.11909/j.issn.1671-5411.2020.01.001 32133031 PMC7008101

[31] DrugBank. [Dataset]. Narrow therapeutic index drugs, 2019. Available: https://www.drugbank.ca/categories/DBCAT003972

[32] Antignac M, Bara Diop I, Macquart de Terline D, et al. Falsified and substandard cardiovascular drugs in Africa: a need for continued monitoring strategies. J Glob Health 2019;9:010302. 10.7189/jogh.09.020302 PMC6815872 31673334

[33] Rothwell PM, Howard SC, Dolan E, et al. Effects of beta blockers and calcium-channel blockers on within-individual variability in blood pressure and risk of stroke. Lancet Neurol 2010;9:469–80. 10.1016/S1474-4422(10)70066-1 20227347

[34] Nobili A, Pasina L, Latini R. Beta-Adrenoceptor antagonists and antianginal drugs. Side Eff Drugs Annu 2014;35:351–7.

[35] Ponto LL, Schoenwald RD, Furosemide SRD. Furosemide (frusemide). A pharmacokinetic/pharmacodynamic review (Part I). Clin Pharmacokinet 1990;18:381–408. 10.2165/00003088-199018050-00004 2185908

[36] Pedrós C, Formiga F, Corbella X, et al. Adverse drug reactions leading to urgent hospital admission in an elderly population: prevalence and main features. Eur J Clin Pharmacol 2016;72:219–26. 10.1007/s00228-015-1974-0 26546335

[37] Pardo Cabello AJ, Del Pozo Gavilán E, Gómez Jiménez FJ, et al. Drug-related mortality among inpatients: a retrospective observational study. Eur J Clin Pharmacol 2016;72:731–6. 10.1007/s00228-016-2026-0 26896941

[38] ElTantawy ME, Bebawy LI, Shokry RF. Chromatographic determination of clopidogrel bisulfate; detection and quantification of counterfeit Plavix® tablets. Bulletin of Faculty of Pharmacy, Cairo University 2014;52:91–101. 10.1016/j.bfopcu.2014.04.003

[39] Ndichu ET, Ohiri K, Sekoni O, et al. Evaluating the quality of antihypertensive drugs in Lagos state, Nigeria. PLoS One 2019;14:e0211567. 10.1371/journal.pone.0211567 30759124 PMC6373917

[40] Angeli DG, Trezza C. Quality and stability of ramipril generics/copies versus reference ramipril (Tritace): a 3-month stability comparative study. Clin Drug Investig 2009;29:667–76. 10.2165/11315270-000000000-00000 19715383

[41] Cok S, Emerce E. Overview of impurities in pharmaceuticals: toxicological aspects. Asian Chem Lett, 2019. Available: https://www.researchgate.net/publication/259150621_overview_of_impurities_in_pharmaceuticals_toxicological_aspects

[42] Secretan P-H, Antignac M, Yagoubi N, et al. Post hoc study to investigate the potential causes of poor quality of cardiovascular medicines collected in sub-Saharan countries. BMJ Open 2020;10:e039252. 10.1136/bmjopen-2020-039252 PMC7654128 33168557

[43] Verna L, Whysner J, Williams GM. N-Nitrosodiethylamine mechanistic data and risk assessment: bioactivation, DNA-adduct formation, mutagenicity, and tumor initiation. Pharmacol Ther 1996;71:57–81. 10.1016/0163-7258(96)00062-9 8910949

[44] World Health Organization. Nitrosodimethylamine (NDMA), 2008. Available: https://www.who.int/water_sanitation_health/dwq/chemicals/ndmasummary_2ndadd.pdf

[45] United States Food and Drug Administration. Laboratory analysis of valsartan products, 2019. Available: https://www.fda.gov/drugs/drug-safety-and-availability/laboratory-analysis-valsartan-products

[46] Pottegård A, Kristensen KB, Ernst MT, Hallas J, Pottegard A, et al. Use of N-nitrosodimethylamine (NDMA) contaminated valsartan products and risk of cancer: Danish nationwide cohort study. BMJ 2018;362:k3851. 10.1136/bmj.k3851 30209057 PMC6134800

[47] United States Food and Drug Administration. Update - St. Jude medical recalls implantable cardioverter defibrillators (ICD) and cardiac resynchronization therapy defibrillators (CRT-D) due to premature battery depletion. USFDA, 2017. Available: https://wayback.archive-it.org/7993/20201222125204/https://www.fda.gov/medical-devices/medical-device-recalls/update-st-jude-medical-recalls-implantable-cardioverter-defibrillators-icd-and-cardiac

[48] Pakistan Today. DRA recovers 40 substandard cardiac stents, seals company’s office, 2017. Available: https://www.pakistantoday.com.pk/2017/01/22/dra-recovers-40-substandard-cardiac-stents-seals-companys-office/

[49] Hakim B. FIA exposes mafia selling fake stents at over 3200% markup, 2016. Available: https://propakistani.pk/2017/01/18/fia-exposes-mafia-selling-fake-stents-3200-markup/

[50] Mori M, Ravinetto R, Jacobs J. Quality of medical devices and in vitro diagnostics in resource-limited settings. Trop Med Int Health 2011;16:1439–49. 10.1111/j.1365-3156.2011.02852.x 21955331

[51] Godlee F. Why aren’t medical devices regulated like drugs? Br Med J 2018;363:k5032

[52] World Health Organization. Guidance for post-market surveillance and market surveillance of medical devices, including in vitro diagnostics, 2020. Available: https://www.who.int/publications/i/item/9789240015319

[53] Mackey TK. Prevalence of substandard and Falsified essential medicines: still an incomplete picture. JAMA Netw Open 2018;1:e181685. 10.1001/jamanetworkopen.2018.1685 30646099

[54] McManus D, Naughton BD. A systematic review of substandard, falsified, unlicensed and unregistered medicine sampling studies: a focus on context, prevalence, and quality. BMJ Glob Health 2020;5:e002393. 10.1136/bmjgh-2020-002393 PMC7454198 32859648

