# Supplementary material for: The quality of medical products for cardiovascular diseases: a gap in global cardiac care
Source: BMJ Glob Health. 2021 Sep 14;6(9):e006523. doi: 10.1136/bmjgh-2021-006523 (PMC8442059; doi:10.1136/bmjgh-2021-006523)
Supplement: Supplementary data [file bmjgh-2021-006523supp001.pdf]

Supplementary file 1: Search terms used for each source in the systematic review of the quality of cardiovascular medicines/ devices A. medicines and B. devices	
**A. Medicines**	
**Sources**	**English search Terms- Medicines**
Pubmed	((substandard OR sub-standard OR falsified OR counterfeit OR fake OR spurious OR “drug quality”OR “medicine quality” OR “pharmaceutical quality” OR degraded OR contaminated OR adulterated OR impurity) AND (“cardiovascular medicine” OR “cardiovascular drug” OR “cardiac drug” OR “cardiac medicine” OR “beta blocking” OR “beta-blocker” OR “alpha-blocking” OR "alpha-adrenoreceptor antagonists" OR “calcium channel blocker” OR “renin-angiotensin” OR “ACE INHIBITOR” OR Antiarrhythmic OR “Anti-arrhythmic” OR antihypertensive OR “anti-hypertensive” OR diuretic OR thiazide OR “lipid modifying” OR antihyperlipidemic OR hypolipidemic OR “HMG CoA reductase inhibitor” OR statin OR Fibrate OR “Bile acid sequestrant” OR anticoagulant OR antiplatelet OR antithrombotic OR hypertension OR “heart failure” OR “lipid regulating” OR digoxin OR strophanthin OR ouabain OR oxprenolol OR pindolol OR propranolol OR timolol OR sotalol OR nadolol OR atenolol OR bisoprolol OR celiprolol OR esmolol OR nebivolol OR metoprolol OR labetalol OR carvedilol OR amlodipine OR felodipine OR isradipine OR nicardipine OR nifedipine OR nimodipine OR lacidipine OR lercanidipine OR verapamil OR diltiazem OR captopril OR enalapril OR lisinopril OR perindopril OR ramipril OR quinapril OR cilazapril OR fosinopril OR trandolapril OR moexipril OR imidapril OR losartan OR eprosartan OR valsartan OR irbesartan OR candesartan OR telmisartan OR olmesartan OR azilsartan OR aliskiren OR quinidine OR disopyramide OR hydroquinidine OR lidocaine OR propafenone OR flecainide OR amiodarone OR dronedarone OR norepinephrine OR noradrenaline OR dopamine OR phenylephrine OR dobutamine OR metaraminol OR dopexamine OR epinephrine OR adrenaline OR ephedrine OR milrinone OR enoximone OR nicorandil OR adenosine OR ivabradine OR ranolazine OR Methyldopa OR clonidine OR guanfacine OR moxonidine OR prazosin OR indoramin OR doxazosin OR guanethidine OR diazoxide OR hydralazine OR minoxidil OR nitroprusside OR bosentan OR ambrisentan OR hydrochlorothiazide OR bendroflumethiazide OR cyclopenthiazide OR chlortalidone OR metolazone OR xipamide OR indapamide OR furosemide OR frusemide OR bumetanide OR torasemide OR spironolactone OR eplerenone OR amiloride OR triamterene OR “inositol nicotinate” OR “hexa niacinate” OR hexanicotinate OR pentoxifylline OR phenoxybenzamine OR moxisylyte OR naftidrofuryl OR “glyceryl trinitrate” OR Nitroglycerin OR “isosorbide dinitrate” OR “isosorbide mononitrate” OR simvastatin OR pravastatin OR fluvastatin OR atorvastatin OR rosuvastatin OR bezafibrate OR gemfibrozil OR fenofibrate OR ciprofibrate OR cholestyramine OR colestipol OR colesevelam OR ezetimibe OR “nicotinic acid” OR acipimox OR heparin OR argatroban OR dalteparin OR tinzaparin OR danaparoid OR phenindione OR warfarin OR acenocoumarol OR clopidogrel OR aspirin OR “acetylsalicylic acid” OR dipyridamole OR epoprostenol OR iloprost OR eptifibatide OR tirofiban OR prasugrel OR cilostazol OR ticagrelor OR bivalirudin OR dabigatran OR rivaroxaban OR apixaban OR fondaparinux OR Streptokinase OR alteplase OR urokinase OR reteplase OR tenecteplase)) NOT (meat OR milk OR water OR vegetable OR soil OR wastewater OR environment OR cellular)
	(((substandard or falsified or counterfeit or fake or spurious or "drug quality" or "medicine quality" or "pharmaceutical quality" or degraded or contaminated or adulterated or impurity) and ("cardiovascular medicine" or "cardiovascular drug" or "cardiac drug" or "cardiac medicine" or "beta blocker" or "alpha blocker" or "calcium channel blocker" or "renin angiotensin" or "ACE INHIBITOR" or Antiarrhythmic or antihypertensive or diuretic or thiazide or antihyperlipidemic or hypolipidemic or statin or Fibrate or anticoagulant or antiplatelet or antithrombotic or digoxin or strophanthin or ouabain or oxprenolol or pindolol or propranolol or

Embase	timolol or sotalol or nadolol or atenolol or bisoprolol or celiprolol or esmolol or nebivolol or metoprolol or labetalol or carvedilol or amlodipine or felodipine or isradipine or nicardipine or nifedipine or nimodipine or lacidipine or lercanidipine or verapamil or diltiazem or captopril or enalapril or lisinopril or perindopril or ramipril or quinapril or cilazapril or fosinopril or trandolapril or moexipril or imidapril or losartan or eprosartan or valsartan or irbesartan or candesartan or telmisartan or olmesartan or azilsartan or aliskiren or quinidine or disopyramide or hydroquinidine or lidocaine or propafenone or flecainide or amiodarone or dronedarone or norepinephrine or noradrenaline or dopamine or phenylephrine or dobutamine or metaraminol or dopexamine or epinephrine or adrenaline or ephedrine or milrinone or enoximone or nicorandil or adenosine or ivabradine or ranolazine or Methyldopa or clonidine or guanfacine or moxonidine or prazosin or indoramin or doxazosin or guanethidine or diazoxide or hydralazine or minoxidil or nitroprusside or bosentan or ambrisentan or hydrochlorothiazide or bendroflumethiazide or cyclopenthiazide or metolazone or xipamide or indapamide or furosemide or frusemide or bumetanide or torasemide or spironolactone or eplerenone or amiloride or triamterene or "inositol nicotinate" or phenoxybenzamine or naftidrofuryl or "glyceryl trinitrate" or Nitroglycerin or "isosorbide dinitrate" or "isosorbide mononitrate" or simvastatin or pravastatin or fluvastatin or atorvastatin or rosuvastatin or bezafibrate or gemfibrozil or fenofibrate or ciprofibrate or cholestyramine or colestipol or colesevelam or ezetimibe or "nicotinic acid" or acipimox or ezetimibe or heparin or argatroban or dalteparin or tinzaparin or danaparoid or phenindione or warfarin or acenocoumarol or clopidogrel or aspirin or "acetylsalicylic acid" or dipyridamole or epoprostenol or iloprost or eptifibatide or tirofiban or prasugrel or cilostazol or ticagrelor or bivalirudin or dabigatran or rivaroxaban or apixaban or fondaparinux or Streptokinase or alteplase or urokinase or reteplase or tenecteplase)) not (meat or milk or water or vegetable or soil or wastewater or environment or cellular))
Web of Science	(((substandard OR falsified OR counterfeit OR fake OR spurious OR “drug quality” OR “medicine quality” OR “pharmaceutical quality” OR degraded OR contaminated OR adulterated OR impurity) AND (“cardiovascular medicine” OR “cardiovascular drug” OR “cardiac drug” OR “cardiac medicine” OR “beta-blocker” OR “alpha-blocker” OR “calcium channel blocker” OR “renin-angiotensin” OR “ACE INHIBITOR” OR Antiarrhythmic OR antihypertensive OR diuretic OR thiazide OR antihyperlipidemic OR hypolipidemic OR statin OR Fibrate OR “Bile acid sequestrant” OR anticoagulant OR antiplatelet OR antithrombotic OR digoxin OR strophanthin OR ouabain OR oxprenolol OR pindolol OR propranolol OR timolol OR sotalol OR nadolol OR atenolol OR bisoprolol OR celiprolol OR esmolol OR nebivolol OR metoprolol OR labetalol OR carvedilol OR amlodipine OR felodipine OR isradipine OR nicardipine OR nifedipine OR nimodipine OR lacidipine OR lercanidipine OR verapamil OR diltiazem OR captopril OR enalapril OR lisinopril OR perindopril OR ramipril OR quinapril OR cilazapril OR fosinopril OR trandolapril OR moexipril OR imidapril OR losartan OR eprosartan OR valsartan OR irbesartan OR candesartan OR telmisartan OR olmesartan OR azilsartan OR aliskiren OR quinidine OR disopyramide OR hydroquinidine OR lidocaine OR propafenone OR flecainide OR amiodarone OR dronedarone OR norepinephrine OR noradrenaline OR dopamine OR phenylephrine OR dobutamine OR metaraminol OR dopexamine OR epinephrine OR adrenaline OR ephedrine OR milrinone OR enoximone OR nicorandil OR adenosine OR ivabradine OR ranolazine OR Methyldopa OR clonidine OR guanfacine OR moxonidine OR prazosin OR indoramin OR doxazosin OR guanethidine OR diazoxide OR hydralazine OR minoxidil OR nitroprusside OR bosentan OR ambrisentan OR hydrochlorothiazide OR bendroflumethiazide OR cyclopenthiazide OR metolazone OR xipamide OR indapamide OR furosemide OR frusemide OR bumetanide OR torasemide OR spironolactone OR eplerenone OR amiloride OR triamterene OR “inositol nicotinate" OR pentoxifylline OR phenoxybenzamine OR naftidrofuryl OR “glyceryl trinitrate” OR Nitroglycerin OR “isosorbide dinitrate” OR “isosorbide mononitrate” OR simvastatin OR pravastatin OR fluvastatin OR atorvastatin OR rosuvastatin OR bezafibrate OR gemfibrozil OR fenofibrate OR ciprofibrate OR cholestyramine OR colestipol OR colesevelam OR ezetimibe OR “nicotinic acid” OR acipimox OR ezetimibe OR heparin OR argatroban OR dalteparin OR tinzaparin OR danaparoid OR phenindione OR warfarin

	OR acenocoumarol OR clopidogrel OR aspirin OR "acetylsalicylic acid" OR dipyridamole OR epoprostenol OR iloprost OR eptifibatide OR tirofiban OR prasugrel OR cilostazol OR ticagrelor OR bivalirudin OR dabigatran OR rivaroxaban OR apixaban OR fondaparinux OR Streptokinase OR alteplase OR urokinase OR reteplase OR tenecteplase)) NOT (meat OR milk OR water OR vegetable OR soil OR wastewater OR environment OR cellular))
Google Scholar	("drug quality" OR "medicine quality" OR degraded OR adulterated OR substandard OR falsified OR counterfeit OR fake OR spurious OR contaminated OR impurity) AND ("cardiovascular medicine" OR "cardiovascular drug")
	("drug quality" OR "medicine quality" OR degraded OR adulterated OR substandard OR falsified OR counterfeit OR fake OR spurious OR contaminated OR impurity) AND ("cardiac drug" OR "cardiac medicine" OR "beta-blocker")
	("drug quality" OR "medicine quality" OR degraded OR adulterated OR substandard OR falsified OR counterfeit OR fake OR spurious OR contaminated OR impurity) AND ("alpha-blocker" OR "calcium channel blocker" OR "angiotensin* blocker")
	("drug quality" OR "medicine quality" OR degraded OR adulterated OR substandard OR falsified OR counterfeit OR fake OR spurious OR contaminated OR impurity) AND ("ACE INHIBITOR" OR Antiarrhythmic OR antihypertensive OR diuretic)
	("drug quality" OR "medicine quality" OR degraded OR adulterated OR substandard OR falsified OR counterfeit OR fake OR spurious OR contaminated OR impurity) AND (thiazide* OR "aldosterone antagonists" OR antihyperlipidemic)
	("drug quality" OR "medicine quality" OR degraded OR adulterated OR substandard OR falsified OR counterfeit OR fake OR spurious OR contaminated OR impurity) AND (anticoagulant OR antiplatelet OR antithrombotic OR hypolipidemic)
	("drug quality" OR "medicine quality" OR degraded OR adulterated OR substandard OR falsified OR counterfeit OR fake OR spurious OR contaminated OR impurity) AND (digoxin OR propranolol OR atenolol OR bisoprolol OR amlodipine OR nifedipine)
	("drug quality" OR "medicine quality" OR degraded OR adulterated OR substandard OR falsified OR counterfeit OR fake OR spurious OR contaminated OR impurity) AND (epinephrine OR ephedrine OR hydralazine OR hydrochlorothiazide OR furosemide)
	("drug quality" OR "medicine quality" OR degraded OR adulterated OR substandard OR falsified OR counterfeit OR fake OR spurious OR contaminated OR impurity) AND (spironolactone OR amiloride OR "glyceryl trinitrate" OR Nitroglycerin)
	("drug quality" OR "medicine quality" OR degraded OR adulterated OR substandard OR falsified OR counterfeit OR fake OR spurious OR contaminated OR impurity) AND ("isosorbide dinitrate" OR simvastatin OR heparin OR warfarin OR clopidogrel)
	("drug quality" OR "medicine quality" OR degraded OR adulterated OR substandard OR falsified OR counterfeit OR fake OR spurious OR contaminated OR impurity) AND (statin OR fibrate OR aspirin OR "acetylsalicylic acid"OR Streptokinase)
	("drug quality" OR "medicine quality" OR degraded OR adulterated OR substandard OR falsified OR counterfeit OR fake OR spurious OR contaminated OR impurity) AND (timolol OR sotalol OR metoprolol OR labetalol OR captopril OR valsartan OR irbesartan)
	("drug quality" OR "medicine quality" OR degraded OR adulterated OR substandard OR falsified OR counterfeit OR fake OR spurious OR contaminated OR impurity) AND (candesartan OR telmisartan OR milrinone OR Methyldopa OR clonidine OR doxazosin)
	("drug quality" OR "medicine quality" OR degraded OR adulterated OR substandard OR falsified OR counterfeit OR fake OR spurious OR contaminated OR impurity) AND (atorvastatin OR rosuvastatin OR fenofibrate OR ticagrelor OR nicardipine OR rivaroxaban)
	("drug quality" OR "medicine quality" OR degraded OR adulterated OR substandard OR falsified OR counterfeit OR fake OR spurious OR "falsely labelled" OR contaminated OR impurity) AND ("cardiovascular* medicine" OR "cardiovascular drug" OR

Google	“cardiac* drug” OR “cardiac medicine” OR "beta-blocker" OR “alpha-blocker” OR “calcium channel blocker” OR “angiotensin blocker”)
	(“drug quality” OR “medicine quality” OR degraded OR adulterated OR substandard OR falsified OR counterfeit OR fake OR spurious OR contaminated OR impurity) AND (“ACE INHIBITOR” OR Antiarrhythmic OR antihypertensive OR diuretic OR thiazide* OR "aldosterone antagonists" OR antihyperlipidemic OR anticoagulant OR antiplatelet OR antithrombotic OR hypolipidemic OR digoxin OR propranolol OR atenolol OR bisoprolol OR amlodipine OR nifedipin OR propafenone OR dobutamine OR adrenaline)
	(“drug quality” OR “medicine quality” OR degraded OR adulterated OR substandard OR falsified OR counterfeit OR fake OR spurious OR contaminated OR impurity) AND (epinephrine OR ephedrine OR hydralazine OR hydrochlorothiazide OR furosemide OR spironolactone OR amiloride OR “glyceryl trinitrate” OR Nitroglycerin OR “isosorbide dinitrate” OR simvastatin OR heparin OR warfarin OR clopidogrel OR statin OR fibrate OR Streptokinase)
	(“drug quality” OR “medicine quality” OR degraded OR adulterated OR substandard OR falsified OR counterfeit OR fake OR spurious OR contaminated OR impurity) AND (timolol OR sotalol OR metoprolol OR labetalol OR captopril OR valsartan OR irbesartan OR candesartan OR telmisartan OR milrinone OR Methyldopa OR clonidine OR doxazosin OR atorvastatin OR rosuvastatin OR fenofibrate OR ticagrelor OR nicardipine OR rivaroxaban)
	(“drug quality” OR “medicine quality” OR degraded OR adulterated OR substandard OR falsified OR counterfeit OR fake OR spurious OR contaminated OR impurity) AND (pindolol OR nadolol OR celiprolol OR esmolol OR carvedilol OR felodipine OR nimodipine OR verapamil OR diltiazem OR lisinopril OR perindopril OR ramipril OR quinapril OR fosinopril OR trandolapril OR moexipril OR nitroprusside)
	(“drug quality” OR “medicine quality” OR degraded OR adulterated OR substandard OR falsified OR counterfeit OR fake OR spurious OR contaminated OR impurity) AND (aspirin OR “acetylsalicylic acid” OR phenoxybenzamine OR moxisylyte OR naftidrofuryl ” OR “isosorbide mononitrate” OR pravastatin OR fluvastatin OR bezafibrate OR gemfibrozil OR ciprofibrate OR cholestyramine OR colestipol OR colesevelam OR ezetimibe OR dabigatran)
	(“drug quality” OR “medicine quality” OR degraded OR adulterated OR substandard OR falsified OR counterfeit OR fake OR spurious OR contaminated OR impurity) AND (“nicotinic acid” OR acipimox OR ezetimibe OR argatroban OR dalteparin OR tinzaparin OR danaparoid OR phenindione OR acenocoumarol OR dipyridamole OR epoprostenol OR iloprost OR eptifibatide OR tirofiban OR prasugrel OR cilostazol OR bivalirudin OR apixaban)
	(“drug quality” OR “medicine quality” OR degraded OR adulterated OR substandard OR falsified OR counterfeit OR fake OR spurious OR contaminated OR impurity) AND (bosentan OR ambrisentan OR chlortalidone OR metolazone OR xipamide OR indapamide OR bumetanide OR torasemide OR eplerenone OR triamterene OR “inositol nicotinate” OR “hexa niacinate” OR hexanicotinate OR pentoxifylline OR fondaparinux OR reteplase OR tenecteplase)
**Sources**	**French search Terms- Medicines**
	(contrefaçon OR sous-standard OR faux OR falsifié OR "qualité des médicaments" OR adultéré OR contaminé OR impureté) AND ("médicament cardiovasculaire" OR bêta-bloquant OR "inhibiteur des canaux calciques" OR "Inhibiteurs de l'ECA")
	(contrefaçon OR sous-standard OR faux OR falsifié OR "qualité des médicaments" OR adultéré OR contaminé OR impureté) AND (Antiarythmique OR Antihypertenseur OR thiazidique OR antihyperlipidémique OR anticoagulant OR antiplaquettaire OR antithrombotique OR hypolipémiant OR digoxine OR propranolol OR aténolol)

BMJ Publishing Group Limited (BMJ) disclaims all liability and responsibility arising from any reliance placed on this supplemental material which has been supplied by the author(s)

<table>
<tr><td rowspan="12">Google Scholar</td><td>(contrefaçon OR sous-standard OR faux OR falsifié OR “qualité des médicaments” OR adultéré OR contaminé OR impureté) AND (épinéphrine OR éphédrine OR hydralazine OR isoprolol OR amlodipine OR nifédipine OR propafénone OR dobutamine)</td></tr>
<tr><td>(contrefaçon OR sous-standard OR faux OR falsifié OR “qualité des médicaments” OR adultéré OR contaminé OR impureté) AND (hydrochlorothiazide OR furosémide OR spironolactone OR amiloride OR "trinitrate de glycéryle" OR Nitroglycérine)</td></tr>
<tr><td>(contrefaçon OR sous-standard OR faux OR falsifié OR “qualité des médicaments” OR adultéré OR contaminé OR impureté) AND ("dinitrate d'isosorbide" OR simvastatine OR héparine OR warfarine OR clopidogrel OR statine OR fibrate OR streptokinase)</td></tr>
<tr><td>(contrefaçon OR sous-standard OR faux OR falsifié OR “qualité des médicaments” OR adultéré OR contaminé OR impureté) AND (timolol OR sotalol OR métoprolol OR labétalol OR captopril OR valsartan OR irsartan OR irbesartan OR candesartan)</td></tr>
<tr><td>(contrefaçon OR sous-standard OR faux OR falsifié OR “qualité des médicaments” OR adultéré OR contaminé OR impureté) AND (telmisartan OR Méthyldopa OR clonidine OR doxazosine OR atorvastatine OR rosuvastatine OR fénofibrate OR ticagrélor)</td></tr>
<tr><td>(contrefaçon OR sous-standard OR faux OR falsifié OR “qualité des médicaments” OR adultéré OR contaminé OR impureté) AND (carvédolol OR félodipine OR nimodipine OR Vérapamil OR diltiazem OR périndopril OR ramipril OR quinapril OR fosinopril OR trandolapril OR moexipril OR nitroprussiate)</td></tr>
<tr><td>(contrefaçon OR sous-standard OR faux OR falsifié OR “qualité des médicaments” OR adultéré OR contaminé OR impureté) AND (phénoxybenzamine OR moxisylyte OR naftidrofuryl OR "isosorbide mononitrate" OR pravastatine OR Fluvastatine)</td></tr>
<tr><td>(contrefaçon OR sous-standard OR faux OR falsifié OR “qualité des médicaments” OR adultéré OR contaminé OR impureté) AND (Bézafibrate OR gemfibrozil OR ciprofibrate OR cholestyramine OR colestipol OR colesevelam OR ézétimibe OR dabigatran)</td></tr>
<tr><td>(contrefaçon OR sous-standard OR faux OR falsifié OR “qualité des médicaments” OR adultéré OR contaminé OR impureté) AND ("acide nicotinique" OR acipimox OR ézétimibe OR argatroban OR daltéparine OR tinzaparine OR alteplase OR urokinase)</td></tr>
<tr><td>(contrefaçon OR sous-standard OR faux OR falsifié OR “qualité des médicaments” OR adultéré OR contaminé OR impureté) AND (Phénindione OR Fluindione OR acénocoumarol OR dipyridamole OR époprosténol OR iloprost OR eptifibatide OR tirofiban)</td></tr>
<tr><td>(contrefaçon OR sous-standard OR faux OR falsifié OR “qualité des médicaments” OR adultéré OR contaminé OR impureté) AND (bosentan OR ambrisentan OR chlortalidone OR métolazone OR xipamide OR indapamide OR prasugrel OR cilostazol OR bivalirudine OR apixaban)</td></tr>
<tr><td>(contrefaçon OR sous-standard OR faux OR falsifié OR “qualité des médicaments” OR adultéré OR contaminé OR impureté) AND (bumétanide OR torasémide OR éplerénone OR "nicotinate d'inositol"OR fondaparinux OR retéplase OR Ténectéplase)</td></tr>
<tr><td rowspan="4">Google</td><td>(contrefaçon | sous-standard | faux | falsifié | "qualité des médicaments" | adultéré | contaminé | impureté) AND ("médicament cardiovasculaire" | bêta-bloquant | "inhibiteur des canaux calciques" | "Inhibiteurs de l'ECA" | Antiarythmique | Antihypertenseur | thiazidique | antihyperlipidémique | anticoagulant | antiplaquettaire | antithrombotique | hypolipémiant)</td></tr>
<tr><td>(contrefaçon | sous-standard | faux | falsifié | "qualité des médicaments" | adultéré | contaminé | impureté) AND (digoxine | propranolol | aténolol | bisoprolol | amlodipine | nifédipine | propafénone | dobutamine | adrénaline | aspirine | "acide acétylsalicylique" | "inhibiteur de l'angiotensine" | "alpha-bloquant")</td></tr>
<tr><td>(contrefaçon | sous-standard | faux | falsifié | “qualité des médicaments” | adultéré | contaminé | impureté) AND (épinéphrine | éphédrine | hydralazine | hydrochlorothiazide | furosémide | spironolactone | amiloride | "trinitrate de glycéryle" | Nitroglycérine | "dinitrate d'isosorbide" | simvastatine | héparine | warfarine | clopidogrel | statine | fibrate | streptokinase)</td></tr>
<tr><td>(contrefaçon | sous-standard | faux | falsifié | “qualité des médicaments” | adultéré | contaminé | impureté) AND (timolol | sotalol | métoprolol | labétalol | captopril | valsartan | irsartan | irbesartan | candesartan | telmisartan | Méthyldopa | clonidine | doxazosine | atorvastatine | rosuvastatine | fénofibrate | ticagrélor | nicardipine | rivaroxaban)</td></tr>
</table>

	(contrefaçon | sous-standard | faux | falsifié | "qualité des médicaments" | adultéré | contaminé | impureté) AND (pindolol | nadolol | céliprolol | esmolol | carvédolol | félodipine | nimodipine | Vérapamil | diltiazem | périndopril | ramipril | quinapril | fosinopril | trandolapril | moexipril | nitroprussiate)
	(contrefaçon | sous-standard | faux | falsifié | "qualité des médicaments" | adultéré | contaminé | impureté) AND (phénoxybenzamine | moxisylyte | naftidrofuryl | "isosorbide mononitrate" | pravastatine | Fluvastatine | Bézafibrate | gemfibrozil | ciprofibrate | cholestyramine | colestipol | colesevelam | ézétimibe | dabigatran | alteplase | urokinase)
	(contrefaçon | sous-standard | faux | falsifié | "qualité des médicaments" | adultéré | contaminé | impureté) AND ("acide nicotinique" | acipimox | ézétimibe | argatroban | daltéparine | tinzaparine | Phénindione | Fluindione | acénocoumarol | dipyridamole | époprosténol | iloprost | eptifibatide | tirofiban | prasugrel | cilostazol | bivalirudine | apixaban)
	(contrefaçon | sous-standard | faux | falsifié | "qualité des médicaments" | adultéré | contaminé | impureté) AND (bosentan | ambrisentan | chlortalidone | métolazone | xipamide | indapamide | bumétanide | bumétanide | torasémide | éplerénone | "nicotinate d'inositol" | hexanicotinate | pentoxifylline | fondaparinux | retéplase | Ténectéplase)
**B. Medical devices**	
**Sources**	**English search Terms- Devices**
Pubmed	(spurious OR substandard OR falsified OR defective OR counterfeit OR non-compliant OR fake OR "device quality") AND ("Blood pressure device" OR Catheter OR "Arrhythmia detector" OR "heart valve" OR Pacemaker OR "cardiovascular device" OR "cardiovascular equipment" OR defibrillator OR "cardiac device" OR "cardiac equipment" OR "medical device" OR stent OR "Trace microsphere" OR "cardiac compressor" OR "Cardiopulmonary resuscitation" OR "counter-pulsating device" OR "rotating tourniquet" OR "endovascular graft" OR "Cardiopulmonary bypass" OR Electrocardiograph OR Phonocardiography OR "Left Ventricular Assist Device") NOT (gene OR protein OR cell OR channel)
Embase	((spurious or substandard or falsified or defective or counterfeit or "non-compliant" or fake or device quality) and ("Blood pressure device" or Catheter or "Arrhythmia detector" or "heart valve" or Pacemaker or "cardiovascular device" or "cardiovascular equipment" or defibrillator or "cardiac device" or "cardiac equipment" or "medical device" or stent or "Trace microsphere" or "cardiac compressor" or "Cardiopulmonary resuscitation" or "counter-pulsating device" or "rotating tourniquet" or "endovascular graft" or "Cardiopulmonary bypass" or Electrocardiograph or Phonocardiography or "Left Ventricular Assist Device"))
Web of Science	((spurious OR substandard OR falsified OR defective OR counterfeit OR non-compliant OR fake OR "device quality") AND ("Blood pressure device" OR Catheter OR "Arrhythmia detector" OR "heart valve" OR Pacemaker OR "cardiovascular device" OR "cardiovascular equipment" OR defibrillator OR "cardiac device" OR "cardiac equipment" OR "medical device" OR stent OR "Cardiopulmonary resuscitation" OR "counter-pulsating device" OR "endovascular graft" OR "Cardiopulmonary bypass" OR Electrocardiograph OR Phonocardiography OR "Left Ventricular Assist Device" OR "cardiac compressor"))
Google scholar	(spurious OR substandard OR falsified OR defective OR counterfeit OR non-compliant OR fake OR "device quality") AND ("Blood pressure device" OR Catheter OR "Arrhythmia detector" OR "heart valve" OR Pacemaker OR "cardiovascular device")
	(spurious OR substandard OR falsified OR defective OR counterfeit OR non-compliant OR fake OR "device quality") AND ("cardiovascular equipment" OR defibrillator OR "cardiac device" OR "cardiac equipment" OR "medical device" OR stent)
	(spurious OR substandard OR falsified OR defective OR counterfeit OR non-compliant OR fake OR "device quality") AND ("cardiac compressor" OR "Cardiopulmonary resuscitation" OR "counter-pulsating device")

<table>
<tr><td></td><td>(spurious OR substandard OR falsified OR defective OR counterfeit OR non-compliant OR fake OR “device quality”) AND (“Cardiopulmonary bypass” OR Electrocardiogram OR Phonocardiography OR “Left Ventricular Assist Device”)</td></tr>
<tr><td rowspan="2">Google</td><td>(spurious OR substandard OR falsified OR defective OR counterfeit OR non-compliant OR fake OR “device quality”) AND (stent OR “heart valve” OR Pacemaker OR defibrillator OR “Blood pressure device” OR Catheter OR “Arrhythmia detector” OR “cardiovascular device” OR "cardiovascular equipment" OR “cardiac device” OR “cardiac equipment”)</td></tr>
<tr><td>(spurious OR substandard OR falsified OR defective OR counterfeit OR non-compliant OR fake OR “device quality”) AND (“Cardiopulmonary resuscitation” OR “counter-pulsating device” OR “endovascular graft” OR “Cardiopulmonary bypass” OR Electrocardiograph OR Phonocardiography OR “Left Ventricular Assist Device” OR “cardiac compressor”)</td></tr>
<tr><td>Sources</td><td>French search Terms- Devices</td></tr>
<tr><td rowspan="2">Google</td><td>(faux | sous-standard | falsifié | défectueux | contrefait | "qualité du dispositif" | "non conforme") AND (Tensiomètre | "Mesure tension artérielle" | cathéter | "détecteur d'arythmie" | "valve cardiaque" | "stimulateur cardiaque" | "dispositif cardiovasculaire" | "équipement cardiovasculaire" | défibrillateur | "dispositif cardiaque" | "équipement cardiaque")</td></tr>
<tr><td>(faux | sous-standard | falsifié | défectueux | contrefait | "qualité du dispositif" | "non conforme") AND (stent | "réanimation cardio-pulmonaire"| "Greffe endovasculaire"| "dérivation cardio-pulmonaire"| électrocardiographe | "Compresseur cardiaque" | "Dispositif d'assistance ventriculaire" | Phonocardiographe)</td></tr>
<tr><td rowspan="4">Google Scholar</td><td>(faux OR sous-standard OR falsifié OR défectueux OR contrefait OR "qualité du dispositif" OR "non conforme") AND (Tensiomètre OR "Mesure tension artérielle" OR cathéter OR "détecteur d'arythmie" OR "valve cardiaque" OR "stimulateur cardiaque")</td></tr>
<tr><td>(faux OR sous-standard OR falsifié OR défectueux OR contrefait OR "qualité du dispositif" OR "non conforme") AND ("dispositif cardiovasculaire" OR "équipement cardiovasculaire" OR défibrillateur OR "dispositif cardiaque" OR "équipement cardiaque")</td></tr>
<tr><td>(faux OR sous-standard OR falsifié OR défectueux OR contrefait OR "qualité du dispositif" OR "non conforme") AND (stent OR "réanimation cardio-pulmonaire"OR "Greffe endovasculaire"OR "dérivation cardio-pulmonaire"OR électrocardiographe)</td></tr>
<tr><td>(faux OR sous-standard OR falsifié OR défectueux OR contrefait OR "qualité du dispositif" OR "non conforme") AND ("Compresseur cardiaque" OR "Dispositif d'assistance ventriculaire" OR Phonocardiographe)</td></tr>
</table>
