# Supplementary material for: The quality of medical products for cardiovascular diseases: a gap in global cardiac care
Source: BMJ Glob Health. 2021 Sep 14;6(9):e006523. doi: 10.1136/bmjgh-2021-006523 (PMC8442059; doi:10.1136/bmjgh-2021-006523)
Supplement: Supplementary data [file bmjgh-2021-006523supp002.pdf]

Supplementary file 2. Websites used for information searching about substandard and falsified cardiovascular medicines and medical devices

	Websites names and hyperlinks		Websites names and hyperlinks
International Organisations and NGOs	WHO-Essential Medicines and Health Products Information Portal	Medicine Regulatory Authorities and national bodies	Ghana Food and Drugs Authorities
	WHO Prequalification Programme		Kenya Pharmacy and Poisons Board
	USP Promoting the Quality of Medicines		Hong Kong Government
	Safe Medicines India		NAFDAC Nigeria
			Danish medicines agency
	MIMS thailand		Thailand Food and Drugs Administration
	Office des nations unies contre la drogue et le crime		Agence Nationale de Sécurité du Médicament et des Produits de Santé
	ReMeD-Réseau Médicaments et Développement		Medicines and Healthcare products Regulatory Agency, UK Government
	Medical Products Counterfeiting and Pharmaceutical Crime (INTERPOL)		US Food and Drug Administration
	ACG - Anti-counterfeting group		Government of Canada
	Permanent Forum on International Pharmaceutical Crime		Central Drugs Standard Control Organization
	Medicines Transparency Alliance- Health Action International		Ordre national des pharmaciens de côte d'ivoire
	Médecins Sans Frontières Access Campaign	Alert lists and systems	
	Third World Network		Campaign for Safe Medicines in Kenya
	Council of Europe-Medicrime convention		Sproxil Brand Protection & Mobile Sales Enablement Solution
	EAASM-European Alliance for Access to Safe Medicines		Association Développement et Santé Contrefaçon Riposte
	GACG Global Anti-Counterfeiting Network		Mpedigree - Bringing Quality To Life
	IRACM Institute of Research Against Counterfeit Medicines		Partnership for safe medicines
	The Global Pharma Health Fund		Pharmabiz
	Fondation Chirac - Agir au service de la paix		PharmaSecure
Academic/ Research Initiatives	QUAMED - Quality Medicines for All	Newspaper websites with	www.ghanaweb.com
	IRASEC- Institut de Recherche sur l'Asie du Sud-Est Contemporaine		https://www.modernghana.com/
	Pharmelp Detection of counterfeit medicines		

	The pharmaceutical security institute	**interest in medicine quality**	https://www.thehansindia.com/
**Pharmaceutical Industry**	Securing Pharma		https://www.chiangraitimes.com/
	Reconnaissance international		https://www.monitor.co.ug/
	Pfizer Pharmaceutical News		
	Sanofi		
