# Supplementary material for: The quality of medical products for cardiovascular diseases: a gap in global cardiac care
Source: BMJ Glob Health. 2021 Sep 14;6(9):e006523. doi: 10.1136/bmjgh-2021-006523 (PMC8442059; doi:10.1136/bmjgh-2021-006523)
Supplement: Supplementary data [file bmjgh-2021-006523supp004.pdf]

**Supplementary file 4 :** Number of publication, data points and samples per study/report type in publications containing description of the quality of cardiovascular medicines in data point(s), i.e. in a specific location at a specific time				
	**Study type**	**No. publications n (%)**	**No. data points n (%)**	**No. samples n (%)**
**Scientific reports**	**Equivalence study**	116 (48.1%)	161 (33.0%)	822 (17.5%)
	**Prevalence survey**	27 (11.2%)	131 (26.8%)	3,414 (72.6%)
	**Control quality - routine**	14 (5.8%)	51 (10.5%)	443 (9.4%)
	**Stability study**	5 (2.1%)	6 (1.2%)	18 (0.4%)
	**Bioavailability studies**	2 (0.8%)	2 (0.4%)	6 (0.1%)
	**Total**	**164 (68.0%)**	**351 (72.1%)**	**4,703 (100.0%)**
**Other reports**	**Recall/warning/alert**	65 (27.0%)	124 (25.4%)	
	**Case reports**	6 (2.5%)	6 (1.2%)	
	**Seizure**	6 (2.5%)	7 (1.4%)	
	**Total**	**77 (32.0%)**	**137 (28.1%)**	
**Total**		**241 (100.0%)**	**488 (100.0%)**	
