# Supplementary material for: The quality of medical products for cardiovascular diseases: a gap in global cardiac care
Source: BMJ Glob Health. 2021 Sep 14;6(9):e006523. doi: 10.1136/bmjgh-2021-006523 (PMC8442059; doi:10.1136/bmjgh-2021-006523)
Supplement: Supplementary data [file bmjgh-2021-006523supp005.pdf]

Supplementary file 5. Main characteristics of prevalence surveys of cardiovascular medicines

Reference / country of collection	Active pharmaceutical ingredient (API)	Brand name	Outlet/ Sampling type	Reference specifications standard	Test (s) performed and analytical technique	No. Failed n/N(%)
Nazerali, 1996 [1] Zimbabwe	Epinephrine	Unknown	Public medical stores, hospital and health centres/ Convenience	USP 1990	API content<br><br>*HPLC*	2 /11(18.2%)
Westenberger, 2005 [2] Unstated country	Warfarin	Unknown	Website/ Convenience	USP 26/ 2003	API content, API Identification, Dissolution, Impurity, Weight variation, Visual inspection<br><br>*HPLC, Near-infrared spectroscopy (NIR), Thermogravimetric (TGA)*	0 /4(0%)
Schwertner, 2005 [3] Mexico	Digoxin	Lanoxin	Pharmacies/ Convenience	USP	API content<br><br>*HPLC-DAD*	0/2 (0%)
Sheth, 2007 [4] India	Ramipril<br>Atenolol<br>Diltiazem	Unknown	Retail pharmacy (licenced) / Convenience	' Conduct of analysis as per Pharmacopoeial status mentioned on label.'	API content<br><br>*Method Unstated*	0/86 (0.0%)
Qureshi, 2008 [5] Argentina, Austria, Brazil, Canada, China, Cyprus, Denmark, Egypt, Finland, France, Germany, Greece, Hungary, Luxembourg, Mexico, Netherlands, New Zealand, Poland, Portugal	Furosemide	Lasix and other products	Different outlets (retail pharmacy, wholesaler)/ Convenience	BP<br>USP 22<br>European Pharmacopoeia	API content, Content uniformity, Disintegration, Dissolution, Mass uniformity<br><br>*HPLC-UV*<br>*IR spectrophotometry*	3/196 (1.5%)

Reference / country of collection	Active pharmaceutical ingredient (API)	Brand name	Outlet/ Sampling type	Reference specifications standard	Test (s) performed and analytical technique	No. Failed n/N(%)
CDSCO, 2009 [6] India	Amlodipine, Atenolol, Atorvastatin	Amdepine Amlopress Amlosafe Aten-50 Atherochek10 Storvas-10 Tenolol Ziblok Zivast-10	Hospital/health centers	Indian Pharmacopeia	API content, Api Identification, Visual inspection *Method Unstated*	3/435 (0.7%)
Twagirumukiza, 2009 [7] Rwanda	Atenolol Captopril Hydrochlorothiazide Methyldopa Propranolol	Tenormin, Betanorm Catenol Capoten Captopril Denk, Cetopril Esidrex, HCTZ L&A, HCTZ Cipla Aldomet Belgium Methyldopa L&A Aldomet Pakistan Inderal Cepanolol	Public and official private pharmacies/ Convenience	USP 2006	API content, Dissolution *HPLC-UV/Vis* *UV-visible spectrophotometry*	2 /16(12.5%)
Markman, 2009 [8] Brazil	Simvastatin	Unknown	Compounding pharmacies/ Convenience	Brazilian Pharmacopeia, USP 2008	API content, Content uniformity, Dissolution, weight *HPLC-UV/Vis*	16/18 (88.9%)
Bate, 2010 [9] United States	Atorvastatin	Lipitor	Website/ Convenience	Not applicable	API content *Raman Spectroscopy*	0/25 (0.0%)
Luciane O. Lima, 2011 [10] Brazil	Methyldopa Enalapril Propranolol Furosemide	Unknown	Pharmacy/ Convenience	Brazilian Pharmacopeia	API content, content uniformity, weight, dissolution *UV-visible spectrophotometry*	3/9 (33.3%)
Marques-Marinho, 2011 [11] Brazil	Simvastatin	Unknown	Compounding pharmacies/ Convenience	Brazilian Pharmacopeia, USP 2009	API content, Content uniformity, Disintegration, Dissolution, Friability, Hardness, Mass uniformity *RP-HPLC-UV/DAD*	16 /30 (53.3%)

Reference / country of collection	Active pharmaceutical ingredient (API)	Brand name	Outlet/ Sampling type	Reference specifications standard	Test (s) performed and analytical technique	No. Failed n/N(%)
Yusuf, Inua, 2011 [12] Afghanistan	Atenolol	Unknown	Public and private pharmacy/ Convenience	United States Pharmacopeia	API content, API identification, Dissolution<br><br>*HPLC*	3/30 (10.0%)
Karlage, 2012 [13]<br>US, Mexico	Warfarin	Generic<br>Coumadin<br>Rofarmin	Pharmacy / Random	USP	API content<br><br>*HPLC-DAD*	0/4 (0.0%)
Patel, 2012 [14]<br><br>South Africa	Hydrochlorothiazide (HCTZ)	Adco-retic<br>HCTZ<br>Ridaq<br>Hexazide	Public and private health sectors outlets-unspecified/ Convenience	BP 2005, USP 2006	API identification, Dissolution test, Uniformity of weight<br><br>*Fourier transformed infrared spectroscopy (FTIR)*	0/4 (0.0%)
Kahsay, 2013 [15] Belgium	Clopidogrel-Acetylsalicylic Acid	Duoplavin<br>Coplavix<br>Clopitab<br>Clopilet<br>Plagril<br>Clavix<br>Noklot<br>Deplatt, Dospin<br>Antiplar	Unknown/Unknown	European Pharmacopoeia and USP 2010	API content, impurity, dissolution, content uniformity<br><br>*Liquid Chromatography (LC)-UV/Visible photodiode array*	8 /10(80%)
Al-Qatamin, 2014 [16]<br>Jordan	Clopidogrel<br>Atorvastatin<br>Bisoprolol<br>Valsartan-HCTZ	Plavix,<br>Lipitor<br>Concor<br>Co-diovan	Pharmacies/ Random and convenience	USP	API content, Visual inspection, Impurity<br><br>*HPLC-UV*	0/172 (0%)
Fukami, 2015 [17] Japan	Atorvastatin	Lipitor<br>Lipiget<br>Atorlip<br>Lipvas<br>Aztor	Website/ Convenience	Japanese Pharmacopeia	API content, Visual inspection, Dissolution<br><br>*HPLC*	1/6 (16.7%)
Macquart De Terline, 2016 [18]<br>*Benin, Burkina Faso, Congo-Brazzaville, Côte D'Ivoire, Guinea,*	Acenocoumarol<br>Amlodipine<br>Atenolol<br>Captopril<br>Furosemide<br>Hydrochlorothiazide<br>Simvastatin	Unknown	Private pharmacy and street market/ Random and Convenience	In-house methods and specifications	API content<br><br>*RP-HPLC-MS/MS*	249/1530 (16.3%)

Reference / country of collection	Active pharmaceutical ingredient (API)	Brand name	Outlet/ Sampling type	Reference specifications standard	Test (s) performed and analytical technique	No. Failed n/N(%)
Mauritania, Niger, the Democratic Republic of the Congo, Senegal, and Togo						
Petersen, 2017 [19] *Cameroon, Democratic Republic of the Congo, Nigeria, Kenya, Uganda, Ghana, India*	Captopril<br>Atenolol<br>Bisoprolol<br>Furosemide<br>Hydrochlorothiazide	Unknown	Private outlet (formal and informal drug vendors)/ Convenience	BP 2015	API content, API identification, Disintegration<br><br>*Colorimetry, Thin Layer Chromatography (TLC)*	1/50 (2.0%)
Schafermann, 2018 [20] Togo	Atenolol<br>Furosemide<br>Hydrochlorothiazide	Atenolol Denk<br>Furo Denk<br>Furosemide<br>Esidrex<br>HCTZ	Licensed pharmacy/ informal vendor/ Convenience	USP 2016	API content, Visual inspection, Dissolution<br><br>*HPLC-UV*	1/24 (4.8%)
Kakio, 2018 [21] China, Myanmar, Indonesia	Candesartan	Blopress<br>XINXIN<br>Candelong-8<br>Advant	Hospital/ Pharmacy/ Convenience	Japanese Pharmacopeia	API content, Visual inspection, Dissolution*<br><br>*HPLC-UV*	19/37 (51.4%)
Ashames, 2019 [22] Libya	Atenolol<br>Furosemide	Unknown	Local pharmacies/ Convenience	BP 2015	API Content, Weight uniformity, Hardness, Friability, Disintegration, Dissolution<br><br>*UV/Visible spectrophotometer*	0/9 (0%)
Ndichu, 2019 [23] Nigeria	Nifedipine	Unknown	Registered pharmacies/ Random	International Pharmacopeia, USP, Food and Drug Administration	API content, Visual inspection, Impurity<br><br>*HPLC-MS*	78/102 (76.5%)
Julie Redfern, 2019 [24] Nigeria	Amlodipine<br>Lisinopril	Unknown	Private pharmacies/ Random	United States Pharmacopeia	API content, Visual inspection<br><br>*HPLC-PDA*	101/361 (28.0%)
Rahman, M.S, 2019 [25]	Amlodipine	Unknown	Pharmacy, Depot/	British Pharmacopoeia,	API content, Content uniformity, Dissolution	7/79 (8.9%)

**Reference / country of collection**	**Active pharmaceutical ingredient (API)**	**Brand name**	**Outlet/ Sampling type**	**Reference specifications standard**	**Test (s) performed and analytical technique**	**No. Failed n/N(%)**
Cambodia			Convenience	United States Pharmacopeia	*HPLC-DAD*	
Khurelbat. D, 2020 [26] Mongolia	Amlodipine	Unknown	Pharmacy oulets/ Random	British Pharmacopoeia, Pharmacopeia of the People's Republic of China, Mongolian National Pharmacopoeia	API content, Visual inspection, Weight variation, Disintegration, Friability, Dissolution<br><br>*HPLC-UV*	8/118 (6.8%)
Sakuda, Mirai, 2020 [27] Myanmar	Candesartan	Unknown	Hospitals, pharmacies, and wholesalers/ Unspecified	British Pharmacopoeia, United State Pharmacopoeia, Japanese Pharmacopoeia	API content, Content uniformity, Dissolution<br><br>*Method Unstated*	1/10 (10.0%)

RP, reverse phase; HPLC, High-performance liquid chromatography; MS, Mass spectrometry; UV, Ultraviolet; TLC, Thin Layer Chromatography; FTIR, Fourier transformed infrared spectroscopy; LC, Liquid Chromatography; DAD, Diode-Array Detection; NIR, Near-infrared spectroscopy, TGA, Thermogravimetric; HTCZ, Hydrochlorothiazide; BP, British Pharmacopeia; USP, United Stated Pharmacopeia; API, Active Pharmaceutical Ingredient
*Samples were tested by both high-performance liquid chromatography (HPLC) and Raman spectrophotometry, with inconsistent results between the two techniques. Only the reference HPLC results were retained in our analysis.

1 Nazerali H, Machemwa T, Hogerzeil H. Stability of essential drugs in tropical climates (Zimbabwe). https://apps.who.int/medicinedocs/pdf/s2206e/s2206e.pdf (accessed 20 Sep 2019).

2 Westenberger BJ, Ellison CD, Fussner AS, *et al.* Quality assessment of internet pharmaceutical products using traditional and non-traditional analytical techniques. *Int J Pharm* 2005;**306**:56–70. doi:10.1016/j.ijpharm.2005.08.027

3 Schwertner HA, Storrow AB. Comparison of actual and stated concentrations of pharmaceuticals manufactured in Mexico. *Clin Ther* 2005;**27**:609–15. doi:10.1016/j.clinthera.2005.05.007

4 Extent of Spurious (Counterfeit) Medicines in India. https://drive.google.com/file/d/1W_vTtByEOFlV6QQHoHvETCInPz6IGCbu/view (accessed 3 Jun 2019).

5 Qureshi S, Industrial IM-D development and, 1998 U. Assessment of pharmaceutical quality of furosemide tablets from multinational markets. *Taylor Fr* doi:https://doi.org/10.3109/03639049809089943

6 CDSCO G of I. Report on countrywide survey for spurious drugs. Cent. Drugs Stand. Control Organ. CDSCO, India. 2009.https://cdsco.gov.in/opencms/opencms/system/modules/CDSCO.WEB/elements/download_file_division.jsp?num_id=MTU1Nw== (accessed 12 Dec 2020).

7 Twagirumukiza M, Pringels E, Remon JP, *et al.* Influence of Tropical Climate Conditions on the Quality of Antihypertensive Drugs from Rwandan Pharmacies. *Am J Trop Med Hyg* 2009;**81**:776–81. doi:10.4269/ajtmh.2009.09-0109

8 Markman BEO, Rosa PCP, Koschtschak MRW, *et al.* Assessment of the quality of simvastatin capsules from compounding pharmacies. *Rev Saude Publica* 2010;**44**:1055–62. doi:http://dx.doi.org/10.1590/S0034-89102010000600010

9 Bate R. Assessing website pharmacy drug quality: Safer than you think? *PLoS One* 2010;**5**:e12199. doi:http://dx.doi.org/10.1371/journal.pone.0012199

10 Lima L., Benetoli A, Gianotto E, *et al.* Quality evaluation of compounded capsules. *Lat Am J Pharm* 2011;**30**:1943.http://www.latamjpharm.org/trabajos/30/10/LAJOP_30_10_1_11_R1QY9O4WS3.pdf

11 Marques-Marinho FD, Zanon JCD, Sakurai E, *et al.* Quality evaluation of simvastatin compounded capsules. *Brazilian J Pharm Sci* 2011;**47**:495–502. doi:http://dx.doi.org/10.1590/S1984-82502011000300007

12 Yusuf I, Lee D, Fatehzada Z, *et al.* Afghanistan Medicines Sampling and Testing - A Quantitative Survey | Management Sciences for Health. Manag. Sci. Heal. 2011.https://www.msh.org/resources/afghanistan-medicines-sampling-and-testing-a-quantitative-survey (accessed 12 Dec 2020).

13 Karlage KL, Franklin SJ, Mufich WC, *et al.* Comparative evaluation of pharmaceutical products obtained in Mexico: Augmenting existing scientific data. *Drug Dev Ind Pharm* 2012;**38**:808–14. doi:http://dx.doi.org/10.3109/03639045.2011.628678

14 A. P, R. G, P. N, *et al.* Quality of generic medicines in South Africa: perceptions versus reality - a qualitative study. *BMC Health Serv Res* 2012;**12**:297. doi:10.1186/1472-6963-12-297

15 Kahsay G, Dhulipalla RL, Van Nyen G, *et al.* Analysis of purity of clopidogrel and acetylsalicylic acid in combined oral dosage forms. *Curr Pharm Anal* 2013;**9**:82–91. doi:DOI: 10.2174/157341213804806070

16 Al-Qatamin S, A. The evaluation and analysis of counterfeit pharmaceuticals within Jordan. Published Online First: 2014.https://core.ac.uk/download/pdf/30617878.pdf

17 Fukami T, Koide T, Hisada H, *et al.* Pharmaceutical evaluation of atorvastatin calcium tablets available on the Internet: A preliminary investigation of substandard medicines in Japan. *J Drug Deliv Sci Technol* 2016;**31**:35–40. doi:http://dx.doi.org/10.1016/j.jddst.2015.11.006

18 Antignac M, Diop B. *Quality assessment of 7 cardiovascular drugs in 10 sub-Saharan countries: The SEVEN study*. M. Antignac, Department of Pharmacy, Saint-Antoine Hospital, East Paris University Hospitals, AP-HP, 184 Rue du Faubourg Saint-Antoine, Paris 75012, France. E-mail: marie.antignac@aphp.fr: : American Medical Association (E-mail: smcleod@itsa.ucsf.edu) 2017. doi:http://dx.doi.org/10.1001/jamacardio.2016.3851

19 Petersen A, Held N. Surveillance for falsified and substandard medicines in Africa and Asia by local organizations using the low-cost GPHF

Minilab. *PLoS One* 2017;**12**:e0184165. doi:http://dx.doi.org/10.1371/journal.pone.0184165

20 Schafermann S, Wemakor E, Hauk C. Quality of medicines in southern Togo: Investigation of antibiotics and of medicines for non-communicable diseases from pharmacies and informal vendors. *PLoS One* 2018;**13**:e0207911. doi:http://dx.doi.org/10.1371/journal.pone.0207911

21 Kakio T, Nagase H, Takaoka T, *et al.* Survey to identify substandard and falsified tablets in several Asian countries with pharmacopeial quality control tests and principal component analysis of handheld raman spectroscopy. *Am J Trop Med Hyg* 2018;**98**:1643–52. doi:http://dx.doi.org/10.4269/ajtmh.17-0553

22 Ashames A, Abushoffa A, Tabet M, *et al.* Impact of the Libyan Conflict on Quality of Medicines Available in the Local Market. *Available online www.jocpr.com J Chem Pharm Res* 2019;**11**:20–7.http://www.jocpr.com/articles/impact-of-the-libyan-conflict-on-quality-of-medicines-available-in-the-local-market.pdf

23 Ndichu E., Ohiri K, Sekoni O, *et al.* Evaluating the quality of antihypertensive drugs in Lagos State, Nigeria. *PLoS One* 2019;**14**. doi:http://dx.doi.org/10.1371/journal.pone.0211567

24 Redfern J, Kaur H, Adedoyin RA, *et al.* Equivalence in Active Pharmaceutical Ingredient of Generic Antihypertensive Medicines Available in Nigeria (EQUIMEDS): A Case for Further Surveillance. *Glob Heart* 2019;**14**:327–33. doi:10.1016/j.gheart.2019.07.006

25 Rahman MS, Yoshida N, Tsuboi H, *et al.* A cross-sectional investigation of the quality of selected medicines for noncommunicable diseases in private community drug outlets in Cambodia during 2011-2013. *Am J Trop Med Hyg* 2019;**101**:1018–26. doi:http://dx.doi.org/10.4269/ajtmh.19-0247

26 Khurelbat D, Dorj G, Sunderland B, *et al.* A cross-sectional analysis of falsified, counterfeit and substandard medicines in a low-middle income country. *BMC Public Health* 2020;**20**:743. doi:10.1186/s12889-020-08897-x

27 Sakuda M, Yoshida N, Takaoka T, *et al.* Substandard and Falsified Medicines in Myanmar. *Pharmacy* 2020;**8**:45. doi:10.3390/pharmacy8010045
