# Supplementary material for: The quality of medical products for cardiovascular diseases: a gap in global cardiac care
Source: BMJ Glob Health. 2021 Sep 14;6(9):e006523. doi: 10.1136/bmjgh-2021-006523 (PMC8442059; doi:10.1136/bmjgh-2021-006523)
Supplement: Supplementary data [file bmjgh-2021-006523supp006.pdf]

Supplementary file 6: Failure frequency per type of quality test performed in prevalence surveys of cardiovascular medicines quality

Because of the limited number of samples tested for quality in the studies included in this review, the figures should not be interpreted as representative of the prevalence of specific SF cardiovascular medicines (please refer to the discussion section of the current paper for more details)

Quality attribute test	Failure Frequency n/N (%)
**Impurity/Contaminant/Related substance**	16.5% (80/484)
**API content**	13.1% (430/3,293)
**Uniformity of units** *	8.4% (52/ 616)
**Dissolution**	4.2% (28/659)
**Packaging**	0.3% (4/1,335)
**Other chemical analysis****	0.0% (0/641)
**Other physical analysis*****	0.5% (5/974)

**Includes: content uniformity, weight uniformity, uniformity of mass, weight variation*
***Includes, spectral comparative compositional analysis (vs authentic or other brands), identification of APIs, API semi-quantitation*
****Includes friability, hardness, disintegration*

Note: One sample may have been tested for one or more quality tests
Failure frequency is defined as the proportion of samples that failed at least one quality test described in the report
