# Supplementary material for: The quality of medical products for cardiovascular diseases: a gap in global cardiac care
Source: BMJ Glob Health. 2021 Sep 14;6(9):e006523. doi: 10.1136/bmjgh-2021-006523 (PMC8442059; doi:10.1136/bmjgh-2021-006523)
Supplement: Supplementary data [file bmjgh-2021-006523supp007.pdf]

Supplementary file 7: Samples collected by type of outlet in prevalence surveys of cardiovascular medicines quality

Because of the limited number of samples tested for quality in the studies included in this review, the figures should not be interpreted as representative of the prevalence of specific SF cardiovascular medicines (please refer to the discussion section of the current paper for more details)

Failure frequency is defined as the proportion of samples that failed at least one quality test

Type of outlet	Failure frequency n/N (%)
**Hospital/health centers**	44.4% (16/36)
**Private pharmacy**	19.8% (349/1,762)
**Unlicensed/unregistered outlets**	19.7% (129/656)
**Website**	2.9% (1/35)
**Wholesalers/importer/distributors**	0.0% (0/9)
**Unknown**	3.3% (30/917)
**Total**	**15.4% (525/3,414)**
