# Supplementary material for: The quality of medical products for cardiovascular diseases: a gap in global cardiac care
Source: BMJ Glob Health. 2021 Sep 14;6(9):e006523. doi: 10.1136/bmjgh-2021-006523 (PMC8442059; doi:10.1136/bmjgh-2021-006523)
Supplement: Supplementary data [file bmjgh-2021-006523supp009.pdf]

Supplementary file 9. Frequency of agreement with the 26 items of the MEDQUARG checklist of the 21 prevalence surveys of cardiovascular medicines quality

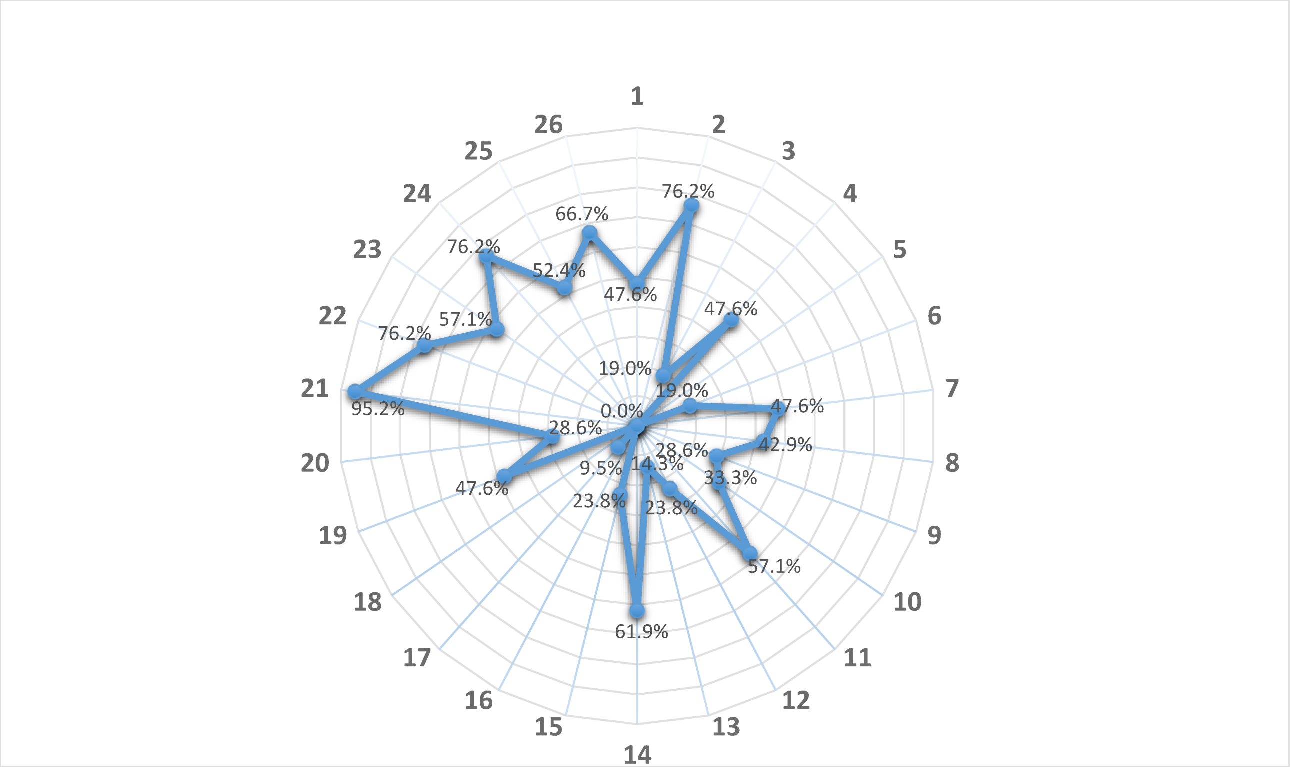

Definition of items: 1. Title/abstract/keywords 2. Introduction.
Methods (items 3-13): 3. Survey details 4. Definitions 5. Outlets 6. Sampling design 7. Samplers 8. Statistical methods 9. Ethical issues 10. Packaging 11. Chemical analysis 12. Method validation 13. Blinding. Results (items 14-20): 14. Outlets (actual) 15. Missing samples 16. Packaging and chemistry results 17. Category of poor quality medicine 18. State company and address as given on packaging 19. Sharing data with MRA 20. Dissemination. Discussion (items 21-24): 21. Key results 22. Limitations 23. Interpretation 24. Intervention. Declaration: 25. Conflict of interest 26. Funding.

No	Item	Bate & Hess, 2010	SEARPharm, 2007	Karlage et al, 2011	Westendberger et al, 2005	Difam-EPN Minilab, 2017	Twagirumukiza et al, 2009	Schafermann et al, 2018	Lima et al, 2011
1	Title/abstract/keywords	N	N	Y	Y	N	N	Y	Y
2	Introduction	Y	Y	Y	N	Y	Y	Y	Y
**Methods**									
3	Survey details	Y	N	N	N	N	N	N	N
4	Definitions	N	Y	Y	N	Y	N	Y	N
5	Outlets	N	N	N	N	N	N	N	N
6	Sampling design	Y	Y	N	N	N	N	Y	N
7	Samplers	Y	Y	N	N	Y	N	Y	N
8	Statistical methods	N	N	N	N	N	N	Y	N
9	Ethical issues	N	N	N	N	N	N	N	N
10	Packaging	N	Y	N	Y	N	N	N	N
11	Chemical analysis	Y	N	N	Y	Y	N	Y	N
12	Method validation	N	N	N	N	N	N	Y	N
13	Blinding	N	N	N	N	N	Y	N	N
**Results**									
14	Outlets	Y	N	N	Y	N	Y	Y	N
15	Missing samples	Y	Y	N	N	N	N	Y	N
16	Packaging and chemistry results	N	N	N	N	N	N	N	N
17	Category of poor-quality medicine	N	N	N	N		N	N	N
18	State company and address as given on packaging	N	N	N	N	N	N	N	N
19	Sharing data with MRA	N	Y	N	Y	Y	N	N	N
20	Dissemination	N	N	N	N	Y	N	N	N
**Discussion**									
21	Key results	N	Y	Y	Y	Y	Y	Y	Y
22	Limitations	Y	Y	Y	Y	Y	Y	Y	N
23	Interpretation	N	Y	N	Y	Y	N	Y	N
24	Intervention	Y	N	N	N	Y	Y	Y	Y

No	Item	Bate & Hess, 2010	SEARPharm, 2007	Karlage et al, 2011	Westendberger et al, 2005	Difam-EPN Minilab, 2017	Twagirumukiza et al, 2009	Schafermann et al, 2018	Lima et al, 2011
**Other Information**									
25	Conflict of interest	Y	N	Y	N	Y	Y	Y	N
26	Funding	Y	N	N	N	Y	Y	Y	N
	Total score	11	10	6	8	12	8	16	4
		42.3%	38.5%	23.1%	30.8%	46.2%	30.8%	61.5%	15.4%

Y : Yes, N : No

No	Item	Fukami et al, 2015	Macquart de Terline et al, 2017	Marques-Marinho et al, 2011	Schwertner & Storrow, 2005	Qureshi & McGilveray, 1998	Ashames et al, 2019	Al-Qatamin, 2012	Ndichu et al, 2019
1	Title/abstract/keywords	N	Y	N	Y	N	N	N	N
2	Introduction	Y	N	N	Y	N	Y	Y	N
**Methods**									
3	Survey details	N	Y	N	N	N	N	N	N
4	Definitions	Y	Y	N	N	N	N	Y	Y
5	Outlets	N	N	N	N	N	N	N	N
6	Sampling design	N	N	N	N	N	N	N	N
7	Samplers	N	Y	N	N	N	N	N	Y
8	Statistical methods	N	Y	Y	N	Y	N	N	Y
9	Ethical issues	N	N	N	Y	N	N	N	Y
10	Packaging	Y	N	N	N	N	N	Y	N
11	Chemical analysis	N	Y	N	N	Y	N	Y	N
12	Method validation	N	Y	Y	Y	N	N	N	N
13	Blinding	N	Y	N	N	N	N	N	N
**Results**									
14	Outlets	Y	Y	Y	N	N	N	N	Y
15	Missing samples	N	Y	N	N	N	N	N	Y
16	Packaging and chemistry results	N	N	N	N	N	N	N	N
17	Category of poor-quality medicine	N	N	N	N	N	N	Y	N

No	Item	Fukami et al, 2015	Macquart de Terline et al, 2017	Marques-Marinho et al, 2011	Schwertner & Storrow, 2005	Qureshi & McGilveray, 1998	Ashames et al, 2019	Al-Qatamin, 2012	Ndichu et al, 2019
18	State company and address as given on packaging	N	N	N	N	N	N	N	N
19	Sharing data with MRA	N	Y	N	N	Y	N	Y	N
20	Dissemination	Y	N	N	N	N	N	Y	Y
**Discussion**									
21	Key results	Y	Y	Y	Y	Y	Y	Y	Y
22	Limitations	N	Y	N	Y	N	N	Y	Y
23	Interpretation	N	Y	N	Y	N	N	Y	N
24	Intervention	Y	Y	N	Y	N	Y	Y	Y
**Other Information**									
25	Conflict of interest	N	Y	N	N	N	Y	N	Y
26	Funding	Y	Y	Y	N	N	Y	N	Y
	Total score	8	17	5	8	4	5	11	12
		30.8%	65.4%	19.2%	30.8%	15.4%	19.2%	42.3%	46.2%

Y : Yes, N : No

No	Item	Khurelbat et al, 2020	Sakuda, M et al, 2020	Julie Redfern et al, 2019	Yusuf I et al, 2011	Rahman M.S et al, 2019
1	Title/abstract/keywords	Y	N	Y	Y	Y
2	Introduction	Y	Y	Y	Y	Y
**Methods**						
3	Survey details	Y	N	N	Y	N
4	Definitions	Y	N	Y	N	N
5	Outlets	N	N	N	N	N
6	Sampling design	Y	N	N	N	N
7	Samplers	Y	N	Y	Y	Y
8	Statistical methods	Y	Y	Y	N	Y

No	Item	Khurelbat et al, 2020	Sakuda, M et al, 2020	Julie Redfern et al, 2019	Yusuf I et al, 2011	Rahman M.S et al, 2019
9	Ethical issues	Y	Y	Y	N	Y
10	Packaging	Y	Y	N	N	Y
11	Chemical analysis	Y	Y	Y	Y	Y
12	Method validation	N	N	N	N	Y
13	Blinding	N	N	N	Y	N
**Results**						
14	Outlets	Y	Y	Y	Y	Y
15	Missing samples	N	N	N	N	N
16	Packaging and chemistry results	N	N	N	N	N
17	Category of poor-quality medicine	N	N	Y	N	N
18	State company and address as given on packaging	N	N	N	N	N
19	Sharing data with MRA	Y	Y	N	Y	Y
20	Dissemination	Y	Y	N	N	N
**Discussion**						
21	Key results	Y	Y	Y	Y	Y
22	Limitations	Y	Y	Y	Y	Y
23	Interpretation	Y	Y	Y	Y	Y
24	Intervention	Y	Y	Y	Y	Y
**Other Information**						
25	Conflict of interest	Y	Y	Y	N	N
26	Funding	Y	Y	Y	Y	Y
	Total score	19	14	15	13	15
		73.1%	53.8%	57.7%	50.0%	57.7%

Y : Yes, N : No
