# Supplementary material for: The quality of medical products for cardiovascular diseases: a gap in global cardiac care
Source: BMJ Glob Health. 2021 Sep 14;6(9):e006523. doi: 10.1136/bmjgh-2021-006523 (PMC8442059; doi:10.1136/bmjgh-2021-006523)
Supplement: Supplementary data [file bmjgh-2021-006523supp010.pdf]

Supplementary file 10. Characteristics of equivalence surveys of cardiovascular medicines

Reference / country of collection	Active pharmaceutical ingredient (API)	Brand name	Outlet	Reference standard	Test(s) performed and analytical technique for API content/Impurities	Failure frequency n/N (%)
Julide Akbuga, 1987 [1]<br>Turkey	Furosemide	Unknown	Unknown	USP 20	API Content, Weight uniformity, Hardness, Friability, Disintegration, Dissolution<br><br>*Thin-Layer Chromatography TLC*	2 /9(22.2%)
S Sakolkhai, 1991 [2]<br>Thailand	Spironolactone	Generic	Hospital and drug stores	USP	API content and Dissolution<br><br>*HPLC (unknown detector)*	2/3 (66.7%)
L. Nikolic, 1994 [3]<br>Serbia	Acetylsalicylic acid	Unknown	Unknown	USP 1985	API content, dissolution, weight variation, disintegration time, crushing strength and friability<br><br>*HPLC-DAD*	6/6 (100.0%)
Baloglu , 2001 [4]<br>Turkey	Enalapril	Generic	Pharmacies	USP	API content, Weight Variation, Hardness, Disintegration, Diameter-Thickness Ratio, Friability, Content Uniformity, Dissolution<br><br>*UV-Spectrophotometry*	8/8 (100.0%)
Ming-shin L, 2010 [5]<br>Taiwan	Hydrochlorothiazide	Unknown- 15 domestic products and one imported from the UK	Unknown	USP	Dissolution, Content Uniformity<br><br>*HPLC-UV*	10/16 (62.5%)
Gomez , 2003 [6]<br>Asia and South America	Clopidogrel	Plagril<br>Clodrel®<br>Orawis®<br>Noklot®<br>Clopigrel®<br>Preva®<br>Clavix®<br>Clopilet®<br>Stromix®<br>Cloplat-75®<br>Deplatt®<br>Ceruvin	Unknown	European Pharmacopeia 2002 +<br>USP 2000	API content, Dissolution, Impurity<br><br>*HPLC* (unknown detector)<br>*LC* (unknown detector)	16 /19 (84.2%)

**Reference / country of collection**	**Active pharmaceutical ingredient (API)**	**Brand name**	**Outlet**	**Reference standard**	**Test(s) performed and analytical technique for API content/Impurities**	**Failure frequency n/N (%)**
		Cloplatic®<br>Plagrel®<br>Nefazan®<br>Talcom®<br>Clopifran®				
Karl-Uwe Petersen, 2003 [7]<br>Germany	Felodipine	Generic	Pharmacies	USP 22	API content and Dissolution<br><br>*UV- Spectroscophotometry*	0 /7(0.0%)
C Longstaff, 2004 [8]<br>Multicountry L/MIC countries	Streptokinase	Unknown	Unknown	European pharmacopeia<br>BP	API content<br><br>*Chromogenic methods*	15 /18(83.3%)
Teshome Dires, 2005 [9]<br>Ethiopia	Methyldopa<br>Propranolol<br>Furosemide	Generic<br>Aldomet<br>Dopegyt<br>Inderal<br>Emforal<br>Lasix<br>Fusix	Private pharmacies	BP 2001,<br>USP 27	API Content, Weight uniformity, Hardness, Friability, Disintegration, Dissolution<br><br>*UV/Vis spectrophotometry*	1/9 (11.1%)
Smith, 2005 [10]<br>Serbia and Montenegro	Carvedilol	Carbetalol<br>Carloc<br>Carvedilol<br>Carvista<br>Coritensil<br>Diola<br>Karvedilol<br>Talliton<br>Vivacor<br>Coryol<br>Karvileks<br>Milenol<br>Talliton<br>Coronat<br>Longcardio<br>V-Block-PT	Distributors	European Pharmacopeia + Roche specifications + USP	API content, Dissolution, Impurity, Hardness<br><br>*HPLC-UV*<br>*UV-spectrophotometry*	17/35 (44.7%)
Hermentin, 2005 [11]<br>Brazil, India, Jordan, Pakistan	Streptokinase	Akinase<br>Unitinase<br>Kabikinase<br>Durakinase	Distributors	European Pharmacopoeia 2002	API content , impurity<br><br>*UV-Spectrophotometry*	18/20 (90.0%)

**Reference / country of collection**	**Active pharmaceutical ingredient (API)**	**Brand name**	**Outlet**	**Reference standard**	**Test(s) performed and analytical technique for API content/Impurities**	**Failure frequency n/N (%)**
		Streptokinase<br>Eskinase<br>Icikinase<br>Thrombosolv<br>Strek<br>Solustrep<br>Streptonase<br>Icikinase<br>Heberkinase<br>STPase				
Vetchy, 2007 [12]<br>Czech Republic	Felodipine	Plenil®<br>Presid®	Unknown	European Pharmacopoeia 4.0<br>Czech Pharmacopoeia 2002<br>USP 26	Dissolution test<br><br>*UV spectrophotometry*	4/6 (66.7%)
R. Panchagnula, 2007 [13]<br>India	Nifedipine	Adalat retard OROS<br>Calbloc retard<br>Calcigard retard<br>Cardules retard<br>Depicor SR<br>Depin retard<br>Nicardia retard<br>Nifedine SR<br>Nifelet retard	Pharmacies	Unspecified	API Content, Weight uniformity, Hardness, Friability, Disintegration, Dissolution<br><br>*UV/Vis spectrophotometry*	0/15 (0.0%)
Veronin, 2008 [14]<br>Canada, United Kingdom, India, Mexico, Spain, Thailand, Fiji	Simvastatin	Zocor<br>Apo-Simvastatin<br>Co-Simvastatin<br>Novo-Simvastatin<br>Pms-Simvastatin<br>Simlo-20<br>SIMLIP-20<br>Starstat<br>Simi-20<br>Zorced<br>Simvastatina<br>Simastin 20	Website	USP 2003	API content, API identification, Appearance, Content uniformity, Disintegration, Dissolution, Hardness, Mass uniformity<br><br>*HPLC -DAD*	7/19 (36.8%)

Reference / country of collection	Active pharmaceutical ingredient (API)	Brand name	Outlet	Reference standard	Test(s) performed and analytical technique for API content/Impurities	Failure frequency n/N (%)
		Bestatin 20<br>Simlup-20				
Dione M Lima, 2008 [15]<br>Brazil	Enalapril	Unknown	Unknown	USP	API content and Dissolution<br><br>*HPLC-UV*	2 /9(22.2%)
Angeli, 2009 [16]<br>Thailand, Hungary, Czech Republic, Panama, South Africa, Belgium, Brazil, India, Pakistan, Dominican Republic, China	Ramipril	Generic	Unknown	European Pharmacopeia 2007<br>Sanofi-Aventis specification<br>USP	API content, Dissolution, Stability, Impurities<br><br>*HPLC (unknown detector)*	16/21 (76.2%)
De Braekeleer, 2009 [17] Belgium	Nifedipine	Adalat<br>Adalat Oros<br>Adalat Retard<br>Docnifedipine<br>Hypan<br>Nifedipine<br>Ratiopharm	Unknown	BP 2009<br>USP 2009<br>European Pharmacopeia 2005	API content,Related Substances,Dissolution,Content Uniformity<br><br>*HPLC-UV*	0/ 9 (0.0%)
Okoye, 2009 [18]<br>Nigeria	Nifedipine	Generic	Unknown	BP<br>USP 2002	API content, Disintegration, Dissolution, Friability, Hardness, Thickness, Weight, Diameter<br><br>*UV/Vis spectrophotometry*	0/7 (0.0%)
Benkovic , 2010 [19]<br>Croatia	Lisinopril	Amicor<br>Irumed<br>Laaven<br>Lizinopril-Farmal<br>Lizinopril Lek<br>Optimon<br>Skopryl<br>Vitopril<br>Lisinolex	Unknown	European Pharmacopeia 2007	Impurities<br><br>*HPLC (unknown detector)*	0/9 (0.0%)
Bunhak, 2010 [20]<br>Brazil	Acetylsalicylic acid	Generic	Basic pharmacies	USP 23,<br>BP 1999,	API content, Content uniformity, Disintegration, Dissolution,	6/ 8 (75.0%)

Reference / country of collection	Active pharmaceutical ingredient (API)	Brand name	Outlet	Reference standard	Test(s) performed and analytical technique for API content/Impurities	Failure frequency n/N (%)
				Brazilian Pharmacopeia	Friability, Hardness, Mass uniformity<br><br>*Titrimetry*<br>*UV-Spectrophotometry (dissolution)*	
Patel, P, 2010 [21] India	Telmisartan	Unknown	Unknown	USP	API Content, Weight uniformity, Hardness, Friability, Disintegration, Dissolution<br><br>*UV/Vis spectrophotometry*	0/12 (0.0%)
Mus'ab M. Khalaf, 2010 [22] Iraq	Furosemide	Apix<br>Desal<br>Lasimex<br>Salex	Private pharmacies	BP, USP	API content, Disintegration, Dissolution, Hardness, Weight uniformity<br><br>*Unstated technique*	0/5 (0.0%)
Eichie, 2011 [23] Nigeria	Amlodipine	Amlovar<br>Amlovasc<br>Swivasc<br>Lofra<br>Novasc<br>Amlovasc<br>Amlodipine<br>Asomex<br>Amlosam	Pharmacies	BP 2003	API content, Disintegration, Friability, Hardness, Weight uniformity, Dissolution<br><br>*UV/Vis spectrophotometry*	3 /10 (33.3%)
Walde 2011 [24] India	Acetylsalicylic acid	Apisafe<br>Caresprin.<br>Ecosporin<br>Nusprin<br>Sprintas	Private pharmacies	Indian Pharmacopeia<br>USP	API content, Content uniformity, Disintegration, Dissolution, Friability, Hardness, Mass uniformity<br><br>*Spectrophotometry*	1/6 (16.7%)
Olajide, 2011 [25] Nigeria	Amlodipine	Norvasc<br>Amlovasc<br>Amlovar<br>Amlong-10<br>Fepilat<br>Cardiosave-10	Retail pharmacies	USP 2000<br>Chinese Pharmacopeia 2005	Visual inspection, API Identification, Weight uniformity, Hardness, Friability, Disintegration, Dissolution, API content<br><br>*UV/Vis spectrophotometry*	4/6 (66.7%)
Oyeniyi, 2011 [26] Nigeria	Amlodipine	Generic	Manufacturer	BP 1998 +<br>BP 2010	API content, Disintegration, Dissolution, Friability, Hardness	0/5 (0.0%)

**Reference / country of collection**	**Active pharmaceutical ingredient (API)**	**Brand name**	**Outlet**	**Reference standard**	**Test(s) performed and analytical technique for API content/Impurities**	**Failure frequency n/N (%)**
					UV/Vis-Spectrophotometry	
Jinat Ashrafi, 2011 [27] Bangladesh	Atenolol-Amlodipine	Camlodin® Amlovas®	Pharmacy shops	BP USP	API content, Content uniformity, Disintegration, Dissolution, Friability, Hardness, Mass uniformity *UV/Vis-Spectrophotometry*	1/4 (25.0%)
T Shams Oishi, 2011[28] Bangladesh	Atorvastatin	Unknown	Local drug stores	USP	API content, Disintegration, Dissolution, Friability, Hardness, Thickness, Weight, Diameter *HPLC-UV*	6/10 (60.0%)
MNA Ameri, 2011 [29] , unstated country	Simvastatin Nifedipine	Generic	Unknown	BP USP European pharmacopeia	Dissolution *UV-Vis spectrophotometry*	0/3 (0.0%)
Akinleye M O , 2011 [30] Nigeria	Amlodipine	Generic	Pharmacies	BP	API Content, Weight uniformity, Hardness, Friability, Disintegration, Dissolution *UV/Vis spectrophotometry*	0/2 (0.0%)
Osadebe P.O, 2011 [31] Nigeria	Lisinopril	Generic	Local pharmacies	USP 22 BP 2001 European pharmacopeia	API content, hardness, friability, weight uniformity, disintegration time and dissolution *UV/Vis spectrophotometry*	3/3 (100.0%)
Oyetunde, 2012 [32] Nigeria	Propranolol	Generic	Manufacturer locally Wholesaler	BP 2007	Dissolution, API content *UV/Vis spectrophotometry*	0/2 (0.0%)
Giri, 2012 [33] India	Diltiazem	Dilzem CD 90 Angizem CD 90 Channel CD 90	Retail pharmacies	USP 2000 BP	API content, dissolution, disintegration and weight variation *UV-spectrophotometry*	0/3 (0.0%)
Walenga, 2012 [34] USA	Enoxaparin	Lovenox	Pharmacy and other hospital systems	Unspecified	API content *Anti-Factor Xa assay*	0 /10 (0.0%)

**Reference / country of collection**	**Active pharmaceutical ingredient (API)**	**Brand name**	**Outlet**	**Reference standard**	**Test(s) performed and analytical technique for API content/Impurities**	**Failure frequency n/N (%)**
Akinleye, 2012 [35] Nigeria	Atorvastatin	Generic	Registered pharmacy	USP 2004<br>BP 2007	API content, Content uniformity, Disintegration, Dissolution, Friability, Hardness, Mass uniformity<br><br>*HPLC-UV*	1/3 (33.3%)
Oyeniyi YJ, 2012 [36]<br>Nigeria	Nifedipine	Unknown	Pharmacies	USP<br>BP 2004	API content, Content uniformity, Disintegration, Dissolution, Friability, Hardness, Mass uniformity<br><br>*UV/Vis spectrophotometry*	6/10 (60.0%)
M A Noman, 2012 [37]<br>Yemen	Captopril	Unknown	Retail pharmacies	BP 2007	API Content, Weight uniformity, Hardness, Friability, Disintegration, Dissolution<br><br>*HPLC-UV spectrophotometry*	9/15 (60.0%)
Sani Ali. Audu, 2012 [38] Nigeria	Lisinopril	Lisiofil<br>Lisoril<br>Lizopril<br>Makinga<br>Ranopril<br>Stripril<br>Takapril<br>Teva<br>Zestril	Unknown	USP	API Content<br><br>*HPLC-UV*	4/9 (44.4%)
Chandra Dinda S, 2012 [39] India	Losartan	Generic	Unknown	Unknown	API Content<br><br>*UV/Vis spectrophotometry*	0/6 (0.0%)
S.O. Olayemi, 2012 [40] Nigeria	Amlodipine	Generic	Pharmacies	BP, USP	API Content, Weight uniformity, Hardness, Friability, Disintegration, Dissolution<br><br>*HPLC-UV-PDA*	4/8 (50.0%)
Haruna, 2013 [41] Nigeria	Methyldopa	Unknown	'Pharmaceutical shops'	USP 2000	API content<br><br>*Titrimetry*	1/4 (25.0%)

**Reference / country of collection**	**Active pharmaceutical ingredient (API)**	**Brand name**	**Outlet**	**Reference standard**	**Test(s) performed and analytical technique for API content/Impurities**	**Failure frequency n/N (%)**
Lichanda,B , 2013 [42] China	Telmisartan	Generic	Unknown	USP	API content, Dissolution, Impurity<br><br>*HPLC-UV*	6 /10(60.0%)
M Antonio de Oliveira, 2014 [43] Brazil	Hydrochlorothiazide	Generic	Compounding pharmacies	Brazilian pharmacopoeia 2010	API Content, Weight uniformity, Hardness, Friability, Disintegration, Dissolution<br><br>*UV-Spectrophotometry*	1/3 (33.3%)
Poonguzhali, 2014 [44] India	Atenolol	Tenolol<br>Ziblok<br>Itel<br>Atecard<br>Tenormin	Unknown	Indian Pharmacopeia	API content, Disintegration, Friability, Hardness, Weight uniformity, Dissolution,<br><br>*HPLC, colorimetry, spectrophotometry*	0/5 (0.0%)
Sylvester O. Eraga, 2014 [45] Nigeria	Amlodipine	Amlovar<br>Junvasc<br>Amlovasc<br>Amlodipine<br>Novasc<br>Amlodipine C-T<br>Lofra<br>Amlocard Swivasc – 5<br>Hyercla	Pharmacies	BP 2007<br>USP 2003	API content, Disintegration, Dissolution, Friability, Hardness, Thickness, Weight, Diameter<br><br>*UV Spectrophotometry*	2/10 (20.0%)
Senthil Rajan, 2014 [46] Malaysia	Atenolol	Generic	Retail pharmacies	BP<br>USP	Visual inspection, API Identification, Weight uniformity, Hardness, Friability, Disintegration, Dissolution, API content<br><br>*UV/Vis spectrophotometry*	0/5 (0.0%)
Safila Naveed, 2014 [47] Pakistan	Atenolol	Tenormin<br>Blokium<br>Atenolol<br>Cardiolyte	Unknown	BP<br>USP	Uniformity of weight, friability, hardness, disintegration, dissolution<br><br>*UV/Vis spectrophotometry*	0/4 (0.0%)
Safila Naveed, 2014 [48] Pakistan	Lisinopril	Zestril<br>Trupril<br>Novatec<br>Lame 5	Unknown	BP<br>USP	Uniformity of weight, friability, hardness, disintegration, dissolution<br><br>*UV/Vis spectrophotometry*	0/4 (0.0%)

**Reference / country of collection**	**Active pharmaceutical ingredient (API)**	**Brand name**	**Outlet**	**Reference standard**	**Test(s) performed and analytical technique for API content/Impurities**	**Failure frequency n/N (%)**
Huma Dilshad, 2014[49]<br>Pakistan	Captopril	Capoten<br>Capril<br>Acetopril<br>Capace	Unknown	BP<br>USP	Uniformity of weight, friability, hardness, disintegration, dissolution<br><br>*UV/Vis spectrophotometry*	0/4 (0.0%)
Mahwish Feroz, 2014 [50]<br>Pakistan	Amlodipine	Unknown	Retail pharmacies	USP 2006<br>European Pharmacopeia 2007	Weight variation, hardness, thickness, length, breadth, friability, disintegration, dissolution and assay<br><br>*UV spectrophotometry*	0/6 (0.0%)
Tapas K Pal, 2014 [51] India	Telmisartan	Unknown	Pharmacies	USP	API content, API identification, Disintegration, Friability, Hardness, Mass uniformity, Visual inspection<br><br>*UV/Vis spectrophotometry*	2 /6(33.3%)
Han,Y, 2014 [52] Argentina	Furosemide	Generic	Pharmacies	USP	Dissolution<br><br>*UV/Vis spectrophotometry*	0/8 (0.0%)
Gamil Q. Othman, 2014 [53] Yemen	Lisinopril	Cipril<br>Lisistril<br>Lotensin<br>zenoril<br>Zestril	Pharmacies	BP, USP	API Content, Weight uniformity, Hardness, Friability, Disintegration, Dissolution<br><br>*HPLC-UV*	0/5 (0.0%)
Rehana Begum, 2014 [54] Bangladesh	Losartan	Generic	Local drug store	USP	Dissolution, Hardness, Friability, Disintegration	0/3 (0.0%)
Sharifa Sultana, 2014 [55] Bangladesh	Furosemide	Generic	Unknown	BP, USP	API content, Disintegration, Dissolution, Friability, Hardness, Thickness, Weight, Diameter, Appearance<br><br>*UV/Vis spectrophotometry*	1/6 (16.7%)
Basha, 2015 [56]<br>Albania	Olmesartan	Unknown	Pharmacies	USP 2009	API content, Disintegration, Dissolution, Friability, Hardness, Thickness, Weight, Diameter<br><br>*HPLC-DAD*	0/4 (0.0%)
Abdelkarim, 2015 [57]<br>Sudan	Atenolol	Unknown	Unknown	USP<br>BP 2013	API content, Disintegration, Dissolution, Friability, Hardness, Thickness, Weight, Diameter	0 /3(0.0%)

**Reference / country of collection**	**Active pharmaceutical ingredient (API)**	**Brand name**	**Outlet**	**Reference standard**	**Test(s) performed and analytical technique for API content/Impurities**	**Failure frequency n/N (%)**
					UV/Vis spectrophotometry	
Siaan, 2015 [58] Libya	Furosemide	Duresan® *Generic* Furosémide	'Local market' (not detailed)	Unspecified	API content, API identification, Appearance, Content uniformity, Disintegration, Dissolution, Hardness, Mass uniformity<br><br>*HPLC-UV/Vis*	0/5 (0.0%)
Zhang, 2015 [59] China	Nifedipine	Unknown	Local pharmacies	USP 34 2011	Impurity<br><br>*HPLC-UV*	0/14 (0.0%)
Osei-Asare, 2015 [60] Ghana	Nifedipine	*Generic*	Community and hospital pharmacies	BP 2011 USP 2007	API content, Weight uniformity, Dissolution Test<br><br>*HPLC-UV*	6/12 (50.0%)
G O. Okpanachi, 2015 [61] Nigeria	Methyldopa	Unknown	Unknown	BP	API content, Disintegration, Dissolution, Friability, Hardness, Thickness, Weight, Diameter<br><br>*UV- Spectrophotometry*	6/8 (75.0%)
Ogochukwu , 2015 [62] Nigeria	Valsartan	Unknown	Registered wholesale	USP	API Content, Weight uniformity, Hardness, Friability, Disintegration, Dissolution<br><br>*HPLC- UV*	0 /4(0.0%)
T Locatelli, 2015 [63] Brazil	Propranolol-Hydrochlorothiazide	*Generic*	Pharmacies	Brazilian pharmacopoeia	API Content, Weight uniformity, Hardness, Friability, Disintegration, Dissolution<br><br>*UV/Vis spectrophotometry*	1/6 (16.7%)
Ming-Yuan Zhang, 2015 [64] China	Nifedipine	*Generic*	Unknown	USP	Impurity, Weight<br><br>*HPLC-UV*	0/14 (0.0%)
Md. Abir Khan, 2015 [65] Bangladesh	Atorvastatin	*Generic*	Local pharmacies	USP	API Content, Weight uniformity, Hardness, Friability, Disintegration, Dissolution	2/5 (40.0%)

**Reference / country of collection**	**Active pharmaceutical ingredient (API)**	**Brand name**	**Outlet**	**Reference standard**	**Test(s) performed and analytical technique for API content/Impurities**	**Failure frequency n/N (%)**
					UV/Vis spectrophotometry	
Shawahna, 2016 [66] Palestine	Atorvastatin	*Generic*	Local retail pharmacies	USP 2005	API content, Hardness<br><br>*HPLC (unknown detector)*	0/4 (0.0%)
Mansour, 2016 [67] Syrian Arab Republic	Furosemide	Unknown	Registered pharmacies	BP<br>USP	API Content, Weight uniformity, Hardness, Friability, Disintegration, Dissolution<br><br>*Spectrophotometry*	1/3 (33.3%)
Karmoker , 2016 [68] Bangladesh	Amlodipine	*Generic*	Local drug store	Unspecified	API content, Content uniformity, Disintegration, Dissolution, Friability, Hardness, Mass uniformity<br><br>*UV/Vis spectrophotometry*	3/4 (50.0%)
Alnuhait, MA , 2016 [69]<br>Saudi Arabia	Atenolol	Tenormin<br>Tenolol<br>Tenol<br>Atormin<br>Apo-Atenol<br>Hypoten<br>Cardol	Retail pharmacies	BP 2013	API content, Disintegration, Dissolution, Friability, Hardness, Thickness, Weight, Diameter<br><br>*UV spectrophotometry*	0/7 (0.0%)
Elghnimi,TY, 2016 [70]<br>Libya	Simvastatin	Simvastatin®<br>Simvastatin-1APharma®<br>Vascor®<br>Simvastatina Normon®	Pharmacies	BP<br>USP	Visual inspection, API Identification, Weight uniformity, Hardness, Friability, Disintegration, Dissolution, API content,<br><br>*HPLC*	0/4 (0.0%)
Hameed A, 2016 [71] Pakistan	Losartan	*Generic*	Unknown	BP	API content, Disintegration, Dissolution, Friability, Hardness, Thickness, Weight, Diameter<br><br>*Unstated technique*	0/2 (0.0%)
Tapas K Pal, 2016 [72] India	Rosuvastatin	Rozucor<br>Rozumac<br>Rosuvas<br>Zyrova<br>Crestor	Unknown	USP<br>Indian pharmacopeia	API Content, Weight uniformity, Hardness, Friability, Disintegration, Dissolution<br><br>*UV/Vis spectrophotometry*	2/5 (40.0%)

**Reference / country of collection**	**Active pharmaceutical ingredient (API)**	**Brand name**	**Outlet**	**Reference standard**	**Test(s) performed and analytical technique for API content/Impurities**	**Failure frequency n/N (%)**
Nesar, 2017 [73] Pakistan	Atenolol	*Generic*	Retail pharmacies	USP	API content, Disintegration, Dissolution, Friability, Hardness, Thickness, Weight, Diameter<br><br>*UV/Vis spectrophotometry*<br>*HPLC*	2/6 (33.3%)
Wajiha, 2017 [74] Pakistan	Atorvastatin	*Generic*	Registered pharmacies	USP 2011<br>BP	API content, Disintegration, Friability, Hardness, Weight uniformity, Dissolution<br><br>*HPLC-UV*	0/7 (0.0%)
Orfao, 2017 [75] Brazil	Hydrochlorothiazide	*Generic*	Unknown	Brazilian pharmacopoeia	API content, Content uniformity, Disintegration, Dissolution, Friability, Hardness, Mass uniformity<br><br>*UV/VIS-spectrophotometry*<br>*FTIR*	1/5 (20.0%)
Mohamed M. Siaan, 2017 [76] Libya	Atenolol	*Generic*	Manufacturer	USP<br>BP	Visual inspection, API Identification, Weight uniformity, Hardness, Friability, Disintegration, Dissolution, Uniformity dosage unit, API content,<br><br>*UV/Vis spectrophotometry*	0/3 (0.0%)
Mustafa Beesh, 2017 [77]<br>Syrian Arab Republic	Atenolol	*Generic*	Pharmacies	BP 2009<br>USP 2013	API content, Content uniformity, Disintegration, Dissolution, Friability, Hardness, Mass uniformity<br><br>*UV- Spectrophotometry*	3/4 (75.0%)
A Ali Bassam, 2017 [78]<br>Yemen	Clopidogrel	*Generic*	Pharmacies	USP	API content, Content uniformity, Disintegration, Dissolution, Friability, Hardness, Mass uniformity<br><br>*UV- Spectrophotometry*	6 /8(75.0%)

**Reference / country of collection**	**Active pharmaceutical ingredient (API)**	**Brand name**	**Outlet**	**Reference standard**	**Test(s) performed and analytical technique for API content/Impurities**	**Failure frequency n/N (%)**
C. N. Chinaka , 2017 [79]<br>Nigeria	Furosemide	Unknown	Pharmacies	BP 2012<br>USP 2009	API Content, Weight uniformity, Hardness, Friability, Disintegration, Dissolution, Packaging<br><br>*UV/Vis spectrophotometry*	2/5 (40.0%)
Lovely Z, 2017 [80]<br>Bangladesh	Clopidogrel	Unknown	Retail pharmacies	USP 2013	API Content, Weight uniformity, Hardness, Friability, Disintegration, Dissolution<br><br>*UV spectrophotometry*	3 /9(33.3%)
M Isbera, 2017 [81]<br>Syrian Arab Republic	Warfarin	Unknown	Registered pharmacies	USP	API content, Content uniformity, Disintegration, Dissolution, Friability, Hardness, Mass uniformity<br><br>*UV-spectrophotometry*	1/3 (33.3%)
F Hasin, 2017 [82]<br>Bangladesh	Valsartan	Unknown	Pharmacies	USP	API Content, Weight uniformity, Hardness, Friability, Disintegration, Dissolution<br><br>*HPLC (unknown detector)*	0/3 (0.0%)
T Tasnim, 2017 [83]<br>Bangladesh	Olmesartan	Unknown	Retail pharmacies	BP 2013<br>USP	API content, Disintegration, Dissolution, Friability, Hardness, Thickness, Weight, Diameter<br><br>*UV Spectrophotometry*	0/9 (0.0%)
H Jung-Cook, 2017 [84]<br>Mexico	Amlodipine	*Generic*	Unknown	USP	Dissolution<br><br>*UV/VIS spectrophotometry*	0/3 (0.0%)
Promit Das, 2018 [85]<br>Bangladesh	Spironolactone	*Generic*	Pharmacies	USP	API content, Disintegration, Dissolution, Friability, Hardness, Thickness, Weight, Diameter<br><br>*UV/Vis spectrophotometry*	0/6 (0.0%)
Aivalli, 2018 [86]<br>India	Atenolol<br>Amlodipine	Unknown	Government and private pharmacies	Indian Pharmacopeia 2014	API content, API Identification, Dissolution, Impurity, Weight uniformity	0/10 (0.0%)

**Reference / country of collection**	**Active pharmaceutical ingredient (API)**	**Brand name**	**Outlet**	**Reference standard**	**Test(s) performed and analytical technique for API content/Impurities**	**Failure frequency n/N (%)**
					HPLC, infrared spectroscopy	
Agune, 2018 [87]<br>Ethiopia	Nifedipine	*Generic*	Retail outlets	USP 2013	Weight Uniformity, Hardness, Disintegration, Dissolution<br><br>*HPLC (unknown detector)*	2/6 (33.3%)
Mehnaz Ali, 2018 [88]<br>Bangladesh	Valsartan	Unknown	Local drug store	USP	API content, Disintegration, Dissolution, Friability, Hardness, Thickness, Weight, Diameter<br><br>*UV/Vis spectrophotometry*	1/3 (33.3%)
M Rahamathulla, 2018 [89]<br>Saudi Arabia	Atorvastatin	Lipitortm<br>Lorvast<br>Lipomax<br>Astatin	Community pharmacies	USP	API content, Content uniformity, Disintegration, Dissolution, Friability, Hardness, Mass uniformity<br><br>*UV/Vis spectrophotometry*	0/4 (0.0%)
Adefolarin A Amu, 2018 [90]<br>Swaziland	Nifedipine	Unknown	Retail pharmacies and central medical stores	USP	Weight variation, diameter, hardness, friability, drug content and disintegration<br><br>*UV spectrophotometry*	4/4 (100.0%)
M. A. Varillas, 2018 [91]<br>Argentina	Hydrochlorothiazide	Unknown	Pharmacies	Argentine Pharmacopeia	API Content, Weight uniformity, Hardness, Friability, Disintegration, Dissolution<br><br>*UV- spectrophotometry*	1/4 (25.0%)
Marques de Andrade, 2018 [92]<br>Brazil	Verapamil	*Generic*	Pharmacies	Brazilian pharmacopoeia	API Content, Weight uniformity, Hardness, Friability, Disintegration, Dissolution<br><br>*UV/Vis spectrophotometry*	0/4 (0.0%)
R F Naddour, 2018 [93]<br>Syrian Arab Republic	Warfarin	*Generic*	Unknown	USP<br>BP	API Content, Weight uniformity, Hardness, Friability, Disintegration, Dissolution<br><br>*UV- VIS spectrophotometry*	0/8 (0.0%)

**Reference / country of collection**	**Active pharmaceutical ingredient (API)**	**Brand name**	**Outlet**	**Reference standard**	**Test(s) performed and analytical technique for API content/Impurities**	**Failure frequency n/N (%)**
M Gayathri Devi, 2018 [94] India	Propranolol	Pronol<br>Propochem<br>Inderal<br>Ciplar	Unknown	Indian pharmacopeia<br>USP	API Content, Weight uniformity, Hardness, Friability, Disintegration, Dissolution<br><br>*UV/Vis spectrophotometry*	0 /4(0.0%)
Manal AL-Yosofy, 2018 [95] Sudan	Amlodipine	*Generic*	Registered Pharmacies	USP	API content, Disintegration, Dissolution, Friability, Hardness, Thickness, Weight, Diameter<br><br>*UV/Vis spectrophotometry*	0/2 (0.0%)
Pabitra Thapa, 2018 [96] Nepal	Amlodipine	*Generic*	Unknown	Indian Pharmacopeia	API content, Disintegration, Dissolution, Hardness, Weight uniformity<br><br>*HPLC-UV*	0/11 (0.0%)
M Kumar, 2018 [97] India	Enalapril	*Generic*	Unknown	Indian Pharmacopeia	API Content, Weight uniformity, Hardness, Friability, Disintegration, Dissolution<br><br>*UV/Vis spectrophotometry*	0/2 (0.0%)
Sanja Kecman, 2018 [98]	Amlodipine	*Generic*	Unknown	European Pharmacopoeia	API Content, Weight uniformity, Hardness, Friability, Disintegration, Dissolution<br><br>*HPLC-UV-PDA*	0/2 (0.0%)
A Bayoumi, 2019 [99] Iraq	Irbesartan	Aprovel<br>Converium<br>Gizlan	Unknown	USP	API Content, Weight uniformity, Hardness, Friability, Disintegration, Dissolution<br><br>*UV spectrophotometry*	1/3 (33.3%)
J S Bayasa, 2019 [100] India	Telmisartan	Unknown	Retail pharmacies	USP<br>Indian pharmacopeia	API Content, Weight uniformity, Hardness, Friability, Disintegration, Dissolution<br><br>*UV/Vis spectrophotometry*	0/4 (0.0%)
Marta I.V. Brevedan, 2019 [101] Argentina	Furosemide	*Generic*	Unknown	Argentine Pharmacopeia, USP	API Content, Weight, Hardness, Friability, Disintegration, Dissolution, Packaging	0/7 (0.0%)

**Reference / country of collection**	**Active pharmaceutical ingredient (API)**	**Brand name**	**Outlet**	**Reference standard**	**Test(s) performed and analytical technique for API content/Impurities**	**Failure frequency n/N (%)**
					UV spectrophotometry	
Arnold C. I, 2019 [102] Nigeria	Lisinopril	Unknown	Pharmacies	BP, USP	API Content, Weight uniformity, Hardness, Friability, Disintegration, Dissolution<br><br>*UV/Vis spectrophotometry*	9/10 (90.0%)
Rehana Begum, 2019 [103] Bangladesh	Atenolol	*Generic*	Unknown	BP + USP	Dissolution, Hardness, Friability, Disintegration	0/3 (0.0%)
A S Zaek, 2019 [104] Libya	Metoprolol<br>Verapamil<br>Spironolactone	Aldactone<br>Apo metoprolol<br>Isoptin<br>Lachipres	Unknown	BP	API Content, Thickness, Hardness and Disintegration time<br><br>*UV/Vis spectrophotometry*	0/6 (0.0%)
K.S. Salako, 2019 [105] Nigeria	Methyldopa	*Generic*	Pharmacies	BP , USP	API content, Disintegration, Dissolution, Friability, Hardness, Thickness, Weight, Diameter, Appearance<br><br>*HPLC-UV*	6/10 (60.0%)
Elias Sakkal, 2019 [106] Syrian Arab Republic	Acetylsalicylic acid<br>Captopril<br>Furosemide	Asiapirin<br>Aspirex<br>Capotal<br>Capoten<br>Captophen<br>Furosemid<br>*Generic*<br>Obarsix<br>Rasperine	Pharmacies	USP	API content<br><br>*HPLC-DAD - MS/MS*	0/10 (0.0%)
Maryana S, 2019 [107] Syrian Arab Republic	Amlodipine	Unknown	Manufacturers	USP	API content, Content uniformity, Disintegration, Dissolution, Friability, Hardness, Weight uniformity<br><br>*HPLC-UV*	3/5 (60.0%)
Bilawal Shaikh, 2020 [108]Pakistan	Captopril	Unknown	Local pharmacies	USP	API content, Disintegration, Dissolution, Friability, Hardness, Thickness, Weight, Diameter, Appearance	2/6 (33.3%)

**Reference / country of collection**	**Active pharmaceutical ingredient (API)**	**Brand name**	**Outlet**	**Reference standard**	**Test(s) performed and analytical technique for API content/Impurities**	**Failure frequency n/N (%)**
					HPLC-UV	
Arnold C. I, 2020 [109] Nigeria	Amlodipine	Unknown	Pharmacies	BP, USP	API Content, Weight uniformity, Hardness, Friability, Disintegration, Dissolution<br><br>*UV spectrophotometry*	3/10 (30.0%)
Akasha, A, 2020 [110] Libya	Amlodipine	Amady<br>Amlodipine<br>Amlor	Unknown	USP	Weight uniformity, Hardness, Friability, Disintegration, Dissolution, Visual inspection<br><br>*Infrared (IR) spectroscopy*	5/5 (100.0%)
D Basava Raju, 2020 [111] India	Losartan	Cosart<br>Losakind<br>Losar	Retail pharmacies	USP	API content, Dissolution, Friability, Hardness, Thickness, Weight<br><br>*UV/Vis spectrophotometry*	0/3 (0/0%)
Silvia Farfan, 2020 [112] Argentina	Clopidogrel	*Generic*	Pharmacies	USP	Appearance, Dissolution, Weight variation	0/8 (0.0%)
Varghese George, 2020 [113] India	Clopidogrel	Unknown	Unknown	Indian Pharmacopeia	API Content<br><br>*HPLC-UV*	8/11 (72.7%)
I Haque, 2020 [114] India	Atenolol	A-AtenololIP<br>Acord Plus<br>HIPRES-50	Private pharmacies	Indian Pharmacopeia	API content, Disintegration, Dissolution, Friability, Hardness, Thickness, Weight<br><br>*UV/Vis spectrophotometry*	0/3 (0.0%)
Mosab Arafat, 2020 [115] United Arab Emirates	Furosemide	Lasix<br>Furosemide	Pharmacies	USP , European Pharmacopeia	Comparative compositional analysis (vs authentic or other brands), Weight uniformity, Hardness, Friability, Disintegration, Dissolution<br><br>*Fourier Transform Infrared Spectrometry (FTIR)*	0/2 (0.0%)
Dalia Gaber, 2020 [116] Saudi Arabia	Atenolol	*Generic*	Pharmacies	USP	API content, Disintegration, Dissolution, Hardness, Weight uniformity	0/4 (0.0%)

**Reference / country of collection**	**Active pharmaceutical ingredient (API)**	**Brand name**	**Outlet**	**Reference standard**	**Test(s) performed and analytical technique for API content/Impurities**	**Failure frequency n/N (%)**
					UV/Vis spectrophotometry	

HPLC, High-performance liquid chromatography; MS, Mass spectrometry; UV, Ultraviolet; TLC, Thin Layer Chromatography; FTIR, Fourier transformed infrared spectroscopy; LC, Liquid Chromatography; DAD, Diode-Array Detection; PDA, Photo diode array; BP, British Pharmacopeia; USP, United Stated Pharmacopeia; API, Active Pharmaceutical Ingredient

1 Akbuğa J, Gürsoy A. Studies on Furosemide Tablets I Dissolutions of Commercial Products and Different Formulations. *Drug Dev Ind Pharm* 1987;**13**:2199–208. doi:10.3109/03639048709020580

2 Sakolkhai S, Chaiyakum A, Aromdee C, *et al.* A Survey on Qualities of Drugs Commercially Available in Thailand. Published Online First: 2010.https://www.researchgate.net/publication/277206970_A_Survey_on_Qualities_of_Drugs_Commercially_Avilable_in_Thailand

3 Nikolić L, Djurić Z, Jovanović M, *et al.* In Vitro Evaluation of Commercial Aspirin Tablets Marketed in Yugoslavia. *Drug Dev Ind Pharm* 1994;**20**:75–83. doi:10.3109/03639049409047215

4 Baloglu E. Quality control studies on enalapril maleate tablets available on the turkish drug market. *Ankara Univ Eczac Fak Derg* 2001;**30**:1–16. doi:DOI: 10.1501/Eczfak_0000000351

5 Mayet C. L, Jung-Cook H, Mendoza A. O, *et al.* A Comparative Study on the Dissolution Profiles of Commercial Hydrochlorothiazide Tablets. *Rev Mex Ciencias Farm* 2008;**39**:18–24.

6 Gomez Y, Adams E. Analysis of purity in 19 drug product tablets containing clopidogrel: 18 copies versus the original brand. *J Pharm Biomed Anal* 2004;**34**:341–8. doi:10.1016/S0731-7085(03)00533-8

7 Petersen K-U. In vitro Release of Felodipine from Original Brand and Generic Products. *Arzneimittelforschung* 2011;**53**:40–3. doi:10.1055/s-0031-1297068

8 Longstaff C, Thelwell C, Whitton C. The poor quality of streptokinase products in use in developing countries. *J Thromb Haemost* 2005;**3**:1092–3. doi:10.1111/j.1538-7836.2005.01271.x

9 Dires T. A comparative in vitro evaluation of anti hypertensive drugs products (methyldopa, furosemide and propranolol tablets) from local market, Addis Ababa. 2005. http://etd.aau.edu.et/bitstream/handle/123456789/4895/Teshome Dires.pdf?sequence=1&isAllowed=y

10 Smith J., Tarocco G, Merazzi F, *et al.* Are generic formulations of carvedilol of inferior pharmaceutical quality compared with the branded formulation? *Curr Med Res Opin* 2006;**22**:709–20. doi:http://dx.doi.org/10.1185/030079906X96461

11 Hermentin P, Cuesta-Linker T, Weisse J, *et al.* Comparative analysis of the activity and content of different streptokinase preparations. doi:10.1093/eurheartj/ehi219

12 Vetchy D, Vetcha M, Rabiškova M, *et al.* Comparison in vitro felodipine release rate from the original versus generic product with controlled release of the drug. *Medicina (B Aires)* 2007;**43**:326. doi:10.3390/medicina43040040

13 Panchagnula R, Singh R, Ashokraj Y. In vitro evaluation of modified release formulations of nifedipine from Indian market. *Indian J Pharm Sci*

2007;**69**:556. doi:10.4103/0250-474X.36944

14 Veronin MA, Nguyen NT. Comparison of Simvastatin Tablets from the US and International Markets Obtained via the Internet. *Ann Pharmacother* 2008;**42**:613–20. doi:10.1345/aph.1K560

15 Lima DM, dos Santos LD, Lima EM. Stability and in vitro release profile of enalapril maleate from different commercially available tablets: Possible therapeutic implications. *J Pharm Biomed Anal* 2008;**47**:934–7. doi:10.1016/j.jpba.2008.02.030

16 Angeli DG, Trezza C. Quality and stability of ramipril generics/copies versus reference ramipril (Tritace): A 3-month stability comparative study. *Clin Drug Investig* 2009;**29**:667–76. doi:http://dx.doi.org/10.2165/11315270-000000000-00000

17 De Braekeleer K, Fierens C. Nifedipine preparations on the Belgian Market: A comparative study. *J Pharm Belg* 2009;**64**:141–6.http://ovidsp.ovid.com/ovidweb.cgi?T=JS&PAGE=reference&D=emed11&NEWS=N&AN=358176356

18 Okoye, E I, Iwuagwu, *et al.* Physicochemical equivalence of some brands of Nifedipine retard tablets available in Nigeria. *African J Biotechnol* 2010;**9**:1274–9. doi:10.5897/AJB09.1466

19 Benkovic G, Sokolic M, Cudina B, *et al.* Analysis of purity profiles of generic lisinopril tablets marketed in Croatia. *Coll Antropol* 2013;**37**:601–6.http://hrcak.srce.hr/file/153953

20 Bunhak E., Stoef P. Physicochemical quality evaluation of acetylsalicylic acid tablets distributed by a basic pharmacy. *Lat Am J Pharm* 2010;**29**:1358–63.http://www.latamjpharm.org/trabajos/29/8/LAJOP_29_8_1_13_VZQSYP16SD.pdf

21 Patel PA. Commercial Telmisartan Tablets: A Comparative Evaluation with Innovator Brand Micardis. 2010. http://www.ijpsr.info/docs/IJPSR10-01-08-04.pdf (accessed 28 May 2019).

22 Khalaf ab M, Oglah MK, Amar TA. Chemical and biopharmaceutical assay of different brands of frusemide tablets. Mosul University 2011. doi:10.33899/IPHR.2011.49573

23 Eichie F., Arhewoh M., Isesele J. In vitro assessment of quality control parameters of some commercially available generics of amlodipine besylate in Nigerian drug market. *Int J Heal Res* 2011;**4**:57–61. doi:http://dx.doi.org/10.4314/ijhr.v4i1.70437

24 Walde S., Rasala T., Gurunani G. Marketed preparations: Quality evaluation of delayed release aspirin tablet. *Int Res J Pharm* 2011;**2**:90–2.http://www.irjponline.com/admin/php/uploads/vol2/issue4/15.pdf

25 Olajide AS, Chidinnma OC, Dennis UE. Comparative Assessment Of The Quality Control Measurements Of Multisource Amlodipine Tablets Marketed In Nigeria. *Int J Biomed Adv Res* 2011;**1**. doi:10.7439/ijbar.v1i4.10

26 Oyeniyi YJ, Acho M. A Comparative Pharmaceutical Evaluation Of Amlodipine (5mg)Tablets Registered In Nigeria By Nafdac, And Marketed By Various Pharmaceutical Companies Nationwide. https://innovareacademics.in/journal/ijpps/Vol3Suppl5/2863.pdf

27 Ashrafi J. Evaluation of the Quality Control Parameters of Two Different Brands of Combined Atenolol (50mg) & Amlodipine (5mg) Tablets Available in Bangladesh. http://dspace.ewubd.edu/bitstream/handle/123456789/1601/Jinat_Ashrafi .pdf?sequence=1&isAllowed=y (accessed 27 May 2019).

28 Oishi T, Nimmi I, Islam S. Comparative in vitro Bioequivalence Analysis of Some Generic Tablets of Atorvastatin, a BCS Class II Compound. *Bangladesh Pharm J* 2011;**14**:61–6.http://bps-bd.org/journal/volume14/11.pdf

29 Al Ameri MN, Nayuni N, Anil Kumar KG, *et al.* The differences between the branded and generic medicines using solid dosage forms: In-vitro dissolution testing. *Results Pharma Sci* 2012;**2**:1–8. doi:10.1016/j.rinphs.2011.12.001

30 Akinleye MO, Oyetunde OO, Okpara HE, *et al.* Equivalence Of Two Generic Brands Of Amlodipine Besylate Under Biowaiver Conditions. https://innovareacademics.in/journal/ijpps/Vol4Issue2/3358.pdf (accessed 27 May 2019).

31 Osadebe P., Uzor P., Enwereji P. Quality Control and Interchangeability of MultisourcedLisinopril Tablets Marketed in Nigeria. 2011.https://www.semanticscholar.org/paper/Quality-Control-and-Interchangeability-of-Tablets-P.O-Uzor/f2a752fccf3b950431658699385d69faad78c6ec (accessed 24 Sep 2019).

32 Olubukola OO, Fola T, Moshood OA, *et al.* In Vitro Equivalence Studies of Generic Metformin Hydrochloride Tablets and Propranolol Hydrochloride Tablets Under Biowaiver Conditions in Lagos State, Nigeria. *Dissolution Technol* Published Online First: 2012.http://dissolutiontech.com/DTresour/201211Articles/DT201211_A07.pdf

33 Giri T., Tandan H., Choudhary C, *et al.* In-vitro evalution of commercially available sustained release capsule containing Diltiazem hydrochloride. *Int J Pharm Pharm Sci* 2012;**4**:523–6.http://www.ijppsjournal.com/Vol4Issue3/4311.pdf

34 Walenga J, Jeske W, Hoppensteadt D, *et al.* Comparative studies on branded enoxaparin and a US generic version of enoxaparin. *Clin Appl Thromb* 2013;**19**:261–7. doi:http://dx.doi.org/10.1177/1076029612463427

35 Akinleye MO, Idris O, Nwachukwu PN, *et al.* Quality of brands of atorvastatin calcium tablets marketed in Lagos , Nigeria. *Int J Pharm Pharmacol* 2012;**1**:1–7.

36 Oyeniyi YJ, AYORINDE J. Pharmaceutical Evaluation Of Some Commercial Brands Of Nifedipine (20mg) Sustained Release Tablets, Marketed In Commercial City Of Kano. 2012. http://ijbpas.com/pdf/1336904153MS IJBPAS 2012 1091.pdf

37 Noman MA, Albooryhi M, Sayf AA. In-vitro evaluation of Captopril tablets present in Yemen markets. 2019;:10–2.

38 Audu SA, Taiwo AE, Waziri FI. Comparative Evaluation Study on Different Brands of Lisinopril Tablet using HPLC and UV Spectrophotometer. J. Nat. Sci. Res. 2012.https://iiste.org/Journals/index.php/JNSR/article/view/2761/2785

39 Chandra Dinda S. A comparative study of three brands of losartan potassium tablets by UV Spectrophotometry. 2012.https://www.researchgate.net/publication/305378597_A_comparative_study_of_three_brands_of_losartan_potassium_tablets_by_UV_Spectrophotometry

40 Olayemi S, Akinleye M., Awodele E., *et al.* The physicochemical equivalence of eight brands of amlodipine tablets in Lagos, Nigeria. West Afr. J. Med. https://www.researchgate.net/publication/234106441_The_physicochemical_equivalence_of_eight_brands_of_amlodipine_tablets_in_Lagos_Nigeria

41 Haruna B, Adaku O. Chemical equivalence study on four brands of alpha-methyldopa. *J Chem Pharm Res* 2013;**5**:356–8.http://jocpr.com/vol5-iss10-2013/JCPR-2013-5-10-356-358.pdf

42 Lichanda B, Luo M, Wang H, *et al.* A comparative evaluation of the quality of ten generic telmisartan tablets with the brand. 2013;**7**:2043–53. doi:10.5897/AJPP12.1251

43 De Oliveira MA, Yoshida MI, Monteiro Da Silva DCG. Quality evaluation of pharmaceutical formulations containing hydrochlorothiazide. *Molecules* 2014;**19**:16824–36. doi:10.3390/molecules191016824

44 Poonguzhali S, Anusha K, Mounica T, *et al.* Comparative invitro evaluation of commercial atenolol tablets. *Res J Pharm Biol Chem Sci* 2014;**5**:30–5.http://www.rjpbcs.com/pdf/2014_5(6)/[4].pdf

45 Eraga SO, C.Uzochukwu O, Iwuagwu MA. Pharmaceutical Equivalence of Some Brands of 5 mg Amlodipine Besylate Tablets Available in Southern Nigeria. 2019;**36**:1–5. doi:10.1088/0256-307X/36/3/030302

46 Senthil Rajan D, Azizi M, Shanmugham S, *et al.* Comparative Quality Control Evaluation of Atenolol Tablets Marketed in Kuala Lumpur, Malaysia. 2014. www.sciencedomain.org (accessed 27 May 2019).

47 Safila N, Dilshad H. Comparitive Study Of Different Brands Of Atenolol Available In Karachi. *Mod Chem Appl* 2014;**02**:4–7.

48 Safila N, Dilshad H, Waheed N. Comparative Study of Four Different Brands of Lisinopril Available In Karachi. *Int Res J Pharm* 2014;**5**:374–7.

49 Dilshad H, Naveed S, Waheed N. Comparative Study Of Four Different Brands Of Captopril Available In Karachi, Pakistan. *Int Res J Pharm* 2014;**5**:374–7. doi:10.7897/2230-8407.050578

50 Feroz MM, Razvi N, Ghayas S, *et al.* Assessment Of Pharmaceutical Quality Control And Equivalence Of Various Brands Of Amlodipine Besylate (5 Mg) Tablets Available In The Pakistani Market Under Biowaiver Conditions. *undefined* Published Online First: 2014.https://www.semanticscholar.org/paper/ASSESSMENT-OF-PHARMACEUTICAL-QUALITY-CONTROL-AND-OF-Feroz-Razvi/889bb178a3017b8a1b9b2b5b192e6f05727e6729

51 Pal TK, Ghosh U, Panda M. Comparative Bioequivalence Study Of Different Brands Of Telmisartan Tablets Marketed In India By Dissolution Modeling And Quality Control Tests. *Int J Pharm Phytopharm Res*;**6084**.

52 Han YK, Laura Simionato D, Calvo RG, *et al.* Comparison of Dissolution Profiles Available in the Argentinian Market of Furosemide Tablets. Published Online First: 2016. doi:10.6000/1927-5951.2016.06.01.1

53 Comparative Analysis of Five Brands of Lisinopril Tablets in Yemeni Market. https://www.researchgate.net/publication/304627984_Comparative_Analysis_of_Five_Brands_of_Lisinopril_Tablets_in_Yemeni_Market (accessed 18 Nov 2020).

54 Begum R, Sultan2 MZ, Rahman A, *et al.* The In Vitro Pharmaceutical Equivalence Studies Of Losartan Tablets Of Different Manufacturers Available In Bangladesh. https://1library.net/document/download/qorxkn5q#

55 Comparative quality evaluation of different brands of Frusemide tablets available in Bangladeshi market. https://www.researchgate.net/publication/324274495_Comparative_quality_evaluation_of_different_brands_of_Frusemide_tablets_available_in_Bangladeshi_market (accessed 18 Nov 2020).

56 Basha B, Malaj L. A comparative study on the quality of different olmesartan tablets available in the albanian market. *Asian J Pharm Clin Res* 2016;**9**:80–3.http://innovareacademics.in/journals/index.php/ajpcr/article/download/9505/4034

57 Abdelkarim A, Mohammed A, Abdeen M. Assessment of pharmaceutical quality control and in vitro equivalence of various brands of atenolol (100MG) tablets available in sudanese market under biowaiver conditions. *Int J Pharm Sci Res* 2015;**6**:2390–4. doi:http://dx.doi.org/10.13040/IJPSR.0975-8232.6%286%29.2390-94

58 Siaan MM, Altketik KA, Almarzouki A, *et al.* Evaluation of the Pharmaceutical Quality of Some Furosemide Tablet Brands. 2015;**4**:238–46.

59 Zhang M-Y, Zhang J-D, Gao Q, *et al.* Evaluation Procedure for Quality Consistency of Generic Nifedipine Extended-Release Tablets Based on the Impurity Profile. *Am J Anal Chem* 2015;**06**:776–85. doi:10.4236/ajac.2015.69074

60 Osei-Asare C, Kipo SL, Ofori-Kwakye K, *et al.* Comparative in vitro dissolution of commercially available sustained release nifedipine tablet brands in the Kumasi Metropolis, Ghana. *J Appl Pharm Sci* 2015;**5**:54–060. doi:10.7324/JAPS.2015.50809

61 Okpanachi GO, Musa H, Mohammed Y, *et al.* Assessment Of Physicochemical Properties Of Eight Brands Of Alpha Methyldopa Tablets Marketed In Zaria , Kaduna State, Nigeria. 2019.

62 Ogochukwu A, Olubukola O, Moshood A, *et al.* Biowaiver assessment of some commercially available brands of valsartan tablets using. 2015;**26**:1–11.

63 Locatelli T, da Silva AK, Sausen TR. Pharmaceutical equivalence of anti-hypertensive tablets distributed by a Brazilian governmental program. *Lat Am J Pharm* 2015;**34**:1601–6.

64 Zhang MY, Zhang JD, Gao Q, *et al.* Evaluation Procedure for Quality Consistency of Generic Nifedipine Extended-Release Tablets Based on the Impurity Profile. *scirp.org*https://www.scirp.org/html/6-2201212_59270.htm

65 Comparative in vitro pharmaceutical equivalence studies of different brands of atorvastatin calcium tablets marketed in Bangladesh. https://1library.net/document/download/zkk4k48z (accessed 18 Nov 2020).

66 Shawahna R, Hroub AK, Abed E, *et al.* Pharmaceutical quality of generic atorvastatin products compared with the innovator product: A need for revising pricing policy in Palestine. *Acta Pol Pharm - Drug Res* 2016;**73**:725–30.http://www.ptfarm.pl/pub/File/Acta_Poloniae/2016/nr 3/725.pdf

67 Mansour O, Ismail G, Mostafaisbera M, *et al.* Assessment of physicochemical properties of furosemide (40mg) tablets marketed in Syria. *J Chem Pharm Sci* 2016;**9**:2879–81.

68 Karmoker J, Joydhar P, Sarkar S RM. Comparative in vitro evaluation of various commercial brands of amlodipine besylate tablets marketed in Bangladesh. *Asian J Pharm Heal Sci* 2016;**6**:1384–9. doi:http://www.ajphs.com/journals/02_AJPHS_Jan-Mar_2016_738.pdf

69 Alnuhait MA, Guard N, Affairs H, *et al.* In-vitro evaluation of some generic products of atenolol commonly sold in the Saudi market. 2016.

70 Elghnimi TY, El-Majri MA, Bennouh R, *et al.* Comparative In-Vitro Evaluation Of Commercial Simvastatin Tablets (20 Mg). Int. J. Pharm. Sci. Res. 2016.http://ijpsr.com/bft-article/comparative-in-vitro-evaluation-of-commercial-simvastatin-tablets-20-mg/?view=fulltext

71 A H, S N, SS A, *et al.* Pharmaceutical Equivalent Study of Losartan Potassium Formulation available in Karachi, Pakistan. *J Bioequiv Availab* 2016;**08**. doi:10.4172/jbb.1000310

72 Pal T, Saha D, Maity S. Bioequivalence Modulation With Modified Starch In Orodispersible Tablets In Comparison To Marketed Conventional Tablets Of Rosuvastatin Calcium Bioequivalence Modulation With Modified Starch In Orodispersible Tablets In Comparison To Marketed Conventional. *Eur J Pharm Med Res* · 2016.

73 Nesar S, Rahim N, Yousuf RI, *et al.* Evaluation of pharmaceutical quality and bio waiver study of atenolol tablets available in Karachi (Pakistan). *Lat Am J Pharm* 2017;**36**:2007–11.http://www.latamjpharm.org/trabajos/36/10/LAJOP_36_10_1_18_NE96561QEP.pdf

74 Iffat W, Shoaib MH, Rahim N, *et al.* Assessment of Pharmaceutical Equivalence of Different Generic Atorvastatin Tablets available in Pakistan. *Lat Am J Pharm* 2017;**36**:2098–104.http://www.latamjpharm.org/trabajos/36/10/LAJOP_36_10_1_31_2LG5V0ZWN5.pdf

75 Órfão MK, Alves JO, Valladão AS, *et al.* Quality Control Of Hydrochlorothiazide Tablets Marketed In Sinop-Mt. *Sci Electron Arch* 2017;**10**.https://www.ingentaconnect.com/content/doaj/23169281/2017/00000010/00000005/art00016

76 Siaan MM, Anwair MAS, Sadek F, *et al.* Comparative in Vitro Evaluations of Different Brands of Atenolol Tablets in Libyan Market. *J Chem Biol Phys Sci* 2018;**7**:857–67. doi:10.24214/jcbps.a.7.4.85767

77 Mustafa B, Isbera M, Aboud K, *et al.* Quality Analysis Of Different Marketed Brands Of Atenolol (50 Mg) Available In Syria And Comparing With The Reference Drug. *World J Pharm Res* 2014;**3**:1961–7. doi:10.20959/wjpr201714-9929

78 Ali BA, Al-haddad MG, Areqi AA. Comparative Evaluation of Some Commercial Clopidogrel Tablets Available in Yemen. *Maj Farm* 2018;**13**:79–87.https://jurnal.ugm.ac.id/majalahfarmaseutik/article/view/40917/22797 (accessed 27 May 2019).

79 Chinaka CN, Nwachukwu N. Comparative In Vitro Quality Assessment of Five Brands of Furosemide Tablets Marketed in Port Harcourt , Nigeria. 2017.

80 Abdul A, Sanjida H, Ik S, *et al.* Comparative In Vitro Quality Evaluation of Some Clopidogrel Tablets, Commercially Available in Bangladesh Drug Market. https://medwinpublishers.com/BEBA/BEBA16000117.pdf (accessed 28 May 2019).

81 Isbera M, Aboud K, Haroun M. Quality control of Warfarin Sodium tablets marketed in Syria. *Res J Pharm Technol* 2017;**10**:2119. doi:10.5958/0974-360X.2017.00371.7

82 Hasin F. Comparative Bioequivalence Study of Different Brands of Valsartan Tablets Marketed in Bangladesh By Dissolution Modeling and Quality Control Tests. *World J Pharm Pharm Sci* 2017;:112–21. doi:10.20959/wjpps20177-9562

83 Tasnim T, Hasan. M. Comparative Quality Evaluation of Different Olmesartan Medoxomil Generic Tablets Marketed in Bangladesh. *Int J Adv Res* 2017;**5**:1403–10. doi:10.21474/ijar01/4852

84 Jung-Cook H, Mayet-Cruz L, Girard-Cuesy ME. Comparative in vitro dissolution and in vivo bioavailability of commercial amlodipine tablets. *Trop J Pharm Res* 2018;**17**:1685. doi:10.4314/tjpr.v17i9.1

85 Promit Das, Das S, Akhter R, *et al.* Comparative In Vitro Equivalence Evaluation Of Some Spironolactone Generic Tablets, Commercially Available In Bangladesh Drug Market. *Indo Am J Pharm Res* Published Online First: 2017.https://www.academia.edu/35017673/COMPARATIVE_IN_VITRO_EQUIVALENCE_EVALUATION_OF_SOME_SPIRONOLACTONE_GENERIC_TABLETS_COMMERCIALLY_AVAILABLE_IN_BANGLADESH_DRUG_MARKET (accessed 24 Sep 2019).

86 Aivalli PK, Elias MA, Pati MK, *et al.* Perceptions of the quality of generic medicines: implications for trust in public services within the local health system in Tumkur, India. *BMJ Glob Heal* 2017;**2**:e000644. doi:10.1136/bmjgh-2017-000644

87 Agune G, Nigatu M, Gabriel T, *et al.* Comparative In Vitro Evaluation Of Different Brands Of Nifedipine 20mg Retard Tablet Products Marketed In Addis Ababa, Ethiopia. *J Drug Deliv Ther* 2018;**8**:1–5. doi:10.22270/jddt.v8i3.1685

88 Ali M, Faizah Ali F, Akhter Rita N, *et al.* Comparative in vitro evaluation of some commercial brands of valsartan tablets marketed in Bangladesh. *~ 1068 ~ Pharma Innov J* 2018;**7**:1068–72.www.thepharmajournal.com (accessed 27 May 2019).

89 Rahamathulla M. Comparative assessment of different marketed brands of atorvastatin tablets in Asir region, Saudi Arabia. Published Online First: 2018. doi:10.15406/mojddt.2018.02.00023

90 Amu AA, Masondo B, Lukhele A, *et al.* Assessment of Pharmaceutical Equivalence and In-Vitro Bioequivalence of Four Brands of Commercially Available Nifedipine Tablets in Swaziland. *Mod Appl Bioequiv Availab* 2018;**4**. doi:10.19080/MABB.2018.04.555626

91 Varillas MA, Brevedan MIV, Vidal NLG. Pharmaceutical equivalence of hydrochlorothiazide tablets in Argentina. *Dissolution Technol* 2018;**25**:22–7. doi:10.14227/DT250418P22

92 Marques de Andrade G, Carvalho Ribeiro C, Castro Alves Plank B, *et al.* Pharmaceutical equivalence and comparative dissolution profile studies for coated tablets containing Verapamil hydrochloride. *J Appl Pharm Sci* 2018;**5**:57–71.

93 Naddour RF, Kaddar NA. In vitro bioequivalence for three products of warfarin sodium in syrian market. *Res J Pharm Technol* 2018;**11**:532–6. doi:10.5958/0974-360X.2018.00099.9

94 Devi MG, Savithri M, Devi U, *et al.* Comparitive In-Vitro Evaluation Of Commercially Available Generic And Branded Propranolol Hydrochloride Immediate Release Tablets. *Int J Pharm DRUG Anal* 2018;**6**:29–34.

95 Al-Yosofy M, Al-Domini Y. Assessment Of Pharmaceutical Quality Control And Bioequivalence Of Various Brands Of Amlodipine Besylate 10 Mg Tablets Marketed In Sudan Under Biowaiver Conditions. *www.wjpps.com* 2018;**7**:118. doi:10.20959/wjpps20189-12213

96 Thapa P, Manandhar N, Pandeya S. In-Vitro Evaluation of Amlodipine Tablets Available in Nepalese Market. *J Nepal Chem Soc* 2018;**39**:46–52. doi:10.3126/jncs.v39i0.27027

97 Kumar M, Tyagi S, Bhatt S, *et al.* Comparative Evaluation of Two Different Marketed Brands of Enalapril maleate. *J Drug Deliv Ther* 2018;**8**:265–8. doi:10.22270/jddt.v8i6-s.2127

98 Kecman S, Goločorbin-Kon S, Mikov M. Physicochemical Equivalence Studies of Two Amlodipine Tablet Formulations. *Scr Med (Brno)* 2018;**49**:18–24. doi:10.18575/msrs.sm.e.18.03

99 Bayoumi AA. Quality assessment of selected marketed irbesartan 300mg tablets brands in Iraq. 2019;:10–3. doi:10.13140/RG.2.2.19781.50401

100 Bayas JS, Cheke RS, Lokhande P, *et al.* The In-vitro studies and evaluation of telmisartan marketed tablets. *J Drug Deliv Ther* 2019;**9**:74–8. doi:10.22270/jddt.v9i1.2267

101 Brevedan MIV, Varillas MA. Pharmaceutical equivalence and stability of furosemide tablets in Argentina. *Dissolution Technol* 2019;**26**:30–7. doi:http://dx.doi.org/10.14227/DT260419P30

102 Igboasoiyi AC, Egeolu AP, Amos BG. Quality Assessment of Various Brands of Lisinopril Tablets sold in Uyo Metropolis using UV Spectrophotometry. 2019. www.nijophasr.com

103 Begum R, Sultan MZ, Chowdhury JA, *et al.* In vitro Pharmaceutical Equivalence Study of Three Brands of Atenolol Tablets Available in Bangladesh. *Dhaka Univ J Pharm Sci* 2019;**18**:43–8. doi:10.3329/dujps.v18i1.41426

104 Zaek AS, Benhamed A, Al MA, *et al.* Comparative study of pharmaceutical content of three different cardiovascular system drugs marketed in Tripoli-Libya. 2019. www.pcbiochemres.com

105 Salako KS, Ayininuola O, Badejo R., *et al.* Quantitative assay of ten brands of methyldopa tablets marketed in Lagos, Nigeria. 2019. doi:DOI:10.4314/JOPHAS.V16I1

106 Sakkal E, Bitar Y, And ST-RJ of P, *et al.* Quality control of the active Pharmaceutical ingredients of some Pharmaceutical products prior the termination of their shelf life. *indianjournals.com*http://www.indianjournals.com/ijor.aspx?target=ijor:rjpt&volume=12&issue=12&article=085

107 Salamah M, Alahmad Y. Assessment and comparison of pharmaceutical equivalence of amlodipine besylate tablets available in Syria under biowaiver conditions. *Res J Pharm Technol* 2020;**13**:1720–4. doi:10.5958/0974-360X.2020.00310.8

108 Shaikh B, Ghoto MA, Arain MI, *et al.* Comparative In-vitro Evaluation of Different Captopril Tablet Brands Commercially Available in Sindh, Pakistan. *J Pharm Res Int* 2020;**32**:131–6. doi:10.9734/jpri/2020/v32i2130763

109 Igboasoiyi AC, Egeolu AP, Edet EM. Quality evaluation and UV spectrophotometric assay of ten brands of amlodipine tablets marketed in Uyo, Nigeria. *Journal* 2020;**17**:60–5. doi:10.4314/jpb.v17i1.10

110 Akasha A. A Comparative Study for Evaluation of Five Formulations Of Amlodipine 10 mg Tablets Marketed in Libya. *undefined* Published Online First: 2020.http://innoriginal.com/index.php/iijs/article/view/229

111 Evaluation of Different Marketed Brands of Losartan Potassium Tablets: A Comparative Study with Generic Shop Products. https://www.researchgate.net/publication/344758402_Evaluation_of_Different_Marketed_Brands_of_Losartan_Potassium_Tablets_A_Comparative_Study_with_Generic_Shop_Products_Corresponding_Author (accessed 18 Nov 2020).

112 Silvia F, Marina Marcos V, Octavio F, *et al.* Comparative dissolution and polymorphism study of clopidogrel bisulfate tablets available in Argentine. *J Appl Pharm Sci* Published Online First: October 2020. doi:10.7324/japs.2020.10107

113 George V, Mullavelil K, Joseph AT, *et al.* Quantitative analysis, cost comparison, physician, and patient perceptions on generic versus branded clopidogrel bisulfate tablets marketed in Kerala, India. *j-pcs.org*http://www.j-pcs.org/article.asp?issn=2395-5414;year=2020;volume=6;issue=1;spage=40;epage=46;aulast=George

114 Haque I, Ghosh S, Joshi H, *et al.* Comparison and Evaluation of Freely Supplied Government and Ethically Marketed Antihypertensive Drug (Atenolol). *Drug Des Intellect Prop Int J* Published Online First: 2020. doi:10.32474/DDIPIJ.2020.03.000164

115 Arafat M, Fahelelbom K, Sarfraz M, *et al.* Comparison between branded and generic furosemide 40 mg tablets using thermal gravimetric analysis and Fourier transform infrared spectroscopy. *J Pharm Bioallied Sci* 2020;**12**:489. doi:10.4103/jpbs.jpbs_365_19

116 Gaber D, Abdoun S, Maswadeh H, *et al.* Equivalence study of immediate release tablets of beta blocker drug. *Int J Appl Pharm* 2020;**12**:53–9. doi:10.22159/ijap.2020v12i2.36477
