# Supplementary material for: The quality of medical products for cardiovascular diseases: a gap in global cardiac care
Source: BMJ Glob Health. 2021 Sep 14;6(9):e006523. doi: 10.1136/bmjgh-2021-006523 (PMC8442059; doi:10.1136/bmjgh-2021-006523)
Supplement: Supplementary data [file bmjgh-2021-006523supp011.pdf]

Supplementary file 11. Detailed results for the equivalence studies (n=116) of cardiovascular medicines

We found 116 equivalence studies from 1987 to 2020, 83.6% (97/116) were found in the last 10 years (2010-2020).

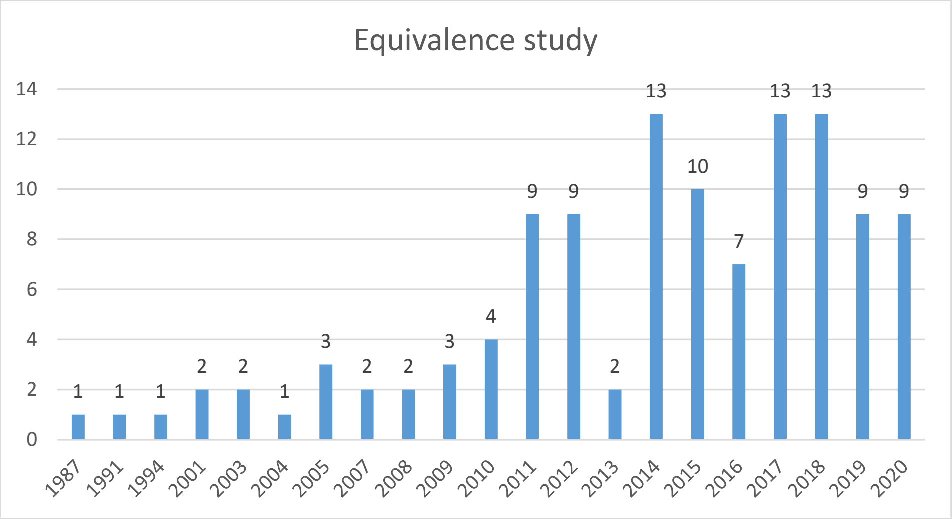

Most samples included in equivalence studies were generics (72.3%, 594/822) with a FF of 31.6% (188/594). Innovator brands had a FF of 16.0% (4/25). No information on whether the samples were generics or innovators were reported for 203 samples tested.

Failure frequency by a version of drugs in equivalence studies

Because of the limited number of samples tested for quality in the studies included in this review, the figures should not be interpreted as representative of the prevalence of specific SF cardiovascular medicines (please refer to the discussion section of the current paper for more details)
Failure frequency is defined as the proportion of samples that failed at least one quality test

Version of Drug	Failure Frequency % (n/N)
**Generic**	31.6% (188/594)
**Innovator**	16.0% (4/25)
**Unknown**	38.9% (79/203)
**Total**	**33.0% (271/822)**

In the equivalence studies, most of the samples were tested for more than one quality attribute (88.4 %, 727/822). 692 samples (692/822, 84.2%) were tested for API content, 143 for impurity/contaminant (143/822, 17.4%), and dissolution test was performed on 660 samples (660/822, 80.3%). Packaging analysis was conducted for 17.9% (n=147) of 822 samples.

The failure frequency due to impurity/contaminant (34/146, 23.3%) was the highest, followed by API content (124/692, 17.9%), and dissolution (104/660, 15.8%) (**Table below**). None of the 147 samples for which packaging analysis was performed failed packaging analysis in the equivalence studies.

Of 125 samples that failed the API content tests, 71.2% (n=89) contained a lower API amount than stated on the packaging, 18.4% (n=23) higher API, and one (0.8%) sample did not contain the stated API. For 9.6% (n=12) of the samples, there were not enough details in the publication as to whether they contained higher or lower amounts of API or no API.

Failure frequency by quality test in equivalence studies

Because of the limited number of samples tested for quality in the studies included in this review, the figures should not be interpreted as representative of the prevalence of specific SF cardiovascular medicines (please refer to the discussion section of the current paper for more details)

Quality test	Failure Frequency % (n/N)
**Impurity/Contaminant/Related substance**	23.1% (34/147)
**API content**	18.0% (125/694)
**Dissolution**	15.7% (104/662)
**Uniformity of units** *	2.5% (14/557)
**Packaging**	0.0% (0/147)
**Other chemical analysis****	0.0% (0/26)
**Other physical analysis*****	5.7% (93/1,622)

*** Includes: content uniformity, weight uniformity, uniformity of mass, weight variation**
**** Includes: bioavailability, identify APIs, Excipients composition, Spectral comparison (vs authentic or other brands)**
*****Includes: friability, hardness, disintegration**

Note: ***One sample may have been tested for one or more quality tests***
