# Supplementary material for: The quality of medical products for cardiovascular diseases: a gap in global cardiac care
Source: BMJ Glob Health. 2021 Sep 14;6(9):e006523. doi: 10.1136/bmjgh-2021-006523 (PMC8442059; doi:10.1136/bmjgh-2021-006523)
Supplement: Supplementary data [file bmjgh-2021-006523supp012.pdf]

**Supplementary file 12: Recalls and alerts of poor quality sartans due to impurities in 2018, 2019 and 2020**

| Continent | Country | API | Number of products-Manufacturers | No. Batches | Month & Year | Ref |
|---|---|---|---|---|---|---|
| **Africa** | Nigeria | Valsartan | 7 products – Dexcel Pharm Limited<br>Actavis Group PTC EHF | - | Aug 2018 | [1] |
| | Ghana | Valsartan | Denk Pharma, Germany | - | Dec 2018 | [2] |
| | | Valsartan | 2 products from Actavis, United Kingdom,<br>2 products from Torrent Pharmaceuticals Ltd, India | All | Sep 2018 | [3] |
| | South Africa | Valsartan | 3 product -Pharma Dynamic company | All | Jul 2018 | [4] |
| **Americas** | United States | Valsartan | 8 products from AvKARE, Inc. (Hetero/Camber), Solco Healthcare LLC. (Prinston | All | 2019 | [5] |
| | | Valsartan products | Many companies | 698 | 2019 | [5] |
| | | Losartan products | Many companies | 445 | 2019 | [5] |
| | | Irbesartan ,<br>Telmisartan,<br>Olmesatan products | Many companies | 45<br>65<br>7 | 2019 | [5]<br>[6] |
| | | Valsartan products | Torrent Pharmaceuticals Limited<br>Teva Pharmaceutical USA<br>Aurobindo Pharma Limited | 119<br>80<br>22 | 2020 | [7]<br>[8]<br>[9] |
| | | Valsartan products | Camber Pharmaceuticals, Inc | All | 2020 | [10] |
| | Canada | Valsartan<br>Losartan<br>Irbesartan | Many companies | 16<br>53<br>1 | Jul2018<br>Nov2018 | [11]<br>[12]<br>[13] |
| **Asia** | India | Losartan,<br>Valsartan,<br>Telmisartan | Many companies | 7 | Aug2013<br>Nov2018 | [14] |
| | Thailand | Valsartan | 2 products –Silom Medical<br>3 products- Unison Laboratories | All | 2018 | [15] |
| | | Losartan | 2 product-Berlin Pharmaceutical Industry Company (Lanzaar 50 and 100) | 142 | Mar2019 | [16] |
| | Vietnam | Valsartan | 32 products from Vietnam's companies and 25 imported products | All | Aug 2018 | [17] |
| | Hong-Kong | Valsartan | 5 products- Actavis and HK Medical companies | - | July 2018 | [18] |
| **Europe** | Denmark | Valsartan | 2Care4 Medical Ltd | 6 | Jul2018 | [19] |
| | | Valsartan-HCTZ | Orifarm Group A/SStada Arzneimittel AG | 20 | Jul2018 | [19] |
| | France | Irbesartan | Cristers Laboratoire<br>Mylan Pharmaceuticals<br>Zydus Cadila | 17 | 2019 | [20] |
| | | Irbesartan-HCTZ[a] | Arrow Génériques<br>Zydus Cadila | 10 | 2019 | [20] |
| | | Losartan | Accord Healthcare | 1 | 2019 | [20] |

| | | | | | | |
|---|---|---|---|---|---|---|
| | | Valsartan-ALP | Teva pharmaceutical, France | 59 | 2019 | [20] |
| | United Kingdom | Valsartan | 5 products-Mylan company<br>3 products-DEXCEL PHARMA LIMITED<br>4 products- ACTAVIS GROUP PTC EHF<br>Teva UK Limited | All batches | 2018 | [21]<br>[22] |
| | | Valsartan-HCTZ | 1 product- ACTAVIS GROUP PTC EHF<br>3 products-Teva UK Limited | All batches<br>21 batches | Nov2018 | [23] |
| | | Irbesartan | Actavis Group PTC EHF<br>Macleods Pharma UK Limited | 2 batches<br>3 batches | Jan-Feb 2019 | [24]<br>[25]<br>[26] |
| | | Irbesartan-HTCZ | Actavis Group PTC EHF | 5 batches | | |
| | | Losartan | Accord Healthcare Limited | 3 batches | Mar2019 | [27] |
| **Multicountry** | 24 countries * | Valsartan | Unstated | 2300 batches | Jul2018 | [28] |

** Germany, Norway, Finland, Sweden, Hungary, The Netherlands, Austria, Ireland, Bulgaria, Italy, Spain, Portugal, Belgium, France, Poland, Croatia, Lithuania, Greece, Canada, Bosnia and Herzegovina, Bahrain, and Malta*

## REFERENCES


1 Nigeria Government. Recall Of Valsartan Due To Contamination. *NAFDAC* Published Online First: 2018.https://www.nafdac.gov.ng/recall-of-valsartan-due-to-contamination/

2 Ghana FDA. Recall of Amlodipine/Valsartan combination tablets due to contamination with an impurity (N-Nitroso-Dimethylamine). *Ghana FDA* Published Online First: 2018.https://fdaghana.gov.gh/images/stories/pdfs/Dear Helthcare Prof/2018/18-398-recall of amlodipine valsartan comb tab-dhpl0001.pdf (accessed 25 Sep 2019).

3 Ghana Government. RECALLED PRODUCTS SINCE JANUARY 2018. *Ghana FDA* Published Online First: 2019.https://fdaghana.gov.gh/index.php/recalled-products-since-january-2018/

4 SAHPRA. Recommendations about the urgent Recall of Valsartan In Response to the Potential Risk of Cancer. *South African Heal Prod Regul Auth* Published Online First: 2018.http://www.health.gov.za/index.php/2014-03-17-09-48-36/2014-03-17-09-49-50?download=2853:press-release-recommendations-about-the-urgent-recall-of-valsartan-in-response-to-the-potential-risk-of-cancer

5 USFDA. FDA Updates and Press Announcements on Angiotensin II Receptor Blocker (ARB) Recalls (Valsartan, Losartan, and Irbesartan). 2019.https://www.fda.gov/drugs/drug-safety-and-availability/fda-updates-and-press-announcements-angiotensin-ii-receptor-blocker-arb-recalls-valsartan-losartan (accessed 4 Jun 2019).

6 Enforcement Report US Food and Drug Administration Recall Information Search. https://www.accessdata.fda.gov/scripts/ires/index.cfm#tabNav_advancedSearch (accessed 16 Sep 2019).

7 USFDA. Updated:additional lots added Torrent Pharmaceuticals Limited Issues Voluntary Nationwide Recall of Valsartan/Amlodipine/HCTZ, Valsartan/Amlodipine and Valsartan Tablets | FDA. 2020.https://www.fda.gov/safety/recalls-market-withdrawals-safety-alerts/updatedadditional-lots-added-torrent-pharmaceuticals-limited-issues-voluntary-nationwide-recall (accessed 12 Dec 2020).

8 USFDA. Teva Pharmaceuticals USA Issues Voluntary Nationwide Recall of Valsartan and Valsartan Hydrochlorothiazide Tablets | FDA. 2020.https://www.fda.gov/safety/recalls-market-withdrawals-safety-

alerts/teva-pharmaceuticals-usa-issues-voluntary-nationwide-recall-valsartan-and-valsartan (accessed 12 Dec 2020).

9 USFDA. Aurobindo Pharma Limited Issues Voluntary Recall of Irbesartan Drug Substance Due to the Detection of Trace Amounts of NDEA (NNitrosodiethylamine) Impurity Found in the Active Pharmaceutical Ingredient (API) | FDA. 2020.https://www.fda.gov/safety/recalls-market-withdrawals-safety-alerts/aurobindo-pharma-limited-issues-voluntary-recall-irbesartan-drug-substance-due-detection-trace (accessed 12 Dec 2020).

10 USFDA. Camber Pharmaceuticals, Inc. Issues Voluntary Nationwide Recall of Valsartan Tablets, USP, 40mg, 80mg, 160mg and 320mg Due to The Detection of Trace Amounts of N-Nitrosodimethylamine (NDMA) Impurity, Found in an Active Pharmaceutical Ingredient (API) | FD. 2020.https://www.fda.gov/safety/recalls-market-withdrawals-safety-alerts/camber-pharmaceuticals-inc-issues-voluntary-nationwide-recall-valsartan-tablets-usp-40mg-80mg-160mg (accessed 16 Dec 2020).

11 Canada Government. Information Update - Mylan-Valsartan medications voluntarily recalled as a precaution due to an impurity. Heal. Canada. 2018.https://www.newswire.ca/news-releases/information-update---mylan-valsartan-medications-voluntarily-recalled-as-a-precaution-due-to-an-impurity-701503141.html (accessed 16 Sep 2019).

12 Health Canada. Pro Doc Limitée voluntarily recalls two lots of irbesartan drugs because of nitrosamine impurity - Recalls and safety alerts. https://healthycanadians.gc.ca/recall-alert-rappel-avis/hc-sc/2019/69328a-eng.php (accessed 16 Sep 2019).

13 Canada Government. Multiple Losartan-containing drugs voluntarily recalled because of potential for nitrosamine impurity. *Heal Canada* Published Online First: 2018.http://www.healthycanadians.gc.ca/recall-alert-rappel-avis/hc-sc/2019/69272a-eng.php

14 Alerts. https://cdsco.gov.in/opencms/opencms/en/Notifications/Alerts/ (accessed 4 Sep 2019).

15 Thailand Government. Thailand is First Asian Country to " Recall Valsartan " Over Potential Links to Cancer. Published Online First: 2018.https://www.chiangraitimes.com/thailand-is-first-asian-country-to-recall-valsartan-over-potential-links-to-cancer.html

16 Thailand Government. FDA recalls Lanzaar 50 and Lanzaar 100 over cancer fears. *Nation Thail Portal* Published Online First: 2019.https://www.nationthailand.com/national/30365885

17 Vietnam Government. Vietnam recalls 57 heart drugs with cancer-causing substance. *Vnexpress* Published Online First: 2018.https://e.vnexpress.net/news/news/vietnam-recalls-57-heart-drugs-with-cancer-causing-substance-3787277.html

18 Hong Kong Goverment. Recall of five valsartan-containing pharmaceutical products. *Gov Hong Kong Spec Adm Reg* Published Online First: 2018.https://www.info.gov.hk/gia/general/201807/06/P2018070600841.htm

19 Denmark Government . Recall of valsartan blood pressure medicine. *Danish Med Agency* Published Online First: 2018.https://laegemiddelstyrelsen.dk/en/news/2018/recall-of-valsartan-blood-pressure-medicine

20 Rappel de lots de médicaments à base d'irbésartan - Point d'Information. ANSM. 2019.https://ansm.sante.fr/S-informer/Points-d-information-Points-d-information/Rappel-de-lots-de-medicaments-a-base-d-irbesartan-Point-d-Information (accessed 10 Jun 2019).

21 UK Government. Class 2 Medicines Recall: Teva UK Limited and Mylan - recall of some Valsartan containing products. GOV.UK. 2018.https://www.gov.uk/drug-device-alerts/class-2-medicines-recall-teva-uk-limited-and-mylan-recall-of-some-valsartan-containing-products (accessed 25 Sep 2019).

22 UK Government. Class 1 Medicines Recall: Action Now – including out of hours Pharmacy Level Recall.

GOV.UK. 2018.https://www.gov.uk/drug-device-alerts/class-1-medicines-recall-action-now-including-out-of-hours-pharmacy-level-recall (accessed 25 Sep 2019).

23 Class 1 Medicines Recall. https://www.gov.uk/drug-device-alerts/class-1-medicines-recall-action-now-including-out-of-hours-pharmacy-level-recall (accessed 4 Sep 2019).

24 UK Government. Class 2 Medicines Recall: Actavis Group PTC EHF - recall of batches of Irbesartan/Hydrochlorothiazide 300/12.5mg Film-coated Tablets and Irbesartan/Hydrochlorothiazide 150/12.5mg Film-coated Tablets - GOV.UK. Med. Healthc. Prod. Regul. Agency UK. 2019.https://www.gov.uk/drug-device-alerts/class-2-medicines-recall-actavis-group-ptc-ehf-recall-of-batches-of-irbesartan-hydrochlorothiazide-300-12-5mg-film-coated-tablets-and-irbesartan-hydrochlorothiazide-150-12-5mg-film-coated-tablets (accessed 25 Sep 2019).

25 UK Government. Class 2 Medicines recall: Irbesartan and Irbesartan / Hydrochlorothiazide. *Med Healthc Prod Regul Agency UK* Published Online First: 2019.https://assets.publishing.service.gov.uk/media/5c62f395e5274a317cc0ebc3/EL_19_A04-Final.pdf (accessed 25 Sep 2019).

26 UK Government. Class 2 Medicines Recall: Macleods Pharma UK Limited - Irbesartan 150mg Film-coated tablets, PL 34771/0079 (MDR 94-06/18). Med. Healthc. Prod. Regul. Agency UK. 2019.https://www.gov.uk/drug-device-alerts/class-2-medicines-recall-macleods-pharma-uk-limited-irbesartan-150mg-film-coated-tablets-pl-34771-0079-mdr-94-06-18 (accessed 25 Sep 2019).

27 UK Government. Class 2 Medicines recall: Accord Healthcare Limited - Losartan Potassium 50mg Film-coated Tablets, PL 20075/0022 and Losartan Potassium 100mg Film-coated Tablets, PL 20075/0023 - GOV.UK. Med. Healthc. Prod. Regul. Agency UK. 2019.https://www.gov.uk/drug-device-alerts/class-2-medicines-recall-accord-healthcare-limited-losartan-potassium-50mg-film-coated-tablets-pl-20075-0022-and-losartan-potassium-100mg-film-coated-tablets-pl-20075-0023 (accessed 25 Sep 2019).

28 Farrukh M, Tariq M, Malik O, *et al.* Valsartan recall: global regulatory overview and future challenges. *Ther Adv Drug Saf* 2019;**10**:204209861882345. doi:http://dx.doi.org/10.1177/2042098618823458
