# Supplementary material for: The quality of medical products for cardiovascular diseases: a gap in global cardiac care
Source: BMJ Glob Health. 2021 Sep 14;6(9):e006523. doi: 10.1136/bmjgh-2021-006523 (PMC8442059; doi:10.1136/bmjgh-2021-006523)
Supplement: Supplementary data [file bmjgh-2021-006523supp013.pdf]

Supplementary file 13: Recall/Alert/Case-report of substandard/falsified of cardiovascular medicines (except sartans, see Supplementary file 12)

Country	API/combination of APIs	Year	No. Batch	Quality test failure	Report Type	Ref
India	Acetylsalicylic acid	Aug-Nov 2013<br>Dec 2019	3<br>1	Dissolution<br>Dissolution	Recall/Alert	[1]
	Amlodipine	Dec 2013- May 2014<br>Feb 2020	4<br>1	API content/ Dissolution<br>Related Substances	Recall/Alert	[1]
	Atenolol-Amlodipine	Jan 2016	2	API content	Recall/Alert	[1]
	Atorvastatin	Nov 2016- Dec2016<br>May 2020	3<br>1	Dissolution<br>Dissolution	Recall/Alert	[1]
	Atorvastatin-Acetylsalicylic acid	May 2015	1	Dissolution	Recall/Alert	[1]
	Carvedilol	Jul 2017<br>Jul2020	1<br>1	Related substances<br>Assay	Recall/Alert	[1]
	Clopidogrel-Acetylsalicylic Acid	Jan 2019<br>Apr 2020	1<br>1	API content<br>Dissolution	Recall/Alert	[1]
	Enalapril	Sep 2017- Dec 2018<br>Apr 2020<br>Apr 2020	3<br>1<br>1	API content/ Dissolution<br>Dissolution/ Assay<br>Assay	Recall/Alert	[1]
	Furosemide	Dec 2016- Jun 2017	3	Dissolution	Recall/Alert	[1]
	Glyceryl Trinitrate	Dec 2016- Feb 2019	2	API content/impurity	Recall/Alert	[1]
	Heparin	Dec 2018- 2019	2	pH/ API content	Recall/Alert	[1]
	Isosorbide dinitrate	Jul 2013- Mar 2019	3	API content/ Dissolution	Recall/Alert	[1]
	Labetalol	Jul 2017	2	Visible particles	Recall/Alert	[1]
	Methyldopa	Jul 2017	1	API content	Recall/Alert	[1]
	Metoprolol	Nov 2017	1	Dissolution	Recall/Alert	[1]
	Nifedipine	Nov 2013- Jan 2014<br>May 2020	2<br>1	Dissolution<br>Dissolution	Recall/Alert	[1]
	Ramipril	Dec 16/Dec 18/Jan 19<br>Apr 2020<br>Jul 2020	3<br>1<br>1	API content<br>Description/ Dissolution/Assay<br>Assay	Recall/Alert	[1]
	Rosuvastatin	Apr 2020	3	Dissolution	Recall/Alert	[1]
	Cilnidipine	Apr 2020	1	API content	Recall/Alert	[1]
	Telmisartan	Jun 2020	1	Dissolution	Recall/Alert	[1]
	Diltiazem	May 2003	1	Falsified-contained only 50% API content	Seizure	[2]
	Amlodipine	Sep 2016	Unstated	"Elderly male patient taking tablet Amlodipine once daily [..] and had 2 years without any events. Patient came to the emergency department with the complaint of altered sensorium and elevated blood pressure [..] The Decreased Therapeutic Response could be due to be counterfeit drug"	Case report	[3]
Nigeria	Propanolol	1993-1996	Unstated	Falsified-no details available	Seizure	[4]

Country	API/combination of APIs	Year	No. Batch	Quality test failure	Report Type	Ref
**Pakistan**	Isosorbide Mononitrate	2011-2012	1	”Over 200 patients died because isosorbide tablets also contained high amount of pyrimethamine as a result of multiple negligence and errors during the manufacture”	Case report	[5] [6]
**United States**	Atorvastatin	2000	Unstated	”Nearly 200,000 tablets of Lipitor […] was found to be counterfeit and recalled by a small Missouri wholesaler. Some of the pills had already reached Rite Aid and CVS pharmacies.”	Recall	[7]
	Phenylephrine	Oct 2017	1	Potential microbial contamination	Recall/Alert	[8]
	Metoprolol	2019	Unstated	"Ethex recall of more than 60 products - metoprolol succinate, A physician in Clevelan Clinical found in the package for Ethex's metoprolol succinate[..] Dose not comply with the dissolution test of the USP monograph"	Recall/Alert	[9]
	Furosemide	Mar 2020	7	Substandard - Failure: Visible particulate matter (furosemide precipitate)	Recall/Alert	
	Heparin	Nov 2007	Unstated	152 ADRs associated with heparin	Case report	[10]
	Furosemide	2012	Unstated	“A patient is thirty-nine […] by 2012, his health was failing with his swollen legs, and the doctor namely Lever immediately suspected a poor quality of generic diuretic made by Ranbaxy. After Lever switched the prescription to a version made by American company Roxane and kept the same dose. Immediately, Parnell started losing fluid”	Case report	[9]
**Taiwan**	Rosuvastatin (Crestor)	2017	2	Falsified - containing atorvastatin	Recall/Alert	[11]
**United Kingdom**	Warfarin	Jul 2013	42	Substandard - Degradation products	Recall/Alert	[12]
	Atorvastatin (Lipitor®)	2005-2006	Unstated	Falsified – containing lovastatin	Recall/Alert	[13]
	Clopidogrel (Plavix®)	2007	Unstated	Falsified – containing simvastatin	Recall/Alert	[13]
	Clopidogrel	Unstated	Unstated	“The people responsible for this fraud were linked to the importation of two million doses of falsified clopidogrel, olanzapine, and bicalutamide into the United Kingdom. One of the ringleaders was sentenced to eight years’ imprisonment.”	Seizure	[14]
**Denmark**	Valsartan	2018	Unstated	Substandard – Cohort study: 198 participants exposed with valsartan developed a cancer (but no proof that the impurity caused it)	Case report	[15]

Country	API/combination of APIs	Year	No. Batch	Quality test failure	Report Type	Ref
**Germany + United States**	Heparin	2008	Unstated	Falsified - 900 cases of adverse events (238 deaths) reported to the US FDA and Federal Institute of Drugs and Medical Devices in Germany. Oversulfated chondroitin sulfate identified as the likely cause.	Case report	[16]
**Canada**	Amlodipine	2016	Unstated	"In 2016, law enforcement seized grey-market Norvasc pills [...] eleven patients of the pharmacy who were prescribed Norvasc died, the Ontario Coroner's Officer conducted an investigation into their deaths to determine if the counterfeit medication was a contributing factor"	Seizure	[17]
**Mexico**	Heparin	2020	Unstated	"Seven deaths at a hospital among patients treated with […] heparin and dozens of other patients reported to have suffered side effects […]. The pharma company has said that after examining the Inhepar 5000 IU / mL vials used at the hospital it concluded they had either been re-used – which is sometimes observed with falsified medicines – or tampered"	Case report	[18]
**Dominican Republic**	Cardiovascular medicines	2014	Unstated	Falsified – unknown<br>'Among the drugs seized there are painkillers, antibiotics and others used to combat hypertension and diabetes mellitus. During the operation, agents also seized empty boxes, containers and a machine to print the batch numbers and expiration dates of the fake products.'	Seizure	[19]
**Cameroon**	Hydrochlorothiazide	2019	1	Falsified- contain glibenclamide instead of hydrochlorothiazide "In March 2019, WHO was informed in Cameroon that a medicine presenting as hydrochlorothiazide 50mg had caused hypoglycaemia in patients"	Recall/ Alert	[20]
**Acronyms to be defined:** ADR, Adverse Drug Reaction, API, Active Pharmaceutical Ingredients, FDA, Food and Drug Administration; WHO, World Health Organization						

REFERENCES

1 Alerts. https://cdsco.gov.in/opencms/opencms/en/Notifications/Alerts/ (accessed 4 Sep 2019).

2 Database on the incidents of Counterfeit Medicines in the WHO-SEA Region. https://drive.google.com/file/d/1zUmFzuAnlzHyXJCSyX_L6fcGS3rvf1tG/view (accessed 16 Sep 2019).

3 USFDA. SCA Pharmaceuticals LLC. Issues Voluntary Nationwide Recall of Furosemide 100 mg in 0.9% Sodium Chloride Due to Presence of Precipitate | FDA. 2020.https://www.fda.gov/safety/recalls-market-withdrawals-safety-alerts/sca-pharmaceuticals-llc-issues-voluntary-nationwide-recall-furosemide-100-mg-09-sodium-chloride-due (accessed 13 Dec 2020).

4 Erhun W., Babalola O., Erhun M. Drug Regulation and Control in Nigeria: The Challenge of Counterfeit Drugs. *J Health Popul Dev Ctries* Published Online First: 2001.http://www.nigeriapharm.com/Library/Drug_regulation.pdf (accessed 16 Sep 2019).

5 Contaminated Isotab® (isosorbide mononitrate) incident in Lahore Pakistan. 2012;:1–2.https://www.who.int/medicines/publications/drugalerts/DrugSafetyAlert125.pdf

6 Etude de la qualite des medicaments generiques DCI achetes par la Pharmacie Populaire du Mali dans le cadre des appels d’offres de 2002 a 2005. http://apps.who.int/medicinedocs/en/m/abstract/Js22131en (accessed 6 Dec 2019).

7 Gaul. U.S. Prescription Drug System Under Attack. Washington Post. https://www.washingtonpost.com/archive/politics/2003/10/19/us-prescription-drug-system-under-attack/2ad69f71-42a0-4fce-829d-89fe0d4f3bd3/?… (accessed 13 Nov 2019).

8 USFDA. SCA Pharmaceuticals Issues Voluntary Nationwide Recall of Specific Products Due to Potential Contamination. US FDA. 2017.https://www.fda.gov/safety/recalls-market-withdrawals-safety-alerts/sca-pharmaceuticals-issues-voluntary-nationwide-recall-specific-products-due-potential-contamination (accessed 6 Sep 2019).

9 Li H. Bottle of Lies: The Inside Story of the Generic Drug Boom. *Yale J Biol Med* 2020;**93**:385–6.

10 Blossom DB, Kallen AJ, Patel PR, *et al.* Outbreak of Adverse Reactions Associated with Contaminated Heparin. *N Engl J Med* 2008;**359**:2674–84. doi:10.1056/NEJMoa0806450

11 Taylor P. Taiwan FDA seeks Crestor recall after counterfeit finds. 2019;:7–8.https://www.securingindustry.com/pharmaceuticals/taiwan-fda-seeks-crestor-recall-after-counterfeit-find/s40/a3433/#.XXHZ2CgzbIU

12 GOV.UK. Warfarin 3mg tablets - higher than expected levels of degradation. 2013.https://www.gov.uk/drug-device-alerts/drug-alert-warfarin-3mg-tablets-higher-than-expected-levels-of-degradation (accessed 6 Sep 2019).

13 Almuzaini T, Sammons H, Choonara I. Substandard and falsified medicines in the UK: A retrospective review of drug alerts (2001-2011). *BMJ Open* 2013;**3**:e002924. doi:http://dx.doi.org/10.1136/bmjopen-2013-002924

14 Newton PN, Schellenberg D, Ashley EA, *et al.* Quality assurance of drugs used in clinical trials: Proposal for adapting guidelines. *BMJ* 2015;**350**. doi:10.1136/bmj.h602

15 Pottegard A, Hallas J, Pottegard A, *et al.* Use of N-nitrosodimethylamine (NDMA) contaminated valsartan products and risk of cancer: Danish nationwide cohort study. *BMJ* 2018;**362**:k3851. doi:10.1136/bmj.k3851

16 Beyer T, Matz M, Brinz D, *et al.* Composition of OSCS-contaminated heparin occurring in 2008 in batches on the German market. *Eur J Pharm Sci* 2010;**40**:297–304. doi:10.1016/j.ejps.2010.04.002

17 Canada Government. Counterfeit Pharmaceuticals in Canada. 2016. http://publications.gc.ca/collections/collection_2013/sp-ps/PS64-108-2006-eng.pdf

18 Spectre of adulterated heparin emerges again, in Mexico. SecuringIndustry.com. https://www.securingindustry.com/pharmaceuticals/spectre-of-adulterated-heparin-emerges-again-in-mexico/s40/a11450/#.X9UGntj7TIX (accessed 13 Dec 2020).

19 Health authorities seize counterfeit medicines in Santiago. DominicanToday. 2014.https://dominicantoday.com/dr/local/2014/7/5/Health-authorities-seize-counterfeit-medicines-in-Santiago/ (accessed 16 Sep 2019).

20 WHO. Medical Product Alert N° 6/2019 (English version). 2019.https://www.who.int/news/item/17-04-2019-medical-product-alert-n-6-2019-(english-version) (accessed 13 Dec 2020).
