# Supplementary material for: The quality of medical products for cardiovascular diseases: a gap in global cardiac care
Source: BMJ Glob Health. 2021 Sep 14;6(9):e006523. doi: 10.1136/bmjgh-2021-006523 (PMC8442059; doi:10.1136/bmjgh-2021-006523)

Supplementary file 14: **PRISMA flow diagram of the selection process of the publications on cardiovascular medical devices quality**

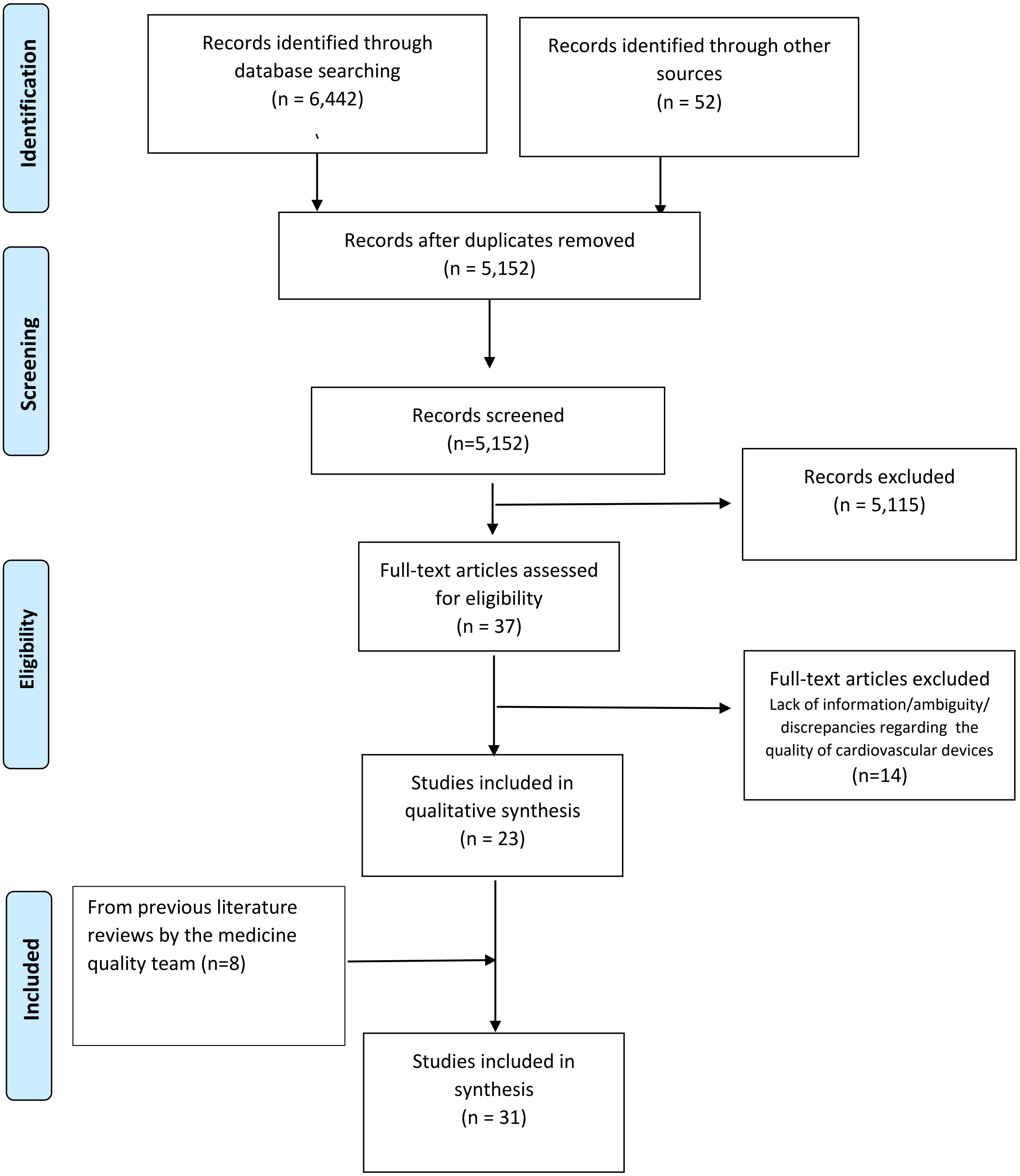

Supplement: Supplementary data [file bmjgh-2021-006523supp014.pdf]
