# Supplementary material for: The quality of medical products for cardiovascular diseases: a gap in global cardiac care
Source: BMJ Glob Health. 2021 Sep 14;6(9):e006523. doi: 10.1136/bmjgh-2021-006523 (PMC8442059; doi:10.1136/bmjgh-2021-006523)
Supplement: Supplementary data [file bmjgh-2021-006523supp015.pdf]

Supplementary file 15: Description of identified recalls/alerts, seizures and case-reports of substandard and falsified cardiovascular medical devices

Reference	Country	Date of incident	Type of publication	Information on the incidents
[1]	USA	1990-2002	Recall/Alert	8834 pacemakers malfunction - 30 deaths of patients associated, due to battery/capacitor abnormalities and electrical issues<br>7217 ICDs malfunctions - 31 deaths of patients associated
[2]	USA	1996-2003	Case report	A total of 103 (69%) of 150 death events were associated with defective pulse generators or high-voltage leads from five manufacturers (about 100 ICD model)
[3]	USA	1989-1992	Case-report	227 pacemakers with failure. All device failures were caused by loss of hermeticity due to fluid intrusion at the pulse generator header.
[4]	USA	1990-2000	Recall/Alert	Between January 1990 and December 2000, 408,500 pacemakers and 114,645 ICDs (523,145 total devices) were subject to recalls or safety alerts mainly due to hardware malfunctions and computer errors (accounted for 95% of device recalls)
[5]	USA	Feb 2018	Recall/ Alert	US FDA recalled 28 units of ICD and 20 units of Cardiac Resynchronization Therapy Defibrillator (CRT-D) due to gas mixture inside the device*, manufactured by Medtronic company
[6]	USA	Nov 2018	Recall/ Alert	US FDA recalled 4,778 units of Temporary Bipolar Pacing Lead due to the connector cap housing that may slide and expose the internal wire**
[7]	USA	Oct 2017	Recall/ Alert	US FDA Recalled 175,624 ICD and CRT-Ds manufactured by St Jude Medical due to premature battery depletion
[8]	USA	Apr 2019	Recall/ Alert	US FDA recalled 60 batches of 2 models: 830515F and 830705F : Miller Balloon Atrioseptostomy Catheter and Fogarty Dilation Atrioseptostomy Catheter because Balloon Deflation, Fragmentation and Detachment Issue. The US FDA says there's been one serious injury, but no deaths, due to balloons deflating, fragmenting, or detaching
[9]	USA	2005-2012	Recall/ Alert	From 2005-2012 a total of 66 cardiovascular device serious recalls were identified.
[10]	Worldwide	Oct 2016	Recall/ Alert	Worldwide recall of 398,740 ICD and CRT-Ds manufactured by St Jude Medical due to premature battery depletion, in which 251,346 were recalled in the US<br>Known impact:<br>37 people experienced dizziness due to battery issues<br>10 Reported fainting<br>2 People died due to premature battery depletion
[11]	USA	Feb 2019	Recall/ Alert	US FDA recalled 13,440 Dual Chamber Implantable Pulse Generators (IPGs) manufactured by Medtronic, Inc due to a circuit error that can result in a lack of cardiac pacing.
[12]	USA	Aug 2015	Recall/ Alert	US FDA recalled Total Artificial Heart (TAH-t) due to a specific component of the drive mechanism that may fail and cause the drive mechanism to stop pumping.

[13]	USA	Mar2019-Aug2020	Recall/ Alert	US FDA recalled 116 ICDs or CRT-D from 16 manufacture due to 47 recall reasons related to both hardware and software failures<br>Recall of 25 heart valve from 8 manufacture with 16 recall reasons related to hardware and software failures<br>Recall of 209 pacemakers from 40 manufacture with recall reasons related to hardware and software failures
[14]	USA	2003-2011	Case report	"This single-center retrospective case series included 90 of 448 patients who were implanted with a cardiac resynchronization therapy pacemaker at the Minneapolis Heart Institute from May 2003 through January 2011; this pacemaker was recalled in November 2015. [...] Five of 90 patients observed during 2015 experienced syncope when their pacemakers stopped pacing owing to battery or wire connection defects prior to the recall".
[15]	UK	Unstated	Recall/ Alert	The arterial pressure monitoring set has a tap that had been incorrectly placed during manufacture (batch number 59326333)
[16]	Japan	November 1994	Recall/ Alert	Recall of all lots of 4 pacemakers – Accufix® found with lead wires which penetrated the outer insulation
[17]	Pakistan	2016	**Seizure**	FIA seized fake stents at the Lahore's Mayo hospital, which were unregistered and sold by 3 companies in Lahore : Pak Punjab Cardex Medical System, AM System, and Saving Life Technologies***
[18]	Pakistan	Jan 2017	Recall/ Alert	DRA recovered 40 substandard stents of a multinational company***
[19]	USA	December 2018	Recall/ Alert	Recall of all lots of CoaguChek XS Test Strips (Warfarin INR test strips) manufactured by Roche Diagnostics, and distributed by Terrific Care, LLC. / Medex Supply Dist, Inc due to the risk of inaccurately reporting high INR test results
[20]	USA	October 2018	Recall/ Alert	Recall of 43 lots (more than 1.1 million packages) of CoaguChek XS PT manufactured by Roche Diagnostics due to inaccurately reporting high INR test results
[21]	UK	October 2018	Recall/ Alert	Recall of 3 products CoaguChek XS PT Test PST, CoaguChek XS PT Test, CoaguChek PT Test manufactured by Roche Diagnostics GmbH due to the risk of false high results for INR values above 4.5 when compared to laboratory results
[22]	Europe	2018	Case report	"Following commercialization of the MitraClip® Delivery System, cases of malfunctioning of the device, due to failure in clip detachment, were reported. After nine cases of malfunctioning, followed by severe comorbidity and complications, with one patients' decease - the company recalled the MitraClip® Delivery System manufactured from July 14, 2015 to August 11, 2015 and distributed from August 28, 2015 to February 3, 2016. The MitraClip® Delivery System, although defective in the presented case, passed all quality controls required by European legislation, bore the CE mark, and has been successfully applied several times. It passed two randomized studies (Everest I and Everest II)"
[23]	Ireland	2002-2012	Case report	ICD, A study was performed to follow patients who had the Riata defibrillator leads manufactured by St Jude Medical Inc and recalled in 2011 by the US FDA. "there were 52 individuals in the recalled lead group and 50 individuals in the control group from November 2002 to November 2012[…] In the recalled lead group, 7

				individuals (13.5%) had electrical dysfunction, 2 (3.8%) had EC, 7 (13.5%) had both, and 3 (5.7%) had lead fractures. […] In the control group, there were 4 with lead failure, 1 (2.0%) with electrical dysfunction, and 3 (6.0%) with lead fracture. there were 14 deaths in the recalled lead group but 10 deaths among controls".
[24]	Republic of North Macedonia	2018	Seizure	Seizure of falsified medical devices including 737 expired cardiac surgery instruments
[25]	Australia	2016	Recall/Alert	Medtronic Australasia Pty Ltd recalled Medtronic Reveal LINQ Insertable Cardiac Monitor (ICM) for the reason: a performance issue that affects the Recommended Replacement Time (RRT) alert
[25]	United States, Japan, Taiwan, Thailand and South Korea.	2016	Recall/Alert	Cordis Corporation recalled Cordis PRECISE (R) PRO RX Nitinol Stent System (Carotid) for the reason: Inability to deploy the stent or partial stent
[25]	Worldwide	2016	Recall/Alert	St. Jude Medical is recalling the Ellipse ICD (Implantable Cardioverter Defibrillator) due to the potential inability to deliver high voltage therapy. Model CD2411-36Q, CD2411-36C, CD1411-36Q, CD1411-36C
[25]	Worldwide	2016	Recall/Alert	Datascope Corporation recalled Intra-Aortic Balloon Catheter for the reason: the IFU for the STATLOCK Sheath Stabilization device for Percutaneous Sheath Introducer (Vendor PN 2403097-1405R) was erroneously packaged with another kits
[26]	Australia, Andorra, Belgium, Brazil, Canada, Colombia, Czech Republic, Denmark, El Salvador, Finland, France, Germany, Greece, Hong Kong, India, Ireland, Italy, Japan, Lebanon, Malaysia, Netherlands, New Zealand, Peru, Philippines, Poland, Portugal, Russia Federation,,Saudi Arabia, Spain, Sweden, Singapore, Serbia, Solvenia, Turkey, United Kingdom,	Up to 31 Aug 2019	Recall/Alert	Total of 5,738 events relevant to recall/alert cardiovascular devices (pacemakers, ICDs, stents, and other devices) in the International Medical Devices Database

	Switzerland, United States			
* May prevent the device from delivering the electrical shock needed<br>** May cause loss of connectivity or even breakage during movement of the cables and prevent the attached external pulse generator from pacing<br>***We were unable to find additional information on the potential defect(s) of the stents				
ICD, Implantable Cardioverter Defibrillator; CRT-D, Cardiac Resynchronization Therapy Defibrillator; INR, International Normalised Ratio; FDA, Food and Drugs Administration; US, United States; UK, United Kingdom; FIA, Federal Investigation Agency ; DRA, Drug Regulatory Authority; LLC, Limited Liability Company				

1 Maisel W, Moynahan M, Zuckerman B, *et al.* Pacemaker and ICD generator malfunctions: analysis of Food and Drug Administration annual reports. *jamanetwork.com*https://jamanetwork.com/journals/jama/article-abstract/202752 (accessed 28 Mar 2019).

2 Hauser RG, Kallinen L. Deaths associated with implantable cardioverter defibrillator failure and deactivation reported in the United States Food and Drug Administration Manufacturer and User Facility Device Experience Database. *Hear Rhythm* 2004;**1**:399–405. doi:http://dx.doi.org/10.1016/j.hrthm.2004.05.006

3 Nanthakumar K, Dorian P, Ham M, *et al.* When Pacemakers Fail: An Analysis of Clinical Presentation and Risk in 120 Patients with Failed Devices. *Pacing Clin Electrophysiol* 1998;**21**:87–93. doi:10.1111/j.1540-8159.1998.tb01065.x

4 Maisel W, Sweeney M, Stevenson W, *et al.* Recalls and Safety Alerts Involving Pacemakers and Implantable Cardioverter-Defibrillator Generators. *jamanetwork.com* doi:doi:10.1001/jama.286.7.793

5 Medtronic Recalls Cardiac Resynchronization Therapy and Implantable Cardioverter Defibrillators Due to Manufacturing Error Preventing Electrical Shock Delivery. 2018.https://www.fda.gov/medical-devices/medical-device-recalls/medtronic-recalls-cardiac-resynchronization-therapy-and-implantable-cardioverter-defibrillators-due (accessed 19 Sep 2019).

6 USFDA. Oscor Recalls TB Temporary Bipolar Pacing Leads Due to Connector Separation Causing Potential for Loss of Pacing. 2018.https://www.fda.gov/medical-devices/medical-device-recalls/oscor-recalls-tb-temporary-bipolar-pacing-leads-due-connector-separation-causing-potential-loss (accessed 19 Sep 2019).

7 USFDA. Update - St. Jude Medical Recalls Implantable Cardioverter Defibrillators (ICD) and Cardiac Resynchronization Therapy Defibrillators (CRT-D) Due to Premature Battery Depletion | FDA. 2017.https://www.fda.gov/medical-devices/medical-device-recalls/update-st-jude-medical-recalls-implantable-cardioverter-defibrillators-icd-and-cardiac (accessed 19 Sep 2019).

8 USFDA. Edwards Lifesciences Recalls Miller and Fogarty Atrioseptostomy Dilation Catheters Due to Balloon Deflation, Fragmentation and Detachment Issue. 2019.https://www.fda.gov/medical-devices/medical-device-recalls/edwards-lifesciences-recalls-miller-and-fogarty-atrioseptostomy-dilation-catheters-due-balloon (accessed 15 Dec 2020).

9 Somberg JC, McEwen P, Molnar J. Assessment of cardiovascular and noncardiovascular medical device recalls. *Am J Cardiol* 2014;**113**:1899–903. doi:10.1016/j.amjcard.2014.03.024

10 T. Turner. St. Jude Medical Implantable Defibrillators. 2019.https://www.drugwatch.com/defibrillators/ (accessed 19 Sep 2019).

11 USFDA. Medtronic, Inc. Recalls Dual Chamber Implantable Pulse Generators (IPGs) Due to Possible Circuit Error. 2019.https://www.fda.gov/medical-devices/medical-device-recalls/medtronic-inc-recalls-dual-chamber-implantable-pulse-generators-ipgs-due-possible-circuit-error (accessed 19 Sep 2019).

12 USFDA. Class 1 Device Recall Temporary Total Artificial Heart (TAHt) with Freedom. Published Online First: 2015.https://www.accessdata.fda.gov/scripts/cdrh/cfdocs/cfRes/res.cfm?id=139311 (accessed 19 Sep 2019).

13 USFDA. Medical Device Recalls Database. https://www.accessdata.fda.gov/scripts/cdrh/cfdocs/cfres/res.cfm (accessed 16 Dec 2020).

14 Sengupta J, Storey K, Casey S, *et al.* Outcomes before and after the Recall of a Heart Failure Pacemaker. *JAMA Intern Med* 2020;**180**:198–205. doi:10.1001/jamainternmed.2019.5171

15 Hingston C, Muzuni D, Surridge M. Defective arterial pressure monitoring set. *Anaesthesia* 2013;**68**:540–540. doi:10.1111/anae.12224

16 WHO. Pacemakers- Warning Concerning Dangerous Wiring Defect. Published Online First: 1994.https://www.who.int/medicines/publications/drugalerts/drug_alert48defective_pacemakers.pdf?ua=1 (accessed 18 Sep 2019).

17 Hakim B. FIA Exposes Mafia Selling Fake Stents at Over 3200% Markup. 2016.https://propakistani.pk/2017/01/18/fia-exposes-mafia-selling-fake-stents-3200-markup/ (accessed 18 Sep 2019).

18 DRA recovers 40 substandard cardiac stents, seals company's office. 2017.https://www.pakistantoday.com.pk/2017/01/22/dra-recovers-40-substandard-cardiac-stents-seals-companys-office/ (accessed 18 Sep 2019).

19 USFDA. Terrific Care, LLC. / Medex Supply Dist, Inc. Issues Nationwide Recall of CoaguChek Test Strips. 2018.https://www.fda.gov/safety/recalls-market-withdrawals-safety-alerts/terrific-care-llc-medex-supply-dist-inc-issues-nationwide-recall-coaguchek-test-strips (accessed 18 Sep 2019).

20 USFDA. Roche Diagnostics Recalls CoaguChek XS PT Test Strips Due to Inaccurate INR Test Results. 2018.https://www.fda.gov/medical-devices/medical-device-recalls/roche-diagnostics-recalls-coaguchek-xs-pt-test-strips-due-inaccurate-inr-test-results (accessed 18 Sep 2019).

21 MHRA medical device alert – CoaguChek Test Strips for Point of Care and Home Use (Roche Diagnostics GmbH) : PSNC Main site. 2018.https://psnc.org.uk/our-news/mhra-medical-device-alert-coaguchek-test-strips-for-point-of-care-and-home-use-roche-diagnostics-gmbh/ (accessed 18 Sep 2019).

22 A. G, A. S, M. P. On a defective Mitraclip system: Considerations on the medical device regulation in Europe. *Monaldi Arch Chest Dis* 2018;**88**:29–32. doi:http://dx.doi.org/10.4081/monaldi.2018.901

23 Davidsson GA, Jonsdottir GM, Oddsson H. Long-term outcome of implantable cardioverter/defibrillator lead failure. *JAMA Intern Med* Published Online First: 2020.https://jamanetwork.com/journals/jamainternalmedicine/article-abstract/2757528

24 Illicit online pharmaceuticals: 500 tonnes seized in global operation. https://www.interpol.int/en/News-and-Events/News/2018/Illicit-online-pharmaceuticals-500-tonnes-seized-in-global-operation (accessed 8 Oct 2019).

25 EU Commission. Data set compilation of Alerts and Recalls. *JRC Tech Rep*

26 International Medical Devices Database_Cardiovascular devices. https://medicaldevices.icij.org/search?f%5Bdevice-classification%5D=Cardiovascular+Devices&m=Event&q%5Bdisplay_cont%5D= (accessed 11 Jun 2019).
