# Supplementary material for: The quality of medical products for cardiovascular diseases: a gap in global cardiac care
Source: BMJ Glob Health. 2021 Sep 14;6(9):e006523. doi: 10.1136/bmjgh-2021-006523 (PMC8442059; doi:10.1136/bmjgh-2021-006523)
Supplement: Supplementary data [file bmjgh-2021-006523supp016.pdf]

Supplementary file 16. Other articles on cardiovascular medicines quality included in the review

Title	First author	Year
Aspirin-A National Survey I: Semiautomated Determination of Aspirin in Bulk and Tablet Formulations and Salicylic Acid in Tablet Formulations [1]	William E. Juhl	1979
Monitoring the Purity of Pharmaceutical Heparin Preparations by High-Field 1H-Nuclear Magnetic Resonance Spectroscopy [2]	Neville, George A	1988
Drug Quality Control Work in DARU: Observations during 1983-1986 [3]	Kibwage, I.O.	1992
Quality Control of Pharmaceuticals In The Caribbean [4]	Dennis.P	1992
Drug Quality Control Work in Drug Analysis and Research Unit: Observation During 1991-1995 [5]	Kibwage, I.O.	1999
Drug Quality Control in Kenya: Observation in Drug Analysis and Research Unit during the Period 1996-2000 [6]	Thoithi, G.N.	2002
Magic Bullet Gone Astray: provides guidance to organizations for imple- menting effective anti-counterfeiting policies Medications and the Internet [7]	Michael A.Veronin	2004
Mongolia Pharmaceutical Sector Assessment Report [8]	Salik Govind	2004
Substandard life-saving drugs: a global concern [9]	Andreotti. F	2005
UV spectrophotometric determination of clopidogrel and repaglinide [10]	Sankar, D. G.	2005
Étude de la qualité des médicaments génériques DCI achetés par la Pharmacie Populaire du Mali dans le cadre des appels d'offres de 2002 à 2005 [11]	Issiaka C	2006
Monitoring of simvastatin impurities by HPLC with microemulsion eluents [12]	Malenovic,A	2006
Amlodipine besilate screening in pharmaceutical preparations by CE [13]	P Jankovics	2008
Contaminated heparin preparations, severe adverse events and the contact system [14]	Ramacciotti, E	2008
Contaminated heparin products recalled [15]	WHO Drug information	2008
Drug Quality Control in Kenya: Observation in the Drug Analysis and Research Unit During the Period 2001-2005 [16]	Thoithi.G.N	2008
Heparin quality control in the Brazilian market: implications in the cardiovascular surgery [17]	MELO, E	2008
Les heparines contaminees [18]	Monneret, C	2008
UK Government action on counterfeit medicines [19]	The pharmaceutical journal	2008
Analysis of pharmaceutical heparins and potential contaminants using 1H-NMR and PAGE [20]	Zhang, Z	2009
Comparative stability of repackaged metoprolol tartrate tablets [21]	Y Yang	2009
Counterfeit Drugs: Coming to a Pharmacy Near You with an Update for 2009 [22]	Yankus. W	2009
Identifying Counterfeit Medicines Using near Infrared Spectroscopy [23]	Moffat, AC	2009
Quality of medicines stored together in multi-compartment compliance aids [24]	P. Donyai	2009
Raids uncover counterfeit drugs [25]	Sagita, Dessy	2009
Comparison of established and novel purity tests for the quality control of heparin by means of a set of 177 heparin samples [26]	Alban,S	2010
Composition of OSCS-contaminated heparin occurring in 2008 in batches on the German market [27]	T. Beyer	2010
Los medicamentos falsificados en Peru [28]	Moreno Exebio,LE	2010
Post Marketing Surveillance on Propranolol and Atenolol Tablets Manufactured in Iran [29]	Khabnadideh,S	2010

Title	First author	Year
Tablet splitting: Product quality assessment of metoprolol succinate extended release tablets [30]	Na Zhao	2010
Contaminants in heparin: Review of the literature, molecular profiling, and clinical implications [31]	Eduardo Ramacciotti	2011
In vitro-in vivo correlation of four commercial brands of aspirin tablets marketed in Nigeria [32]	Bamigbola E. A	2011
Simple fluorescence assay for quantification of OSCS in heparin. [33]	Luhn, S	2011
Unveiling the Mystery of Online Pharmacies: an Audit Study [34]	Bate, R.	2012
Atorvastatin Generics Obtained from Multiple Sources Worldwide Contain a Methylated Impurity that Reduces Their HMG-CoA Reductase Inhibitory Effects [35]	Richard Preston Mason	2013
Characterization of currently marketed heparin products: Key tests for LMWH quality assurance [36]	Hongping Ye	2013
In vitro studies of amlodipine besylate tablet and comparison with foreign brand leader in Nepal [37]	Tekendra Pant	2013
Liquid dosage forms extemporaneously prepared from commercially available products - considering new evidence on stability [38]	Haywood, A.	2013
Quality Performance of Drugs Analyzed in the Drug Analysis and Research Unit (DARU) during the Period 2006-2010 [39]	Abuga K.O.	2013
RP-LC simultaneous quantitation of co-administered drugs for (non-insulin dependent) diabetic mellitus induced dyslipidemia in active pharmaceutical ingredient, pharmaceutical formulations and human serum with UV-detector [40]	Arayne, M	2013
Substandard drugs: A potential crisis for public health [41]	Johnston,A	2013
A systematic review of counterfeit and substandard medicines in field quality surveys [42]	Abdulaziz, A.F.	2014
Chromatographic determination of clopidogrel bisulfate; detection and quantification of counterfeit Plavix tablets [43]	Mona E. ElTantawy	2014
Degradation study of different brands of amlodipine using UV spectrophotometer [44]	Naveed, S	2014
Development and validation of HPLC dissolution assay of simvastatin tablets under normal and accelerated conditions [45]	EL Karbanea,M	2014
Evaluation of the Pharmaceutical Characteristics of Various Enteric-Coated Aspirin Tablets under Different Storage Conditions [46]	Abe,T	2014
Liquid chromatography with tandem mass spectrometry for the simultaneous identification and quantification of cardiovascular drugs applied to the detection of substandard and falsified drugs [47]	M Bernard	2014
Quality and Avialability of Medicines in Public Health Facilities of Kerala, Indian State an Analysis [48]	Lekshmi. S	2014
Recent Applications of Analytical techniques for counterfeit drug analysis: A Review [49]	Kumar, R.	2014
Drug Quality: Postmarket Sampling and Testing Results for Drugs (FY 2015) [50]	US FDA	2015
Surveillance of Quality of Medicines Available in the Nepalese Market: A Study from Kathmandu Valley [51]	Gyanwali, P.	2015
In vitro and in vivo postmarketing surveillance of valsartan, alone or in combination with amlodipine or hydrochlorothiazide, among Palestinian hypertensive patients [52]	Zaid,N.A	2016
National Drug Survey - The Core Expert Committee [53]	Surinder Singh	2016
Process validation of tablet containing Irbesartan 300mg and Hydrochlorothiazide 12.5mg [54]	Z Hussain	2016
Drug Quality: Postmarket Sampling and Testing Results for Drugs (FY 2016) [55]	US FDA	2017
Identification of Substandard Medicines via Disproportionality Analysis of Individual Case Safety Reports.[56]	Trippe, Z. A.	2017
Investigations Of The Quality Medicines Distributed In Myanmar And Cambodia, Through Different Surveys [57]	Islam, R.	2017

Title	First author	Year
Poor-Quality and Counterfeit Drugs: A Systematic Assessment of Prevalence and Risks Based on Data Published From 2007 to 2016. [58]	Koczwara, A.	2017
A Study on Spurious and Not of Standard Quality Drugs in the State of Andhra Pradesh [59]	Praveen Kumar G	2018
After Valsartan Recalls, Regulators Grapple with Nitrosamine Contamination in APIs [60]	Shanley A	2018
Comparison of uv spectrophotometry and hplc methods for the determination of pharmaceutical equivalence of clopidogrel tablet brands marketed in Nigeria [61]	S. E. Ukwueze	2018
Development and validation of a hydrophilic interaction liquid chromatography method for the quantitation of impurities in fixed-dose combination tablets containing rosuvastatin and metformin [62]	Machairas. G	2018
Inorganic analysis of falsified medical products using X-ray fluorescence spectroscopy and chemometrics [63]	Herve.R	2018
NDMA impurity in valsartan and other pharmaceutical products:Analytical methods for the determination of N-nitrosamines [64]	Parr.K.M	2018
Regulatory response to contaminated valsartan [65]	Rita Banzi	2018
WHO pharmaceuticals Newsletter No.5 2018 [66]	WHO	2018
Analysis of an impurity, N-nitrosodimethylamine, in valsartan drug substances and associated products using GC-MS [67]	Tsutsumi.T	2019
Current status of angiotensin receptor blocker recalls[68]	P Moon Gunasekaran	2019
Development of quantitative HPTLC-densitometry methods following a model process for transfer of TLC screening methods for pharmaceutical products containing moxifloxacin HCl, ofloxacin, amoxicillin trihydrate, acetylsalicylic acid + acetaminophen + caffeine, nimesulide, irbesartan, and pantoprazole [69]	Zeng, B	2019
Falsified and substandard cardiovascular drugs in Africa: a need for continued monitoring strategies [70]	Marie Antignac	2019
Low quality of some generic cardiovascular medicinal products represents a matter for growing concern [71]	Juan Tamargo	2019
Public Perception toward E-commerce of Medicines and Comparative Pharmaceutical Quality Assessment Study of Two Different Products of Furosemide Tablets from Community and Illicit Online Pharmacies [72]	Ashames, A	2019
Short commentary on NDMA (N-nitrosodimethylamine) contamination of valsartan products [73]	David J. Snodin	2019
Defibrotide falsifie et contamine identifie dans les regions de l'OMS de l'Europe, Mediterranee orientale et du Pacifique occidental [74]	-1	2020
Substandard, falsified and unregistered medicines in Latin America, 2017-2018 [75]	Rojas-Cortes, R.	2020
Stability of essential drugs in the field: Results of a study conducted over a two-year period in Burkina Faso [76]	Ballereau.F	1997

1 Juhl WE, Kirchhoefer RD. Aspirin—a national survey I: Semiautomated determination of aspirin in bulk and tablet formulations and salicylic acid in tablet formulations. *J Pharm Sci* 1980;**69**:544–8. doi:10.1002/jps.2600690517

2 Neville GA, Mori F, Holme KR, *et al.* Monitoring the purity of pharmaceutical heparin preparations by high-field 1H-nuclear magnetic resonance spectroscopy. *J Pharm Sci* 1988;**78**:101–4. doi:http://dx.doi.org/10.1002/jps.2600780205

3 Kibwage IO, Ogeto JO, Maitai CK, *et al.* Drug quality control work in Daru: observations during 1983-1986. *East Afr Med J* 1992;**69**:577–80.http://www.ncbi.nlm.nih.gov/pubmed/1473513 (accessed 25 Sep 2019).

4 Dennis P, Edgrilll L., Allen M. Quality control of pharmaceuticals in the Caribbean. Int. Pharm. J. http://ovidsp.ovid.com/ovidweb.cgi?T=JS&PAGE=reference&D=emed4&NEWS=N&AN=22151866 (accessed 25 Sep 2019).

5 Kibwage I, JT K, Gathu L, *et al.* Drug Quality Control Work in Drug Analysis and Research Unit: Observation During 1991-1995. Published Online First: 1999.http://erepository.uonbi.ac.ke/handle/11295/10357?show=full (accessed 25 Sep 2019).

6 Thoithi G, Abuga K, Nguyo J, *et al.* Drug Quality Control in Kenya: Observation in Drug Analysis and Research Unit during the Period 1996-2000. *East Cent African J Pharm Sci* 2004;**5**:28–32. doi:10.4314/ecajps.v5i2.9684

7 Veronin MA, Youan B-BC. Magic bullet gone astray: medications and the Internet. *Science (80-)* 2004;**305**:481–2.https://go.gale.com/ps/anonymous?id=GALE%7CA120350491&sid=googleScholar&v=2.1&it=r&linkaccess=fulltext&issn=00368075&p=AONE&sw=w (accessed 25 Sep 2019).

8 Govind S. Mongolia Pharmaceutical Sector Assessment Report. Published Online First: 2004.http://apps.who.int/medicinedocs/documents/s18614en/s18614en.pdf (accessed 25 Sep 2019).

9 Andreotti F, Crea F. Substandard life-saving drugs: a global concern. *Eur Heart J* 2005;**26**:858–60. doi:10.1093/eurheartj/ehi219

10 Gowri Sankar D, Kalyan Sumanth S, Pawar AK., *et al.* UV spectrophotometric determination of clopidogrel and repaglinide. *Asia J Chem* 2005;**17**.http://www.asianjournalofchemistry.co.in/User/ViewFreeArticle.aspx?ArticleID=17_3_99 (accessed 25 Sep 2019).

11 Issiaka CAMARA. Étude de la qualité des médicaments génériques DCI achetés par la Pharmacie Populaire du Mali dans le cadre des appels d'offres de 2002 à 2005.

12 Malenovic A, Medenica M, Ivanovic D. Monitoring of simvastatin impurities by HPLC with microemulsion eluents. *Chromatographia* 2006;**63**:S95–100. doi:http://dx.doi.org/10.1365/s10337-006-0747-4

13 Jankovics P, Németh T, Németh-Palotás J, *et al.* Amlodipine besilate screening in pharmaceutical preparations by CE. *Chromatographia* 2008;**68**:S43–8. doi:http://dx.doi.org/10.1365/s10337-008-0620-8

14 Ramacciotti E, Wahi R, Messmore HL, *et al.* Contaminated heparin preparations, severe adverse events and the contact system. *Clin Appl Thromb* 2008;**14**:489–91. doi:http://dx.doi.org/10.1177/1076029608324291

15 WHO. Contaminated heparin products recalled. *WHO Drug Inf* 2008;**22**:10.http://www.who.int/medicines/publications/druginformation/issues/22_1_2008.pdf

16 Thoithi G., Abuga K., Nguyo J. Drug Quality Control in Kenya: Observation in the Drug Analysis and Research Unit During the Period 2001-2005. East Cent. African J. Pharm. Sci. 2008;:74–81.https://www.researchgate.net/publication/272459027_Drug_Quality_Control_in_Kenya_Observation_in_the_Drug_Analysis_and_Research_Unit_During_the_Period_2001-2005 (accessed 25 Sep 2019).

17 Melo E, Pereira M, Cunha R, *et al.* Heparin quality control in the Brazilian market: Implications in the cardiovascular surgery. *Brazilian J Cardiovasc Surg* 2008;**23**:169–74.http://www.scielo.br/pdf/rbccv/v23n2/en_v23n2a04.pdf

18 Monneret C. Les héparines contaminées. *Ann Pharm Fr* 2008;**66**:212–5. doi:http://dx.doi.org/10.1016/j.pharma.2008.07.004

19 UK Government action on counterfeit medicines. Pharm. J. https://www.pharmaceutical-journal.com/opinion/comment/uk-government-action-on-counterfeit-medicines/10008049.article?firstPass=false (accessed 25 Sep 2019).

20 Zhang Z, Li B, Suwan J, *et al.* Analysis of pharmaceutical heparins and potential contaminants using 1H-NMR and PAGE. *J Pharm Sci* 2009;**98**:4017–26. doi:http://dx.doi.org/10.1002/jps.21729

21 Yang Y, Gupta A, Carlin A, *et al.* Comparative stability of repackaged metoprolol tartrate tablets. *Elsevier* doi:https://doi.org/10.1016/j.ijpharm.2009.10.040

22 Yankus W. Counterfeit Drugs: Coming to a Pharmacy Near You with an Update for 2009. Am. Counc. Sci. Heal. 2009.https://drive.google.com/file/d/1Y-6zRTH6GHabWIMnV15JbpTMMum6lb_d/view (accessed 3 Jun 2019).

23 Moffat AC, Assi S, Watt RA. Identifying Counterfeit Medicines Using near Infrared Spectroscopy. *J Near Infrared Spectrosc* 2010;**18**:1–15. doi:10.1255/jnirs.856

24 Quality of medicines stored together in multi-compartment compliance aids. *J Clin Pharm Ther* 2010;**35**:533–43. doi:http://dx.doi.org/10.1111/j.1365-2710.2009.01129.x

25 Sagita D. Raids uncover counterfeit drugs. 2009.https://drive.google.com/file/d/14plcZWR6p6NYjMbI1szhONmmAT8oHFYV/view (accessed 16 Dec 2020).

26 Alban S, Lühn S, Schiemann S, *et al.* Comparison of established and novel purity tests for the quality control of heparin by means of a set of 177 heparin samples. *Springer* doi:10.1007/s00216-010-4169-7

27 Beyer T, Matz M, Brinz D, *et al.* Composition of OSCS-contaminated heparin occurring in 2008 in batches on the German market. *Eur J Pharm Sci* 2010;**40**:297–304. doi:10.1016/j.ejps.2010.04.002

28 Exebio LEM, Rodríguez J, Sayritupac F. Los medicamentos falsificados en Perú. *Rev Panam Salud Pública* 2010;**27**:138–43. doi:10.1590/S1020-49892010000200008

29 Iranian Association of Pharmaceutical Scientists. S, Mohammadi Samani S, Yarmohammadi G, *et al. Post Marketing Surveillance on Propranolol and Atenolol Tablets Manufactured in Iran*. Iranian Association of Pharmaceutical Scientists 2010. http://www.ijps.ir/article_2110.html (accessed 12 Jun 2019).

30 Zhao N, Zidan A, Tawakkul M, *et al.* Tablet splitting: Product quality assessment of metoprolol succinate extended release tablets. *Int J Pharm* 2010;**401**:25–31. doi:http://dx.doi.org/10.1016/j.ijpharm.2010.09.004

31 Ramacciotti E, Clark M, Sadeghi N, *et al.* Contaminants in heparin: Review of the literature, molecular profiling, and clinical implications. *Clin Appl Thromb* 2011;**17**:126–35. doi:http://dx.doi.org/10.1177/1076029610392214

32 Bamigbola E A, Ibrahim M A, Attama A A, *et al.* In vitro-in vivo correlation of four commercial brands of aspirin tablets marketed in Nigeria. *African J Pharm Pharmacol* 2011;**5**:1648–54. doi:10.5897/AJPP10.179

33 Luhn S, Schiemann S, Alban S, *et al.* Simple fluorescence assay for quantification of OSCS in heparin. *Anal Bioanal Chem* 2011;**399**:673–80. doi:10.1007/s00216-010-3867-5

34 Bate R, Jin GZ, Mathur A. Unveiling the Mystery of Online Pharmacies: an Audit Study. 2012.https://www.researchgate.net/publication/254072335_Unveiling_the_Mystery_of_Online_Pharmacies_an_Audit_Study (accessed 25 Sep 2019).

35 Mason RP, Jacob R. Atorvastatin generics obtained from multiple sources worldwide contain a methylated impurity that reduces their hmg-coa reductase inhibitory effects. *J Clin Lipidol* 2013;**7**:287. doi:http://dx.doi.org/10.1016/j.jacl.2013.03.096

36 Ye H, Toby TK, Sommers CD, *et al.* Characterization of currently marketed heparin products: Key tests for LMWH quality assurance. *J Pharm Biomed Anal* 2013;**85**:99–107. doi:http://dx.doi.org/10.1016/j.jpba.2013.06.033

37 Pant T, Subedi KMRK. In vitro studies of amlodipine besylate tablet and comparison with foreign brand leader in Nepal. 2013.https://ijpsr.com/bft-article/in-vitro-studies-of-amlodipine-besylate-tablet-and-comparison-with-foreign-brand-leader-in-nepal/ (accessed 16 Dec 2020).

38 Haywood A, Glass BD. Liquid dosage forms extemporaneously prepared from commercially available products - Considering new evidence on stability. *J Pharm Pharm Sci* 2013;**16**:441–55.http://ejournals.library.ualberta.ca/index.php/JPPS/article/view/19411/15507

39 Abuga KO, Amugune BK, Ndwigah SN, *et al.* Quality Performance of Drugs Analyzed in the Drug Analysis and Research Unit (DARU) during the Period 2006-

2010. *East Cent African J Pharm Sci* 2013;**16**.https://profiles.uonbi.ac.ke/okibwage/files/full_paper.pdf (accessed 25 Sep 2019).

40 Arayne MS, Sultana N, Tabassum A. RP-LC simultaneous quantitation of co-administered drugs for (non-insulin dependent) diabetic mellitus induced dyslipidemia in active pharmaceutical ingredient, pharmaceutical formulations and human serum with UV-detector. *Clin Chim Acta* 2013;**425**:54–61. doi:10.1016/j.cca.2013.06.020

41 Johnston A, Holt DW. Substandard drugs: a potential crisis for public health. *Br J Clin Pharmacol* 2014;**78**:218–43. doi:10.1111/bcp.12298

42 Alghannam A, Evans S, Schifano F, *et al.* A systematic review of counterfeit and substandard medicines in field quality surveys. *Integr Pharm Res Pract* 2014;**3**:71. doi:10.2147/IPRP.S63690

43 ETantawy M., Bebawy L. Chromatographic determination of clopidogrel bisulfate; detection and quantification of counterfeit Plavix tablets. *Bull Fac Pharmacy, Cairo Univ* 2014;**52**:91–101. doi:http://dx.doi.org/10.1016/j.bfopcu.2014.04.003

44 Naveed S, Jawaid W. Degradation study of different brands of amlodipine using UV spectrophotometer. *J Sci Innov Res*http://www.jsirjournal.com/Vol3_Issue4_04.pdf (accessed 26 Mar 2019).

45 El Karbane M, Ramli Y, Amood Al-Kamarany M, *et al.* Development and validation of HPLC dissolution assay of simvastatin tablets under normal and accelerated conditions. *J Chem Pharm Res* 2014;**6**:886–93.http://jocpr.com/vol6-iss6-2014/JCPR-2014-6-6-886-893.pdf

46 Abe T, Yanagihara Y, Uchino T, *et al.* Evaluation of the Pharmaceutical Characteristics of Various Enteric-Coated Aspirin Tablets under Different Storage Conditions. *Chem Pharm Bull* 2014;**62**:617–26. doi:10.1248/cpb.c13-00580

47 Bernard M, Akrout W, Van Buu CT, *et al.* Liquid chromatography with tandem mass spectrometry for the simultaneous identification and quantification of cardiovascular drugs applied to the detection of substandard and falsified drugs. *J Sep Sci* 2015;**38**:562–70. doi:10.1002/jssc.201401301

48 Lekshmi S, Mohanta G., Farooq A, *et al.* Quality and Avialability of Medicines in Public Health Facilities of Kerala, Indian State an Analysis. *Int J Adv Sci Eng Technol* 2014;**2**.http://www.iraj.in/journal/journal_file/journal_pdf/6-87-142044869595-97.pdf (accessed 25 Sep 2019).

49 Kumar R. Recent applications of analytical techniques for counterfeit drug analysis: A review. *Int J PharmTech Res* 2014;**6**:646–65.http://sphinxsai.com/2014/PTVOL6/PT=31(646-665)AJ14.pdf

50 Drug Quality: Postmarket Sampling and Testing Results for Drugs (FY 2015) | FDA. US FDA. 2015.https://www.fda.gov/media/103649/download (accessed 25 Sep 2019).

51 Gyanwali P, Humagain BR, Aryal KK, *et al.* Surveillance of Quality of Medicines Available in the Nepalese Market: A Study from Kathmandu Valley. *J Nepal Health Res Counc* 2015;**13**:233–40.http://ovidsp.ovid.com/ovidweb.cgi?T=JS&PAGE=reference&D=emed16&NEWS=N&AN=616144386

52 Zaid AN, Ghanem M, Shweiki D, *et al.* In vitro and in vivo postmarketing surveillance of valsartan, alone or in combination with amlodipine or hydrochlorothiazide, among palestinian hypertensive patients. *Ther Clin Risk Manag* 2016;**12**:1425–32. doi:http://dx.doi.org/10.2147/TCRM.S110727

53 Surinder Singh. National Drug Survey - The Core Expert Committee. Natl. Inst. Biol. 2016.http://nib.gov.in/Drug Survey.html (accessed 25 Sep 2019).

54 Hussain Z, Baqir S, Naqvi S, *et al.* Process validation of tablet containing Irbesartan 300mg and Hydrochlorothiazide 12.5mg. *Int J Sci Eng Res* 2016.

55 Drug Quality: Postmarket Sampling and Testing Results for Drugs (FY 2016) | FDA. US FDA. 2016.https://www.fda.gov/media/103635/download (accessed 25 Sep 2019).

56 Trippe ZA, Brendani B, Meier C. Identification of Substandard Medicines via Disproportionality Analysis of Individual Case Safety Reports. *Drug Saf* 2017;**40**:293–303. doi:http://dx.doi.org/10.1007/s40264-016-0499-5

57 Islam R. Investigations Of The Quality Medicines Distributed In Myanmar And Cambodia, Through Different Surveys. Grad. Sch. Med. Sci. Technol. Kanazawa Univ. https://kanazawa-u.repo.nii.ac.jp/?action=repository_action_common_download&item_id=43361&item_no=1&attribute_id=31&file_no=1 (accessed 9 Dec 2019).

58 Koczwara A, Dressman J. Poor-Quality and Counterfeit Drugs: A Systematic Assessment of Prevalence and Risks Based on Data Published From 2007 to 2016. *J Pharm Sci* 2017;**106**:2921–9. doi:10.1016/j.xphs.2017.05.018

59 Praveen Kumar G, Ravi Shankar A. A Study on Spurious and Not of Standard Quality Drugs in the State of Andhra Pradesh. *African J Pharm Pharmacol* Published Online First: 2018.https://academicjournals.org/journal/AJPP/article-full-text-pdf/4EC3AD856268 (accessed 25 Sep 2019).

60 Shanley A. After Valsartan Recalls, Regulators Grapple with Nitrosamine Contamination in APIs. Published Online First: 2018.http://www.pharmtech.com/after-valsartan-recalls-regulators-grapple-nitrosamine-contamination-apis (accessed 27 Mar 2019).

61 Ukwueze SE, Uliah C. Comparison of UV spectrophotometry and HPLC methods for the determination of pharmaceutical equivalence of clopidogrel tablet brands marketed in Nigeria. *Eur J Pharm Med Res* 2018;**5**:126–30.http://www.ejpmr.com/admin/download/article/MTUyNTA2Nzk2MC5wZGY=

62 Machairas G, Panderi I, Geballa-Koukoula A, *et al.* Development and validation of a hydrophilic interaction liquid chromatography method for the quantitation of impurities in fixed-dose combination tablets containing rosuvastatin and metformin. *Talanta* 2018;**183**:131–41. doi:10.1016/j.talanta.2018.02.068

63 Rebiere H, Kermaidic A, Ghyselinck C, *et al.* Inorganic analysis of falsified medical products using X-ray fluorescence spectroscopy and chemometrics. *Talanta* 2019;**195**:490–6. doi:http://dx.doi.org/10.1016/j.talanta.2018.11.051

64 Parr M, Joseph J. NDMA impurity in valsartan and other pharmaceutical products: Analytical methods for the determination of N-nitrosamines. *J Pharm Biomed Anal* 2019;**164**:536–49. doi:http://dx.doi.org/10.1016/j.jpba.2018.11.010

65 Banzi R. Regulatory response to contaminated valsartan. *BMJ* 2018;**362**:k3855. doi:http://dx.doi.org/10.1136/bmj.k3855

66 WHO pharmaceuticals Newsletter No.5 2018. *WHO* Published Online First: 2018.https://apps.who.int/iris/bitstream/handle/10665/275697/WPN-2018-05-eng.pdf?sequence=1&isAllowed=y (accessed 25 Sep 2019).

67 Tsutsumi T, Akiyama H. Analysis of an impurity, N-nitrosodimethylamine, in valsartan drug substances and associated products using GC-MS. *Biol Pharm Bull* Published Online First: 2019.https://www.jstage.jst.go.jp/article/bpb/advpub/0/advpub_b19-00006/_article/-char/ja/ (accessed 27 Mar 2019).

68 Gunasekaran PM, Chertow GM, Bhalla V, *et al.* Current status of angiotensin receptor blocker recalls. *Hypertension* 2019;**74**:1275–8. doi:http://dx.doi.org/10.1161/HYPERTENSIONAHA.119.13955

69 Zeng BS, Gu YR, Nguyen K, *et al.* Development of quantitative HPTLC-densitometry methods following a model process for transfer of TLC screening methods for pharmaceutical products containing moxifloxacin HCl, ofloxacin, amoxicillin trihydrate, acetylsalicylic acid + acetaminophen + caffe. *J Liq Chromatogr Relat Technol* 2019;**42**:324–9. doi:10.1080/10826076.2019.1585609

70 Antignac M, Bara Diop I, Macquart de Terline D, *et al.* Falsified and substandard cardiovascular drugs in Africa: a need for continued monitoring strategies. J. Glob. Health. 2019;**9**:010302. doi:10.7189/jogh.09.020302

71 Tamargo J, Journal-Cardiovascular GR-EH, undefined 2020. Low quality of some generic cardiovascular medicinal products represents a matter for growing concern. *academic.oup.com*https://academic.oup.com/ehjcvp/article-abstract/6/3/176/5566494

72 Ashames A, Bhandare R, AlAbdin SZ, *et al.* Public perception toward e-commerce of medicines and comparative pharmaceutical quality assessment study of two different products of furosemide tablets from community and illicit online pharmacies. *J Pharm Bioallied Sci* 2019;**11**:284–91. doi:10.4103/jpbs.JPBS_66_19

73 Snodin D., Elder DP. Short commentary on NDMA (N-nitrosodimethylamine) contamination of valsartan products. *Regul Toxicol Pharmacol* 2019;**103**:325–9. doi:http://dx.doi.org/10.1016/j.yrtph.2019.01.007

74 Defibrotide falsifie et contamine identifie dans les regions de l'OMS de l'Europe, Mediterranee orientale et du Pacifique occidental. https://www.who.int/fr/news/item/07-05-2020-medical-product-alert-n-5-2020 (accessed 16 Dec 2020).

75 Rojas-Cortés R. Substandard, falsified and unregistered medicines in Latin America, 2017-2018. *Rev Panam Salud Publica/Pan Am J Public Heal* 2020;**44**. doi:10.26633/RPSP.2020.125

76 Ballereau F, Prazuck T, Schrive I, *et al.* Stability of essential drugs in the field: Results of a study conducted over a two-year period in Burkina Faso. *Am J Trop Med Hyg* 1997;**57**:31–6. doi:10.4269/ajtmh.1997.57.31
