# Supplementary material for: The quality of medical products for cardiovascular diseases: a gap in global cardiac care
Source: BMJ Glob Health. 2021 Sep 14;6(9):e006523. doi: 10.1136/bmjgh-2021-006523 (PMC8442059; doi:10.1136/bmjgh-2021-006523)
Supplement: Supplementary data [file bmjgh-2021-006523supp017.pdf]

Supplementary file 17. Other articles discussing cardiovascular medical devices quality

Title	First author	Year
Pseudomalfunction of pacemaker due to a defective electrocardiograph [1]	N, Ali	1983
Defective intravenous cannula [2]	MP, Sinden	1995
Failure Rates of Leads, Pulse Generators, and Programmers Have Not Diminished Over the Last 20 Years: Formal Monitoring of Performance is Still Needed [3]	DT, Kawanishs	1996
Aspects généraux et réglementaires de la materiovigilance des stimulateurs et des défibrillateurs cardiaques [4]	L, Fauchier	2004
Heart-Valve Records Falsified [5]	M, Shiu	1991

1 Ali N, Bhatia S, N. A. Pseudomalfunction of pacemaker due to a defective electrocardiograph. *Am J Cardiol* 1984;**53**:373–4.http://ovidsp.ovid.com/ovidweb.cgi?T=JS&PAGE=reference&D=emed3&NEWS=N&AN=14215406

2 Sinden MP, Jayamaha JE, M.P. S. Defective intravenous cannula. *Anaesthesia* 1995;**50**:829–30.http://ovidsp.ovid.com/ovidweb.cgi?T=JS&PAGE=reference&D=emed5&NEWS=N&AN=125120386

3 Kawanishi DT, Song S, Furman S, *et al.* Failure Rates of Leads, Pulse Generators, and Programmers Have Not Diminished Over the Last 20 Years: Formal Monitoring of Performance is Still Needed. *Pacing Clin Electrophysiol* 1996;**19**:1819–23. doi:10.1111/j.1540-8159.1996.tb03232.x

4 Fauchier L, de Bouët du Portal H, Giraudeau C, *et al.* Aspects généraux et réglementaires de la matériovigilance des stimulateurs et des défibrillateurs cardiaques. *Ann Cardiol Angeiol (Paris)* 2005;**54**:38–43. doi:10.1016/j.ancard.2004.11.004

5 Shiu M, Dyer C. Heart-Valve Records Falsified. Br. Med. J. 1991;**303**:1222. doi:10.1136/bmj.304.6819.118-b
